# Systematic scRNA-seq screens profile neural organoid response to morphogens

**DOI:** 10.1038/s41592-025-02927-5

**Published:** 2025-12-15

**Authors:** Fátima Sanchís-Calleja, Nadezhda Azbukina, Akanksha Jain, Zhisong He, Ryoko Okamoto, Charlotte Rusimbi, Pedro Rifes, Gaurav Singh Rathore, Malgorzata Santel, Jasper Janssens, Makiko Seimiya, Benedikt Eisinger, Jonas Simon Fleck, Agnete Kirkeby, J. Gray Camp, Barbara Treutlein

**Affiliations:** 1 Department of Biosystems Science and Engineering, ETH Zürich, Basel, Switzerland; 2 Novo Nordisk Foundation Center for Stem Cell Medicine (reNEW), University of Copenhagen, Copenhagen, Denmark; 3 Institute of Human Biology, Roche Pharma Research and Early Development, Roche Innovation Center Basel, Basel, Switzerland; 4 Biozentrum, University of Basel, Basel, Switzerland

**Keywords:** Pattern formation, High-throughput screening, Gene regulatory networks, Gene expression, Neurogenesis

## Abstract

Morphogens direct neuroepithelial fates toward discrete regional identities in vivo. Neural organoids provide models for studying neural regionalization through morphogen exposure; however, we lack a comprehensive survey of how the developing human neuroepithelium responds to morphogen cues. Here we produce a detailed survey of morphogen-induced effects on the regional specification of human neural organoids using multiplexed single-cell transcriptomic screens. We find that the timing, concentration and combination of morphogens strongly influence organoid cell-type and regional composition, and that cell line and neural induction method impact the response to a given morphogen condition. We apply concentration gradients in microfluidic chips or increasing static concentrations in multi-well plates and observe different patterning dynamics in each scenario. Altogether, we provide a detailed resource on neural lineage specification that, in combination with deep learning models, can enable the prediction of differentiation outcomes in human stem-cell-based systems.

## Abstract (web summary)

This Resource provides a detailed atlas of morphogen-induced responses in human neural organoids.

## Main

The elaborate architecture and functionality of the human central nervous system (CNS) arises from tightly coordinated patterning events initiated in early stages of development. After gastrulation, ectodermal cells giving rise to non-neural tissues maintain their identity via paracrine bone morphogenetic protein 4 (BMP4) signaling. The neural plate emerges from the ectoderm through BMP inhibition and fibroblast growth factor (FGF) activation signals, ultimately giving rise to the CNS. Meanwhile, at the edge of the neural plate, ectodermal cells with intermediate BMP and high FGF and WNT signaling activity form the neural crest, precursor of the peripheral nervous system. While these lineages are established, a unique set of morphogen concentration gradients interact in space and time to guide neural tube regionalization along the anteroposterior (AP) and dorsoventral (DV) axes^1–3^.

Current knowledge of CNS regionalization mostly originates from studies using non-human animal models with divergent cytoarchitecture, cell behavior and gene expression patterns compared to humans^4–7^. However, the advent of stem cell-based neural differentiation methods has enabled the study of human-specific neurodevelopment in vitro^4,5,8–12^. While monolayer neural cultures present basic rosette-like arrangements, neural organoids provide a three-dimensional environment where cells self-organize cytoarchitectural and morphogenetic features with similarities to the primary human tissue, such as cortical layering and folding as well as interneuron migration^13,14^. Hence, human pluripotent stem (hPS) cell-derived neural organoids provide the unique opportunity to systematically explore the effect of morphogens on neuroepithelial development and brain regional patterning.

Numerous approaches have been developed to generate reproducibly patterned neural organoids, such as organoids with intrinsic inducible organizers^15^, self-folding systems with DV patterning^16^, and a wide range of protocols for the generation of regionalized neural organoids of forebrain, midbrain, hindbrain or retinal-like tissue fates^17–22^. Although these advances have increased our understanding of neural tissue regionalization and consolidated neural organoids as a leading model system for neurodevelopmental studies^13,23^, it is still challenging to integrate the findings obtained in each study and evaluate their relevance for human-specific biology. Different guided organoid protocols aimed toward the same brain region can produce different outcomes^24^, as they use different culture settings that might affect tissue architecture, regional patterning, cell-type diversity and maturation rates.

The development of new protocols is still inefficient: the assessment of different culture conditions typically relies on bulk measurements of a few marker genes with low organoid throughput, and their validation is often performed after several months in culture, when they are predominantly composed of mature neurons. Besides slowing down progress in the field, this approach has neglected the study of early neuroepithelial patterning, which is key for the successful generation of regionally patterned and functionally mature neural organoids.

To unify our understanding of early neural regionalization, we implement a systematic approach to assess morphogen effects on developing human neural organoids. We supply patterning molecules at multiple time points, concentrations and combinations, and use highly multiplexed single-cell RNA-sequencing (scRNA-seq) with sample barcoding^25,26^ to assess neural progenitor regionalization after 3 weeks in culture. Analysis of this time point captured a high diversity of developmental stages, ranging from pluripotent stem cells and neuroectodermal states to neural progenitors and neurons. We show that the delivery of sonic hedgehog (SHH), WNT, FGF8, retinoic acid (RA), BMP4 and BMP7 pathway modulators in short pulses over different time windows induces substantial changes in organoid composition. Increasing concentration steps in enlarged treatment windows modulate the proportion of each regional identity within the same tissue. Guided by this analysis, we use combinations of morphogens to steer axial and regional fates in a more precise manner. Our experiments reveal substantial variability across hPS cell lines and neural induction methods. Finally, we show that an SHH morphogen gradient and discontinuous SHH morphogen steps similarly recapitulate DV patterning of the human forebrain. Altogether, our work provides an in-depth survey of morphogen-induced patterning of human neural organoids and serves as a reference to accelerate development of optimized organoid models.

## Results

### A single-cell screening of morphogen patterning conditions in human neural organoids

**Fig. 1:**
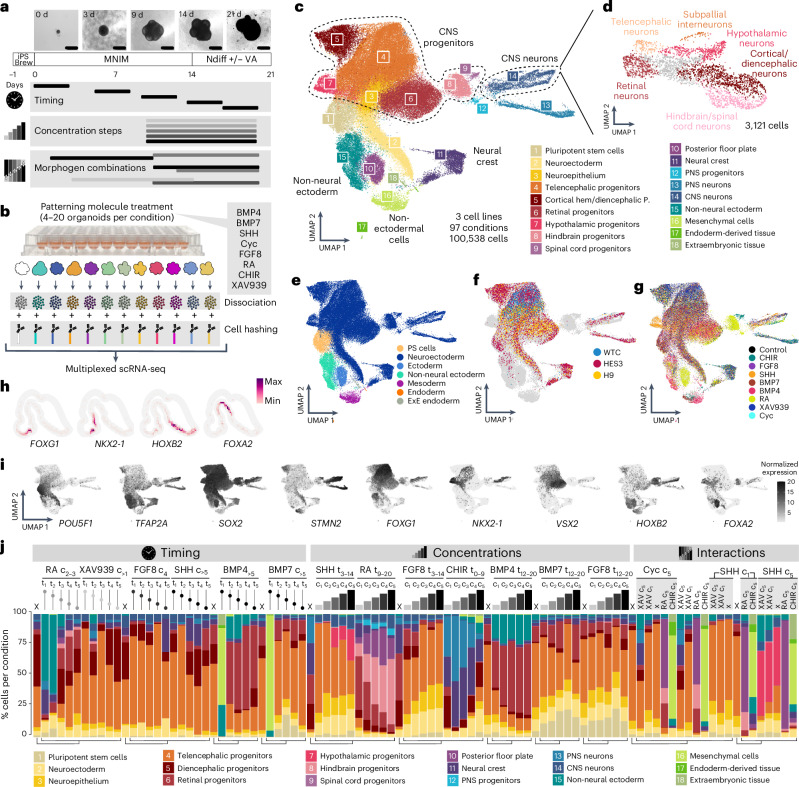
A single-cell transcriptomic atlas of human neural organoid patterning. **a**, Experimental timeline showing morphogen treatment modalities in this study, an overview of the organoid culture protocol used and representative bright-field images at different developmental stages (WTC cell line). Scale bars, 500 µm. A full overview of all experiments performed can be found in Extended Data Fig. 1 and Supplementary Fig. 1. Ndiff +/− VA, neural differentiation media with or without vitamin A. **b**, Overview of the experimental approach to obtain multiplexed scRNA-seq readouts from treated organoids. **c**, RSS-based UMAP projection of all cells in the dataset, including three hPS cell lines (HES3, H9 and WTC) in different proportions, colored by cluster identity. iPS Brew, StemMACS iPS Brew XF. **d**, RSS-based UMAP projection of subclustered CNS neurons, showing different regional identities in the same colors as their corresponding progenitors shown in **c**. P., progenitors. **e**, Whole-dataset RSS-UMAP projection colored by germ layer, showing off-target cells mostly generated by morphogen treatments. PS cells, pluripotent stem cells; ExE endoderm, extraembryonic endoderm. **f**, RSS-UMAP projection highlighting untreated (control) cells from each cell line of origin. Cells from morphogen treatment conditions are shown in gray. **g**, Same projection highlighting cells treated with a single morphogen and control cells. Cells from combined morphogen treatments are colored in gray in the background. **h**, Voxel maps showing the normalized expression of regional marker genes in the developing human brain dataset published in Braun et al.^28^. **i**, Feature plots for representative marker genes (*POU5F1*, stem cells; *TFAP2A*, non-neural ectoderm or hindbrain; *SOX2*, neural stem cells; *STMN2*, neurons; *FOXG1*, telencephalon; *NKX2-1*, ventral telencephalon; *VSX2*, retina; *HOXB2*, hindbrain; *FOXA2*, floor plate). **j**, Bar plots showing the cell-type composition of each condition for HES3-derived organoids exposed to a single time point, concentration steps and an example subset of combinatorial morphogen treatments. Concentrations are indicated by c_1_–c_5_ and treatment windows by t_0–21_, indicating the start and end of morphogen exposure in days. Lines with dots represent timing experiments (single morphogen pulse); the dot represents the time frame when the treatment took place (up is early, down is late). Concentration experiments are represented by progressively taller bars. The shading of the dots and bars in timing and concentration experiments represents the concentration used (darker shading means higher concentration). ‘X’ indicates untreated (control) organoids. Correspondences between controls and experimental conditions are indicated by the tree below the bar plots. On the right, combinatorial experiments (‘Interactions’) are labeled with the AP morphogen (vertical) and the DV morphogen (horizontal); for simplicity, only concentrations are indicated (refer to Supplementary Fig. 1 for timing information) and FGF8 combinations are not shown. PNS, peripheral neural system.

**Extended Data Fig. 1:**
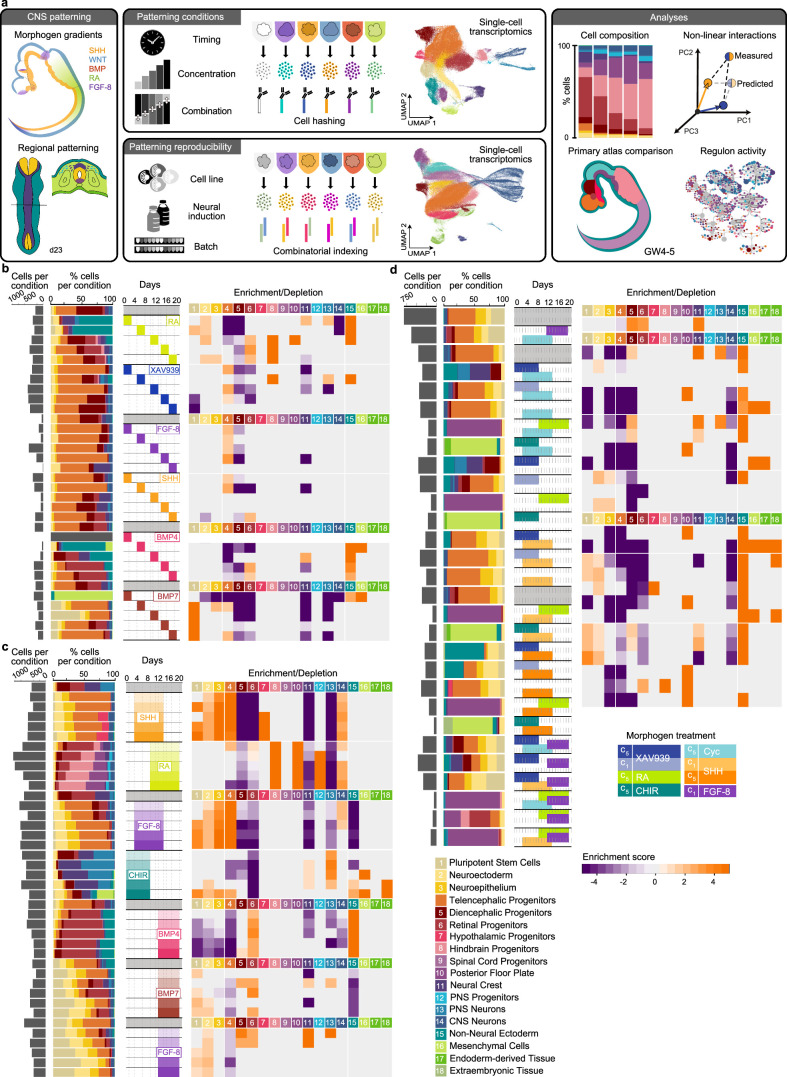
Overview of conditions and results of patterning condition screening. **a**, Overview of the experimental setup and downstream analyses. **b-d**, Barplots (left) show total number of cells sequenced and percentage of cells in each cluster per condition in the morphogen exposure timing (**b**), concentration (**c**), and combination (**d**) experiments. Heatmap shows enrichment (orange) and depletion (purple) of each cluster across conditions (right) in comparison to corresponding control, untreated organoids from the same experiment. All results correspond to the cell line HES3.

We established an experimental workflow to systematically test the timing, concentration and combinations of patterning molecules using cell hashing-based multiplexed scRNA-seq. Neural organoids were generated in 96-well plates, where pluripotent stem cells (WTC, H9 and HES3 lines; the latter modified to report the expression of the regional marker NKX2-1) were aggregated into embryoid bodies and cultured in minimal neural induction media (MNIM) to induce neuroectodermal fates (Fig. 1a). Developing organoids were then subjected to a panel of morphogen treatments with varying timing, concentration and combination of the molecules CHIR99021 (CHIR, WNT pathway activator), XAV939 (WNT pathway inhibitor), rh-SHH/CS24II (SHH) and purmorphamine, FGF-8B (FGF8), RA, rhBMP-4 (BMP4), rhBMP-7 (BMP7) and cyclopamine (Extended Data Fig. 1a–d and Supplementary Figs. 1 and 2). In each batch, one condition did not receive any morphogen pathway modulator, but was exposed to the same base media and, therefore, served as control. On day 21, multiple neural organoids of each condition (Supplementary Fig. 1) were pooled and dissociated into single-cell suspensions, followed by cell hashing with barcoded antibodies^25^ and processing for single-cell transcriptome sequencing (3′ mRNA, 10x Genomics; Fig. 1b). Altogether, 100,538 single cells from 97 morphogen treatment conditions were analyzed, including untreated organoids (one control condition per 96-well plate or organoid batch).

**Extended Data Fig. 2:**
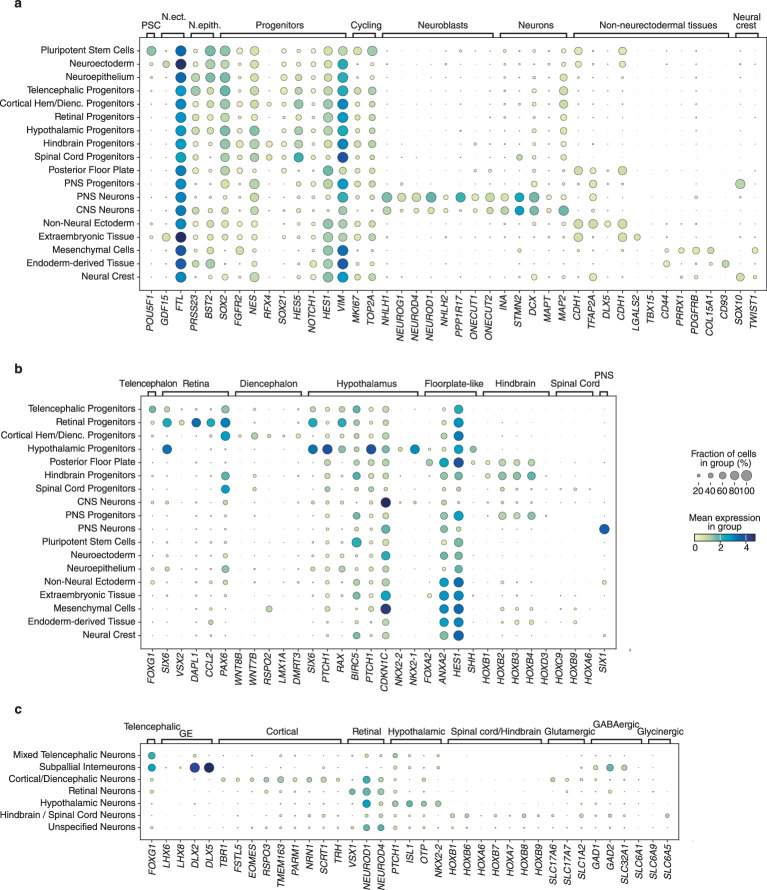
Annotation of single-cell dataset resulting from the patterning condition screening. Dotplots showing cluster marker expression for the entire morphogen screen dataset (including 3 cell lines H9, HES3 and WTC), showing cell class markers (**a**) and brain region markers (**b**). PSC., pluripotent stem cells; N.ect, Neuroectoderm; N.epith., Neuroepithelium; Dienc., Diencephalon; PNS, peripheral neural system; CNS, central neural system. **c**, Dotplot, showing marker expression for CNS neurons. GE, ganglionic eminence.

We integrated the data using reference similarity spectrum (RSS)^27^ comparison to an early developing human brain cell atlas ref. ^28^ and embedded the dataset with uniform manifold approximation and projection (UMAP)^29^ (Fig. 1c–e). Analysis of cluster marker gene expression (Extended Data Fig. 2a–c and Supplementary Table 1) revealed cell identities including diverse CNS progenitors and neurons of telencephalon, diencephalon, retina, hypothalamus, hindbrain and spinal cord domains, as well as neural crest, non-neural ectoderm, mesoderm, endoderm and extraembryonic tissue (Fig. 1e). Further subclustering of CNS neurons revealed different regional identities consistent with the identities observed in CNS progenitors (Fig. 1d). We found that organoid cells from all hPS cell lines predominantly localized to neural clusters in the absence of any morphogen treatment (Fig. 1f), whereas treated organoids generated distinct clusters that resembled neural regions absent in control organoids, such as hindbrain and hypothalamus, as well as other non-neural tissues (Fig. 1g–i).

The single-cell transcriptomic readout allowed quantification of organoid cell-type composition for each morphogen treatment (Fig. 1j). We measured the heterogeneity of each patterning condition using the Shannon diversity index, where lower scores indicate lower cell-type diversity. While few conditions showed relatively homogeneous cell-type compositions (high cyclopamine or late supply of low FGF8), most conditions displayed an array of cellular identities spanning differentiation states between progenitors and neurons patterned to different brain regions (Fig. 1j and Supplementary Table 2). Interestingly, higher morphogen concentrations did not generally promote more homogeneous cell-type composition in the organoids, but instead generated new cell types that incrementally replaced the initial identities across the concentration conditions. Altogether, we provide a single-cell transcriptomic reference of human neural organoid patterning to explore principles of cell fate acquisition in response to extrinsic morphogen exposure.

### Effect of morphogen timing and concentration on regional cell identities in developing neural organoids

**Fig. 2:**
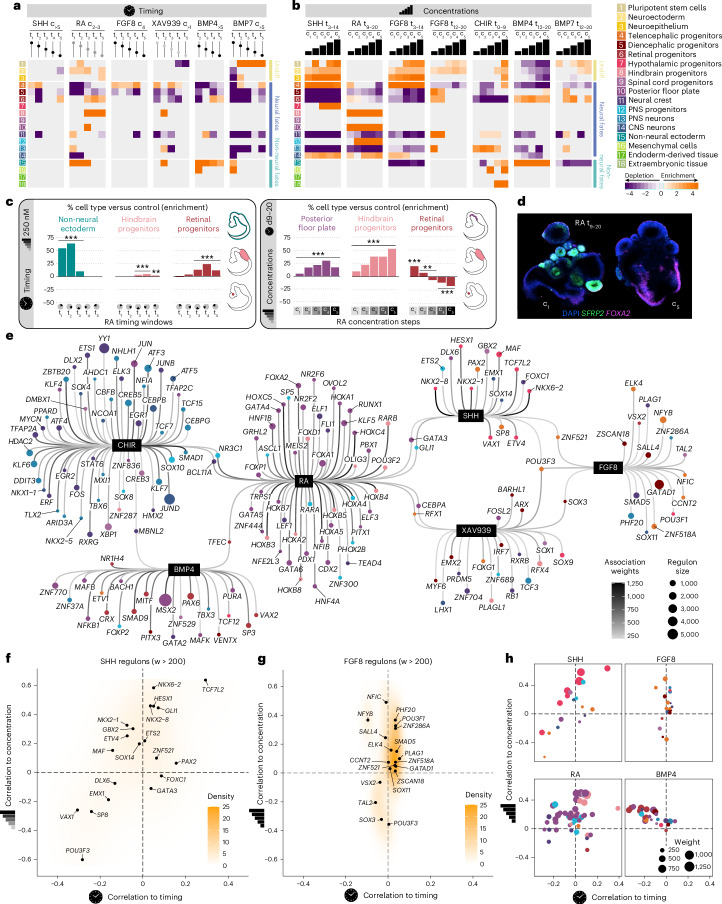
Morphogen timing and concentration determine regional cell identities in developing neural organoids. All results in this figure refer to the hES cell line HES3. **a**,**b**, Heat maps showing enrichment/depletion scores for timing (**a**) and concentration (**b**) experiments in HES3-derived organoids. The shading of dots and bars on top of the heat map indicates the used dose, and the dots along a line represent the time point of patterning molecule addition. **c**, Effects of different RA treatment windows (left) and concentration steps (right) on the abundance of retinal and hindbrain progenitors, non-neural ectoderm and posterior floor plate in 21-day-old neural organoids. *P* values were derived using two-sided Fisher’s exact test with Bonferroni correction. ***P value < 0.001, ** 0.001 < *P* value < 0.01. Exact *P* values can be found in Supplementary Table 3. **d**, HCR in situ hybridization for *SFRP2* (retinal marker gene) and *FOXA2* comparing RA treatment conditions c_1_ and c_5_. DAPI, 4′,6-diamidino-2-phenylindole. **e**, Inferred regulon network for all tested patterning molecules. Each black node represents a morphogen that is connected to a number of associated regulons (showing correlating activity to morphogen treatment). The size of each regulon node indicates the relative size of the regulon, and the color refers to the cluster where that regulon registers maximum activity (excluding non-neural and undifferentiated clusters). **f**, Scatterplot of SHH-associated regulon correlation to timing and concentration, showing wide dependency on both variables. **g**, Scatterplot of FGF8-associated regulon correlation to timing and concentration, showing high dependency on concentration and low dependency on timing. For **f** and **g**, οnly regulons with weights above 200 are shown in the plot. **h**, Summary scatterplot showing how modulating the timing and concentration of each morphogen can be used to activate regulons associated with different cell types. Each panel shows the top regulons associated with each morphogen and how their activity correlates with changes in morphogen timing and concentration. Each regulon is one dot in the plot. Dot size indicates regulon weight and color indicates the cluster that the regulon is most enriched in (that is, the main cell type possibly controlled by that regulon).

**Extended Data Fig. 3:**
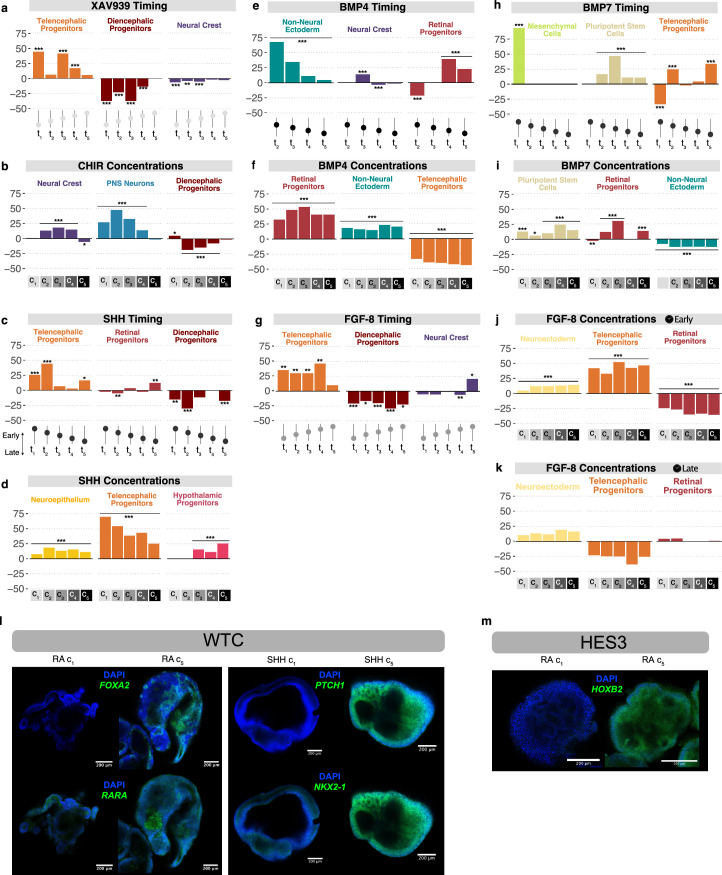
Regulation of neural and non-neural cell fates by morphogen timing and morphogen concentrations. **a-k**, Most significantly enriched or depleted clusters for each morphogen after single time point pulses (Timing) or prolonged exposure to concentration steps (Concentrations) in HES3-derived organoids. P-values were derived using two-sided Fisher’s exact test with Bonferroni correction. *** p-value < 0.001, ** 0.001< p-value < 0.01, * 0.01 < p-value < 0.05. Exact p-values can be found in Supplementary Table 3. l, HCR in situ hybridization for FOXA2 and RARA comparing RA treatment conditions c1 and c5 (left) and HCR in situ hybridization for PTCH1 and NKX2-1 comparing SHH treatment conditions c1 and c5 (right) for WTC cell line. m, HCR in situ hybridization for HOXB2 comparing RA treatment conditions c1 and c5 for HES3 cell line. In panels **l** and **m**, images are representative of 3 organoids per condition.

**Extended Data Fig. 4:**
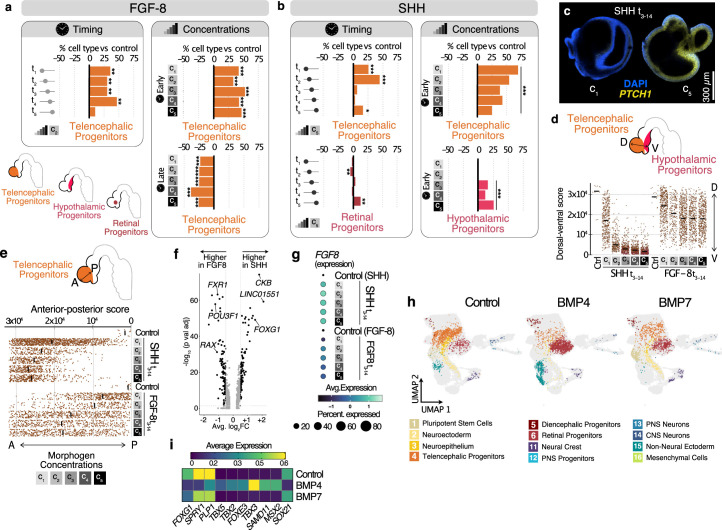
Comparing the effects of FGF8 versus SHH and BMP4 versus BMP7 treatment. Results shown correspond to the lines HES3 (single-cell RNA-seq analysis) and WTC (HCR image). **a**, Effects of different FGF8 treatment windows (left) and concentration steps (right) at early (day 3-14) and late (day 12-20) time windows on the abundance of telencephalic progenitor populations at 21 days. Bottom left: Spatial representation of the clusters enriched by FGF8 and SHH in a schematic representation of a ~ 4 GW human embryo. **b**, Effects of different SHH treatment windows (left) and concentration steps (right) on the abundance of retinal, telencephalic and hypothalamic progenitor populations in 21-day-old neural organoids. P-values were derived using two-sided Fisher’s exact test with Bonferroni correction. For panels **a** and **b**, *** p-value < 0.001, ** 0.001< p-value < 0.01, * 0.01 < p-value < 0.05. Exact p-values can be found in Supplementary Table 3. **c**, HCR in situ hybridization for PTCH1 (hypothalamic marker gene and target gene of SHH signaling) comparing SHH treatment conditions c1 and c5. Cell line = WTC. Images are representative of 3 organoids per condition. **d**, Dorso-ventral score values of forebrain single cells for different concentration steps of SHH and FGF8 (both given on days 3-14) with their corresponding controls showing higher ventralization in SHH-treated organoids, which reach the hypothalamic compartment. Horizontal bars depict the median dorso-ventral score across all cells categorized as telencephalic and hypothalamic progenitors for each condition. n = 12 for Ctrl (SHH experiment), n = 424 for SHH c1, n = 271 for SHH c2, n = 379 for SHH c3, n = 380 for SHH c4, n = 361 for SHH c5, n = 17 for Ctrl (FGF-8 experiment), n = 446 for FGF-8 c1, n = 239 for FGF-8 c2, n = 526 for FGF-8 c3, n = 407 for FGF-8 c4 and n = 472 for FGF-8 c5. **e**, Anteroposterior scores for telencephalic progenitors treated with SHH or FGF8 concentration steps on days 3-14. Vertical bars depict the median dorso-ventral score across all cells categorized as telencephalic progenitors for each condition. n = 12 for Ctrl (SHH experiment), n = 1165 for SHH c1, n = 269 for SHH c2, n = 274 for SHH c3, n = 304 for SHH c4, n = 356 for SHH c5, n = 17 for Ctrl (FGF-8 experiment), n = 439 for FGF-8 c1, n = 238 for FGF-8 c2, n = 523 for FGF-8 c3, n = 406 for FGF-8 c4, and n = 470 for FGF-8 c5. **f**, Volcano Plot showing the differentially expressed genes between the telencephalic progenitors of FGF-8 and SHH conditions, promoting the greatest increase in telencephalic progenitors (c3 on days 3-14 for FGF-8, and c2 on days 3-14 for SHH). Differentially expressed genes were obtained by applying the Wilcoxon rank sum test for differential expression analysis. P-values were then subjected to Bonferroni correction for multiple testing. **g**, Endogenous FGF8 expression in telencephalic progenitors in response to SHH or FGF8 concentration steps applied at the same time window. SHH expression is not shown, as it consistently yielded zero values. **h**, UMAP representation of cell fateshifts under BMP4 and BMP7 treatment in comparison to control. Only cells from the BMP concentration steps experiment are shown and are coloured by cluster. Legend with relevant clusters is shown below. **i**, Heatmap showing differentially expressed genes in ectodermal cells from control, BMP4 or BMP7 conditions.

To identify effects of each morphogen at the cell-type level, we calculated cluster enrichment/depletion scores for each treatment (Fig. 2a–c, Extended Data Figs. 3a–k and 4a,b and Supplementary Table 3). Most morphogen pathway manipulations resulted in enrichment or depletion of one or more clusters. We observed the most significant changes in cell-type composition in the morphogen concentration experiments, where organoids were exposed to morphogens for a longer time (Fig. 2b). However, a single pulse of certain treatments also showed remarkable effects, such as RA promoting non-neural ectoderm at early time points but retinal and hindbrain progenitors when supplied at later time points (Fig. 2a,c). Interestingly, late and long exposure (days 9–20) of a low concentration (c_2–3_) of RA promoted the emergence of retinal progenitors, whereas higher concentration of RA progressively enriched for hindbrain and posterior floor plate progenitors (Fig. 2c). In situ hybridization chain reaction (HCR) stainings confirmed the presence of more retinal progenitors (*SFRP2*^+^) at lower RA concentrations and more ventralized and posterior fates (*FOXA2*^+^) at higher RA concentrations (Fig. 2d and Extended Data Fig. 3l,m).

Inhibition of the WNT pathway (XAV939) at early and intermediate time points (days 0–3, 10–13 and 14–17) led to a significant enrichment in telencephalic progenitors (Fig. 2a and Extended Data Fig. 3a). Activation of the WNT pathway via exposure to CHIR caused strong dorsalization and posteriorization, as shown by the production of neural crest fates and peripheral nervous system neurons (Fig. 2b and Extended Data Fig. 3b). At the highest concentrations, CHIR additionally guided cells toward non-neural identities such as mesenchyme (Fig. 2b).

BMP4 and BMP7 often display interchangeable roles in neural patterning^30^; therefore, we tested them in equimolar steps to dissect their roles. Both factors prevented neural specification when supplied in early time windows, consistent with their role in vivo^30^ (Fig. 2a and Extended Data Fig. 3e,h). When provided on days 0–3, BMP4 led to organoid disintegration, while BMP7 induced a nearly 90% enrichment in mesenchymal cells (Extended Data Fig. 3h). After day 3, BMP4 induced non-neural ectoderm (NNE) formation and progressively guided toward retina cell fates at later time points (Fig. 2a and Extended Data Fig. 3e). Instead, BMP7 guided cells toward pluripotency, even when added at later time points (Extended Data Fig. 3h). When supplying BMP4 over an extended period of time (days 12–20), increase in concentration led to an increase in the percentage of retinal progenitors, while still producing NNE cells (Fig. 2b and Extended Data Fig. 3f). BMP7 also showed a dose-dependent induction of retinal progenitor fate, although less efficiently than BMP4 (Fig. 2b and Extended Data Fig. 3i). Overall, treatment with both BMP4 and BMP7 generated the same cell types as control organoids, but BMP4 significantly altered their proportions (Fig. 2b and Extended Data Fig. 4h). We calculated differential expression between control, BMP4 and BMP7 conditions (Extended Data Fig. 4i) and found very similar expression patterns in control and BMP7-treated organoids, whereas BMP4-treated cells activated *MSX2* and *TBX* genes, implicated in neural crest development and dorsal neural tube formation^31,32^. BMP4 also activated the expression of *FOXE3* and *SAMD11*, which are essential for optic cup morphogenesis^33,34^, consistent with the promotion of retinal fates.

SHH and FGF8 both induced telencephalic fates when supplied in short pulses at specific time points (Extended Data Figs. 3c,g and 4a,b). FGF8 maintained this activity over a longer time window, while the SHH patterning window was more restricted to earlier time points. Exposing organoids to FGF8 for a longer duration led to depletion of telencephalic fates at later time points (days 12–20), in contrast to enrichment in early time points (days 3–14; Extended Data Figs. 3j,k and Fig. 4a). Late single-pulse SHH exposure favored the production of retinal progenitors together with a slight enrichment in telencephalic identities (Extended Data Fig. 4b). Sustained and progressively higher SHH concentrations induced a transition from telencephalic to hypothalamic progenitors (Extended Data Figs. 3d and 4b,c), consistent with the spatial concentration gradient of SHH in vivo^35–37^.

We next compared FGF8 and SHH influence on DV and AP patterning within the telencephalon. We calculated DV and AP scores for each cell using marker genes varying along these axes identified from the primary human brain (Extended Data Fig. 4d,e). We observed that SHH had stronger ventralizing and anteriorizing effects than FGF8, generating hypothalamic floor plate cells at the highest concentrations, while also generating more highly anteriorized telencephalic progenitor cells (Extended Data Fig. 4d–f). This finding is surprising since it has been shown in the mouse that FGF8 is secreted from the anterior neural ridge to promote anteriorization of the developing telencephalon, potentially suggesting a differential role of FGF8 in mouse and human brain patterning^38^. Interestingly, SHH exposure induced FGF8 expression, but FGF8 treatment did not induce SHH nor FGF8 expression (Extended Data Fig. 4g).

### Morphogen conditions differentially activate transcription factor regulons in developing neural organoids

We next explored how morphogens impact gene regulatory networks underlying human neural organoid development (Fig. 2e and Supplementary Table 4). Using the screening data for the HES3 embryonic stem (ES) cell line, we linked each morphogen treatment to regulons^39^—a transcription factor and a set of predicted downstream target genes—and assessed regulon specificity by comparing regulon activity across cell types. Notably, some regulons were associated with two or more morphogens, apparent as interconnected nodes in the network graph, such as regulons linked to both SHH and RA (for example, *GATA3*, *GLI1*).

**Extended Data Fig. 5:**
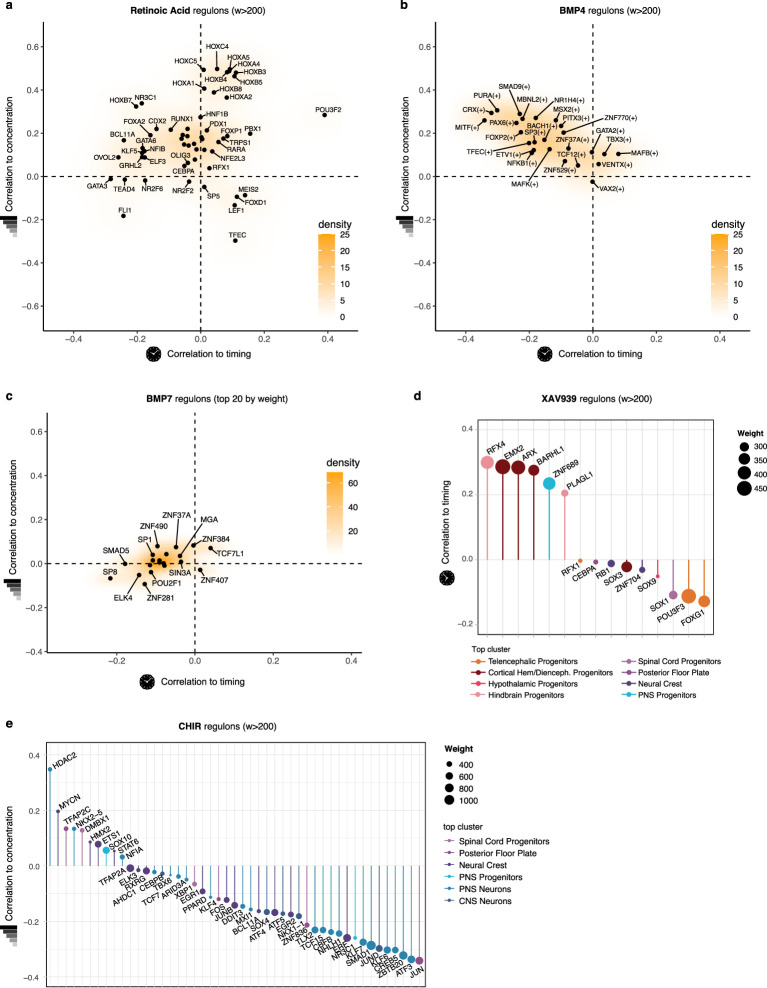
Correlations between regulon activity and morphogen timing and concentration. All results correspond to the hESC line HES3. (**a-c**) Scatterplot showing the relationship between correlation to timing (x-axis) and concentration (y-axis) of morphogen-associated regulons, with selected regulons labeled. **a,b**, Scatterplots of RA- and BMP4-associated regulon correlation to timing and concentration. **c** Scatterplot of the top 20 weighted BMP7-associated regulons correlation to timing and concentration, showing roughly equal dependency on concentration and timing. **d**, Lollipop plot showing the correlation of regulon activity (weights>200) to XAV939 treatment timing. **e**, Lollipop plot showing the correlation of regulon activity (weights>200) to CHIR concentrations. In **d** and **e**, the size of the lollipop circle represents the weight of the corresponding association to the morphogen, and the color represents the cluster where the regulon is most enriched. The top regulons with associations to timing surpassing weights of 200 are shown.

We next examined the dependency of regulon activity on morphogen timing and concentration, finding both positive and negative correlations (Fig. 2f–h and Extended Data Fig. 5). While SHH-linked and RA-linked regulons showed wide dependency on both timing and concentration (Fig. 2f and Extended Data Fig. 5a), FGF8-linked regulons showed strong dependence only on concentration (Fig. 2g), and BMP4-linked and BMP7-linked regulons were more dependent on timing than concentration (Extended Data Fig. 5b,c). Regulon associations for BMP7 were weak, not surpassing thresholds (weights above 200).

Notably, this analysis shows that tuning morphogen timing, morphogen concentration or both factors can activate different developmental programs in a targeted manner. For example, SHH-induced activation of the *NKX2-1* regulon (key for the formation of the medial ganglionic eminence or MGE) occurred homogeneously across treatment windows but required higher SHH concentrations, while SHH-induced activation of the *TCF7L2* regulon (essential for thalamic development) occurred only at later time points and even higher concentrations (Fig. 2f,h). Interestingly, retinal regulons *VSX2* and *VAX2* were active independent of FGF8 and BMP4 treatment time point and concentration, respectively (Fig. 2g,h and Extended Data Fig. 5b). RA induced *FOXA2* regulon activity at early treatment windows with little dependency on RA concentration. Instead, *HOX* genes were induced by RA in a concentration-dependent manner. *HOXB8* and *HOXA2* regulons were activated at similar RA concentrations, while *HOXB7* was activated with earlier RA exposures (Extended Data Fig. 5a).

Inhibition of the WNT pathway using XAV939 mildly modulated regulon activity in a time-dependent manner, as shown by *FOXG1* and *POU3F3* correlating with early exposure and *EMX2*, *ARX* and *BARHL1* regulons correlating with late exposure times, respectively (Extended Data Fig. 5d). In contrast, WNT pathway activation using CHIR showed a clear dependency on morphogen concentration. Most CHIR-associated regulons were related to neurogenesis and showed negative correlation to CHIR concentration, indicating a possible suppression of neural fates. Consistently, regulons positively correlated with CHIR concentration were associated with alternative non-neural programs like neural crest differentiation (for example, *SOX10*; Extended Data Fig. 5e).

These analyses demonstrate that modulating morphogen timing and concentration can selectively modulate distinct subsets of morphogen-associated regulons that drive emergence of distinct cell fates.

### A combinatorial patterning screening reveals morphogen interactions

**Fig. 3:**
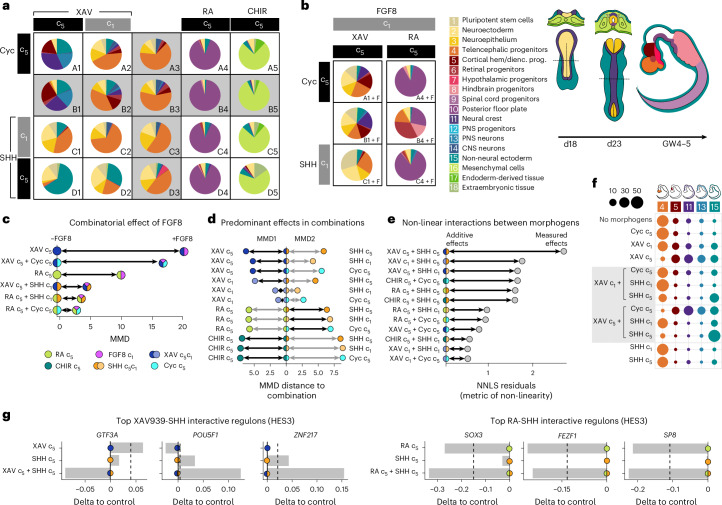
Morphogen combinations regulate neural cell fates. All results in this figure refer to the hES cell line HES3. **a**, Pie charts showing cell-type composition of each condition of the morphogen treatment panel in HES3-derived organoids. c_5_ and c_1_ refer to the highest and lowest concentration, respectively, used for the corresponding morphogens, consistent with previous experiments. Each condition has a coordinate indicated in the lower-right corner. **b**, Pie charts of cell-type composition for treatment conditions A1–C1 and A4–C4 supplemented with FGF8 (indicated as ‘+F’ in the coordinate name). Cell-type names and color correspondence for **a** and **b** are indicated at the bottom and right of the panels. dienc. prog., diencephalic progenitors. **c**, Quantification of MMD distance between each morphogen treatment and its equivalent with FGF8 in addition. The colors of each dot represent the morphogens and concentrations that make up the treatment, and stay consistent through **с**–**e** and **g**. **d**, Quantification of MMD distances between each morphogen treatment and their respective combination. Smaller MMD distances between two conditions imply more similarity. **e**, Non-negative least squares (NNLS) residuals are used to measure deviation from a hypothetical additive effect of the two composing morphogens. Quantification of NNLS residuals is used to measure deviation from a hypothetical additive effect of the two composing morphogens. Higher values indicate non-linearity, non-additive effects between two morphogens. **f**, Dot plot summarizing the most substantial variations in cell-type proportions for treatment with different doses of XAV939 and SHH, both alone and in combination, versus their control condition (no morphogens). **g**, Bar plots showing regulon activation relative to control in selected conditions where interactive effects were detected. Dashed lines represent the expected regulon activity in case of additive effects. The colors of circles in the baseline represent the morphogen condition according to the colors shown at the bottom of **c**.

To probe relevant morphogen combinations, we selected timing and concentration conditions and designed a combination scheme to reach most domains of the CNS (Fig. 3a). XAV939, RA and CHIR were used to pattern along the AP axis, whereas cyclopamine and SHH were applied to modulate along the DV axis. We designed additional conditions including FGF8 together with other morphogens that may generate anterior neural ridge or midbrain–hindbrain boundary domains (Fig. 3b). In addition, we performed this experiment with two different hPS cell lines: HES3 (human embryonic stem (hES) cell, XX karyotype) and WTC (human induced pluripotent stem (hiPS) cell, XY karyotype). We note that these experiments are performed in MNIM without dual SMAD inhibition.

**Extended Data Fig. 6:**
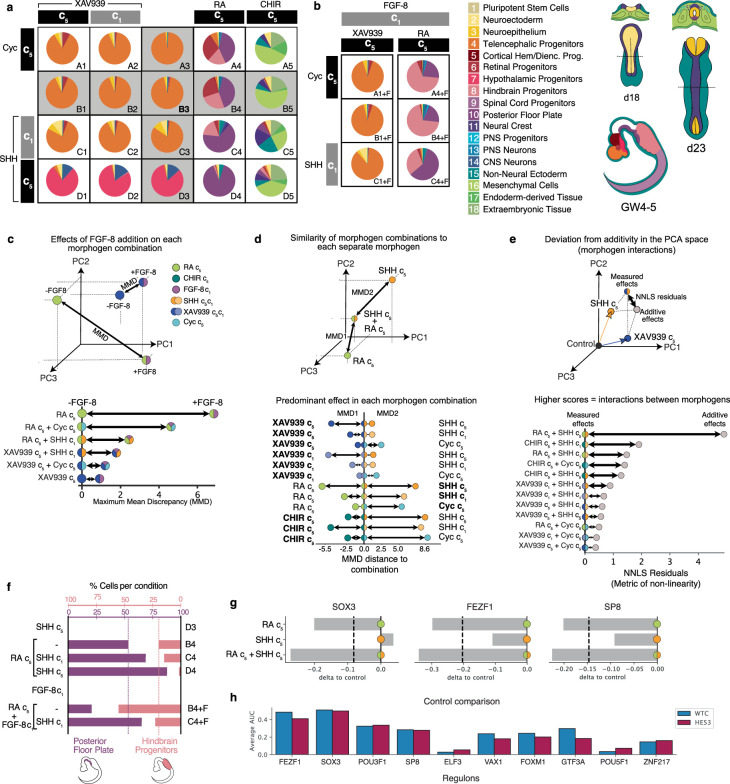
Morphogen combinations regulate neural cell fates (WTC). All plots in this figure are related to WTC. **a**, Pie charts indicating cell types distributions of each coordinate of the morphogen treatment panel. Pie charts with gray background correspond to single morphogen treatment. B3 serves as a control, without any morphogens added, and is calculated as the average of all control conditions for each organoid batch. **b**, Pie charts indicating cell types distributions of each coordinate of the morphogen treatment panel with addition of FGF8 on top of the corresponding morphogens. **c**, Top, example projection of MMD distances between conditions with and without FGF8 in a common PCA space. The colors of each dot represent the morphogens and concentrations that compose the treatment. MMD, Maximum Mean Discrepancy. Bottom, quantification of MMD distance between each morphogen treatment and its equivalent with FGF8 on top. **d**, Top, example projection of MMD distances between treatments using two separate morphogens and their respective combination in a common PCA space. Bottom, quantification of MMD distances between each morphogen treatment and their respective combination. Smaller MMD distances between two conditions imply more similarity. **e**, Top, example projection of single morphogen treatments, the expected result of their additive combination and the observed result of their combination in a common PCA space. Non-Negative Least Squares (NNLS) Residuals indicate whether the combination of morphogens deviates from the additive model of single morphogen-treated conditions. Bottom, quantification of NNLS Residuals for each combination of morphogens. **f**, Enrichment of hindbrain vs. posterior floor plate in different conditions under RA, SHH and FGF8 treatment. The basal levels of posterior floor plate and hindbrain progenitors under treatment with only RA are shown with a discontinued line in purple or pink, respectively. **g**, Bar plots showing regulon activation relative to control in RA- and SHH-high conditions where interactive effects were detected. Dotted lines represent the expected regulon activity in case of additive effects. The colors of circles in the baseline represent the morphogen condition and follow the same code as panels **c**-**e**. **h**, Comparison of baseline activation of each interactive regulon in control organoids (without morphogens) across hPSC lines.

Different morphogen combinations displayed varying degrees of consistency across the two cell lines. Treatments including CHIR consistently induced non-neural fates. RA consistently induced the posterior floor plate in both cell lines, although WTC also produced hindbrain and retinal progenitors in low SHH signaling conditions (Fig. 3a and Extended Data Fig. 6a). XAV939 anteriorized WTC organoids, as shown by abundant telencephalic progenitors (Extended Data Fig. 6a). However, HES3 organoids responded to XAV939 treatments by increasing non-neural ectoderm and neural crest-derived fates (Fig. 3a). The addition of FGF8 to XAV939-treated organoids enriched for neural and undifferentiated states in HES3 (Fig. 3b), but no significant changes occurred in WTC organoids (Extended Data Fig. 6b). Meanwhile, treatment of HES3 organoids with FGF8 and RA notably increased the fraction of retinal progenitors (Fig. 3b). In WTC organoids, retinal progenitors were already abundant with RA treatment alone, and the addition of FGF8 turned them into hindbrain progenitors (Extended Data Fig. 6b).

To better understand the contribution of each morphogen to cell-type compositions in each cell line, we summarized each sample based on treatment and cell line (Methods; pseudo-bulk analysis of conditions) and projected them in a three-dimensional principal component analysis (PCA) space. We then measured maximum mean discrepancy (MMD) values between conditions with FGF8 and their equivalents without FGF8, as a measure of FGF8’s effects on the preexisting conditions (Fig. 3c and Extended Data Fig. 6c). We found that FGF8 induced the largest effects on cell-type composition when applied over the XAV939 c_5_ condition in the hES cell line HES3 (Fig. 3c), depleting the non-neural ectoderm and neural crest-related fates in favor of neural identities like neuroectoderm, neuroepithelium and telencephalic progenitors (Fig. 3a, panel B1, versus Fig. 3b, panel B1 + F). While XAV939 treatments seemed more sensitive to FGF8 treatment in HES3 (Fig. 3c), RA treatments were most influenced by the addition of FGF8 in the iPS cell line WTC (Extended Data Fig. 6c).

We also compared all combinations of two morphogens to each of the corresponding single morphogen treatments and calculated MMD distances between them (Fig. 3d and Extended Data Fig. 6d). A small distance between a single morphogen treatment and its combination with another morphogen indicates a strong influence of that morphogen on the combination phenotype. In HES3, we observed similar MMD distances between all single morphogens and their pair-wise combinations (Fig. 3d), indicating an overall comparable contribution of both morphogens regardless of which morphogen was added first to the media. In WTC organoids, the timing of morphogen addition did not strongly affect the resulting cell-type composition either, but MMD distances were quite variable. The effects of SHH activation were stronger than WNT inhibition (XAV939), but weaker than WNT activation (CHIR), while RA was the dominating morphogen when combined with SHH modulation (Extended Data Fig. 6d). The different cell-type composition outcomes we observed across cell lines were not due to differences in underlying regulon activation under basal culture conditions (Extended Data Fig. 6h).

To unveil possible morphogen interactions, we then measured how much a certain morphogen combination would diverge from the expected additive effects of the two separated morphogens, using non-negative least square residuals as a metric of non-linearity.

In HES3, WNT inhibition and SHH activation also displayed interactive effects (Fig. 3e–g). XAV939 treatment (WNT inhibition) alone promoted diencephalic, neural crest and non-neuroectodermal fates in a dose-dependent manner, while SHH treatment alone induced progressive ventralization of anterior forebrain tissue, as previously described^40^. A combination of XAV939 and SHH showed synergistic effects leading to a higher proportion of non-neural ectodermal cells (Fig. 3f).

In WTC, we found that the balance between hindbrain progenitors and posterior floor plate is regulated by RA, FGF8 and SHH (Extended Data Fig. 6e–g). Organoids treated with RA alone generated a majority of posterior floor plate progenitors (53%) and a smaller proportion of hindbrain progenitors (19.5%). Higher SHH concentrations combined with RA increased the proportion of posterior floor plate progenitors (69% with SHH c_1_, 87.8% with SHH c_5_) and depleted hindbrain progenitors. Including FGF8 shifted the balance of regional fates back toward hindbrain progenitors (Extended Data Fig. 6f), a finding that is consistent with the role of FGF8 at the midbrain–hindbrain boundary^41^.

### hPS cell lines and neural induction method impact patterning outcome

**Extended Data Fig. 7:**
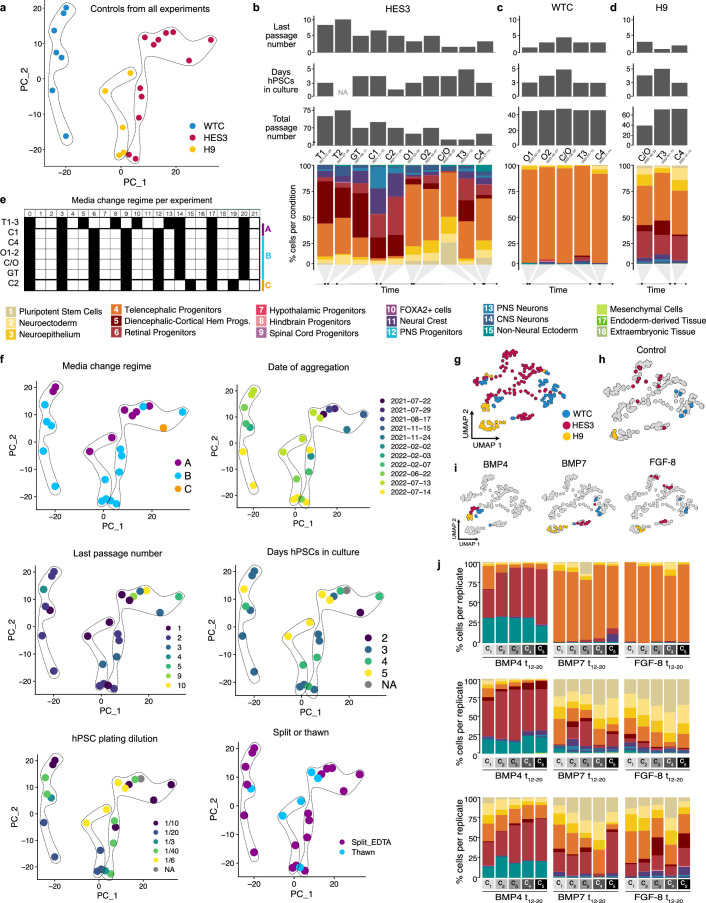
Comparison of patterning variability in the presence and absence of morphogen treatment. **a**, PCA projection of summarized control samples colored by cell line of origin. Each dot represents one pool of 4-16 organoids from the same line that were exposed to a certain morphogen treatment in each experiment/batch. **b-d**, Bar plots showing last passage number, days in culture, total passage number (top) and stacked barplot showing proportion of cell types (bottom) for each experiment for HES3 (**b**), WTC (c), H9 (**d**). The bottom line indicates the month when each experiment was conducted. T1-3, Timing experiments 1-3; C1-4, Concentration experiments 1-4; O1-2, Combination experiments 1-2; C/O, Concentration and combination (mixed) experiment; GT, Gradient Timing experiment (data not shown); **e**, Schematic indicating the medium change regime for each experiment. Black cells indicate a medium change event. **f**, PCA projection of summarized control samples colored by medium change regime (as indicated in **e**), date of aggregation, last passage number, days in culture, plating dilution, split or thawn hSC source. Each dot represents one pool of 4-16 organoids from the same line that were exposed to a certain morphogen treatment in each experiment/batch. **g**, UMAP projection of summarized experiments, with each sample colored by cell line of origin. **h** shows the same projection but only control samples are highlighted for each cell line. **i**, UMAP projection split by morphogen identity for BMP4, BMP7 and FGF8 concentration experiments, colored by cell line of origin. **j**, Cell type composition of neural organoids exposed to increasing concentration steps of BMP4, BMP7 and FGF8 (late exposure, from day 12 to 21). Top, WTC organoids, middle, HES3 organoids, bottom, H9 organoids. Cell type colour legend is identical to **b-d**.

hPS cell lines present distinct genetic and epigenetic properties that affect their differentiation potential and lead to biases in neural organoid regional development^4,42^. Multiple methods exist to induce neural development from multipotent embryoid bodies, including dual SMAD inhibition^43^ and MNIM^9^. Our patterning condition screen revealed variable organoid composition across hPS cell lines in the absence of morphogens (Extended Data Fig. 7a–f) and between neural induction methods, in this case only in the presence of patterning molecules (data not shown). We analyzed multiple culture variables and noted that, in MNIM without morphogens, the date of aggregation was the main factor correlating with changes in organoid composition (Extended Data Fig. 7b–f). When treated with increasing BMP4, BMP7 and FGF8 concentrations, each morphogen displayed variable consistency across hPS cell lines (Extended Data Fig. 7g–j).

**Fig. 4:**
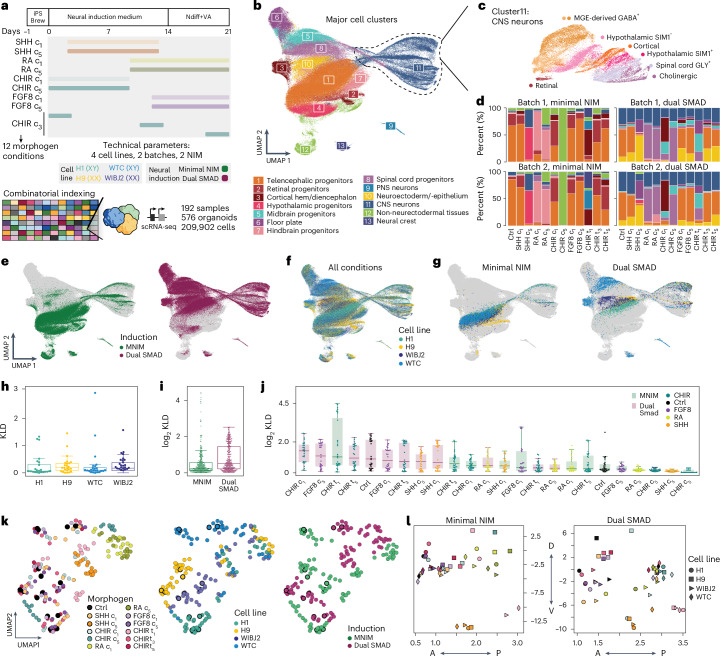
Morphogen patterning outcome is influenced by PS cell line and NIM. **a**, Experimental timeline showing morphogen treatment modalities in this study, an overview of the organoid culture protocol used and technical parameters that were tested. **b**, RSS-based UMAP projection of all cells in the dataset, including four cell lines (H1, H9, WIBJ2 and WTC), colored by cluster identity. **c**, RSS-based UMAP projection of subclustered CNS neurons, showing different regional identities. **d**, Stacked bar plots, showing the cell-type composition of each condition for H9-derived organoids, split by experimental batches. Concentrations are indicated by c_1_–c_5_. Cell-type color codes are identical to **b**. **e**, RSS-based UMAP projection of all cells in the dataset, colored by neural induction method. **f**, RSS-based UMAP projection of all cells in the dataset, colored by cell line. **g**, RSS-UMAP projection highlighting untreated (control) cells from each cell line of origin, split by neural induction method. Cells from morphogen treatment conditions are shown behind in gray. **h**, Box plots showing the distribution of KLD scores between experimental batches. Each dot represents a distance between experimental batches for each tested morphogen treatment and the indicated cell line. *n* = 24 for each category. KLD, Kullback–Leibler divergence. **i**, Box plots showing pair-wise distances between pairs of conditions derived from different hPS cell lines exposed to the same morphogen treatment and neural induction method. Each dot represents a distance between two cell lines when receiving the same morphogen treatment and undergoing the same neural induction method. *n* = 326 for each category. **j**, Box plots showing distribution of KLD scores between cell lines under the same treatment conditions. Each dot represents a distance between a pair of cell lines receiving the indicated morphogen treatment. *n* = 28 for each category. For the box plots in **h**–**j**, the center line indicates the median, the box limits show the lower and upper quartiles, and the whiskers extend to 1.5 times the interquartile range from the quartiles. **k**, UMAP projection of summarized experiments, with each sample colored by treatment condition (left), cell line of origin (center) and neural induction method (right). Each dot represents one pool of three organoids from the same line that were exposed to a certain morphogen treatment in each experiment/batch. Bold strokes around dots highlight the control samples. **l**, Scatterplot showing aggregated location of control and treatment conditions along the DV (vertical) and AP (horizontal) axes.

**Extended Data Fig. 8:**
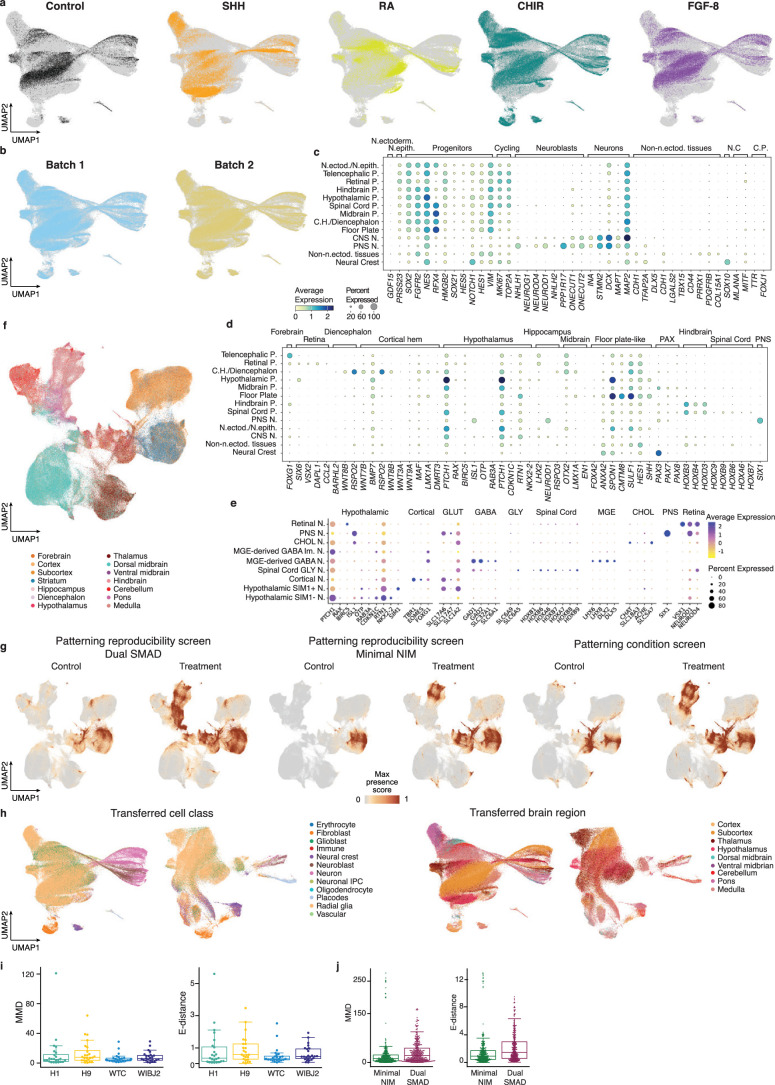
Annotation of regionalized patterning reproducibility screen single-cell dataset and projection to primary reference data. **a-b**, RSS-UMAP projection of data, colored by treated morphogen (**a**) or experimental batch (**b**). **c-d**, Dotplots showing cluster marker expression for the entire patterning reproducibility screen dataset, showing cell class markers **(c**) and brain region markers (**d**). N.ectoderm, Neuroectoderm; N.epith., Neuroepithelium; C.H.,Cortical Hem; PNS, peripheral neural system; CNS, Central Nervous System; N.C, neural crest; C.P., choroid plexus; P., progenitors; N. neurons; Non-n. ectod, non-neural ectoderm. **e**, Dotplots showing cluster marker expression for the CNS neurons subset. GLUT., glutamatergic; GABA, GABAergic; GLY, glycinergic; MGE, Medial ganglionic eminence; CHOL, cholinergic; PNS, peripheral neural system; N. neurons. **f**, UMAP of the human primary reference atlas radial glia subset28, colored by dissected regions. **g**, UMAP of the primary reference, colored by the max presence scores across all control and treatment conditions in patterning reproducibility and patterning condition screen. **h**, RSS-UMAP projection of patterning reproducibility screen and patterning condition screen, coloured by transferred cell class (left) and dissected brain region (right) of human primary reference atlas. **i**, Boxplots showing pair-wise MMD (left) and E-distance (right) between pairs of conditions derived from different hPSC lines exposed to the same morphogen treatment and neural induction method. Each dot represents a distance between two cell lines when receiving the same morphogen treatment and undergoing the same neural induction method. n = 24 for each category. **j**, Boxplots showing pair-wise distances between pairs of conditions derived from different hPSC lines exposed to the same morphogen treatment and neural induction method. Each dot represents a distance between two cell lines when receiving the same morphogen treatment and undergoing the same neural induction method. n = 326 for each category. MMD, maximum mean discrepancy; E-distance, energy distance. For panels **i–j**, boxplots are shown with the median indicated, where the boxes show the quartiles of the measurement and the whiskers, the 1.5 IQRs of the lower and upper quartile.

**Extended Data Fig. 9:**
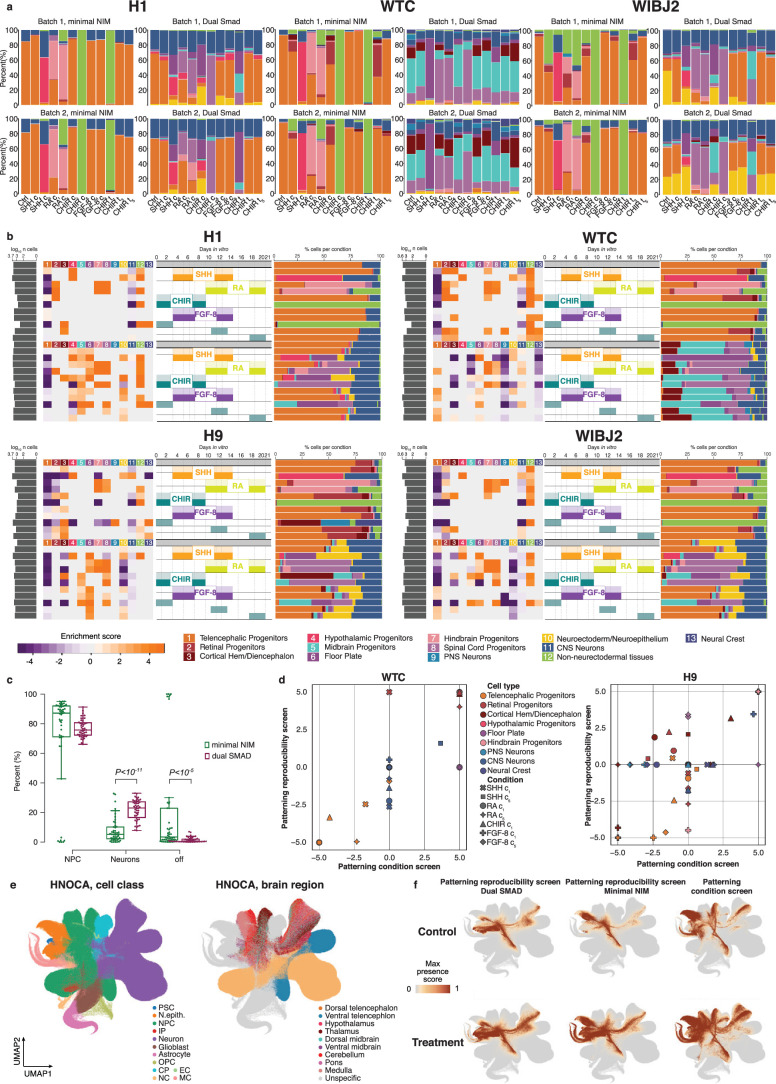
Comparison of patterning variability in the presence and absence of morphogen treatment. **a**, Stacked barplots, showing the cell type composition of each condition for H1 (left), WTC (middle) and WIBJ2 (right)-derived organoids, split by experimental batches. Concentrations are indicated by c1-5, treatment time windows are indicated by t1-t5. Cell types colour codes are identical to panel **b**. **b**, Barplots (left) show total number of cells sequenced and percentage of cells in each cluster per condition in the morphogen reproducibility experiments. Heatmap shows enrichment (orange) and depletion (purple) of each cluster across conditions (right). All results are presented split by cell line. **c**, Boxplots showing distribution of percentage of progenitors, neurons and off-target cell types, split by neural induction approach, with the median indicated, where the boxes show the quartiles of the measurement and the whiskers, the 1.5 IQRs of the lower and upper quartile. NPC, neural progenitor cells; off, off-target cell types. Statistically significant compositional differences were estimated using a linear model, implemented in the speckle package, followed by 2-sided t-test. P-values were FDR adjusted using Benjamini-Hochberg procedure. Exact adjusted P-values are 0.28 NPC, 9.4*10-11 for Neurons and 3.3*10-5 for off-target. n = 48 for each category, samples being each organoid. **d**, Scatterplot showing enrichment of cell types over control for patterning reproducibility (y-axis) and patterning condition screen (x-axis) for WTC (left) and H9 (right) cell lines. Shape of the dot indicates the morphogen treatment condition, color indicates thecell type. Datapoints aligning with the diagonal represent treatments that are highly reproducible in the two screening setups. **e**, UMAP of the integrated human neural organoid cell atlas (HNOCA), colored by cell class (left) and brain region (right) as provided in the original publication. PSC, pluripotent stem cell; N.epith, neuroepithelium; NPC, neural progenitor cell; IP, intermediate progenitor; OPC, oligodendrocyte progenitor cell; CP, choroid plexus; EC, endothelial cell; NC, neural crest; MC, mesenchyme. **f**, UMAP of the integrated human neural organoid cell atlas, colored by the max presence scores across all control and treatment conditions in patterning reproducibility and patterning condition screen.

To systematically assess the impact of these variables on patterning outcomes, we designed a patterning reproducibility screen (Fig. 4a–g and Extended Data Figs. 8 and 9) where 12 morphogen treatments were replicated using four hPS cell lines: H9 (hES cell, XX), H1 (hES cell, XY), WIBJ2 (hiPS cell, XX) and WTC (hiPS cell, XY). Each treatment was tested using two neural induction approaches and two technical batches (192 samples, 209,902 cells; Methods).

Data were integrated using RSS^27^-based comparison to an early developing human brain cell atlas^28^ and embedded with UMAP^29^ (Fig. 4b,c,e–g and Extended Data Fig. 8a,b). Cluster marker gene expression (Extended Data Fig. 8c–e and Supplementary Table 5) revealed cell identities corresponding to diverse CNS progenitors and neurons of the telencephalon, diencephalon, retina, hypothalamus, hindbrain and spinal cord domains, as well as midbrain progenitors and subpopulations of hypothalamic, cortical and spinal cord-derived neurons, which were predominantly generated under dual SMAD background conditions (Fig. 4b,c). We observed a higher ratio of neurons in dual SMAD conditions (Extended Data Fig. 9c), suggesting that dual SMAD inhibition promotes stronger neural fate acquisition and maturation. To validate cell-type annotation and assess which primary cell types or states were generated, we mapped organoid data to the single-cell transcriptomic atlas of the developing human brain^28^ (Extended Data Fig. 8f–h) and integrated human organoid atlas^24^ (Extended Data Fig. 9e,f). We analyzed normalized presence scores in both control and morphogen-treated conditions, revealing expansion of diverse reference cells generated after morphogen administration (including increased proportions of hypothalamus, midbrain, medulla, pons and cortex identities). We note that brain regions such as the cerebellum and the dorsal midbrain were still underrepresented in these conditions (Extended Data Fig. 8g,h), indicating more specialized protocols, using sequential and/or combinatorial patterning approaches are needed^44,45^.

We observed a remarkable degree of reproducibility between experimental batches for most conditions (Fig. 4d and Extended Data Fig. 9a), but the different Neural Induction Media (NIM) clearly generated different cell populations (Fig. 4e). Some cell types were predominantly produced by particular hPS cell lines (Fig. 4f). In the absence of morphogens, MNIM treatment led to more similar outcomes across lines in comparison to dual SMAD inhibition (Fig. 4g). We next performed a quantitative analysis of organoid reproducibility using three different distance metrics (Kullback–Leibler divergence, MMD and E-distance). While joint analysis of both induction methods revealed similar reproducibility scores across all cell lines (Fig. 4h and Extended Data Fig. 8i), we could confirm a tendency for increased variability across all conditions in organoids exposed to dual SMAD inhibition (Fig. 4i and Extended Data Fig. 8j). We noted a few outlier conditions in MNIM, corresponding to CHIR conditions that generated off-target cell states (Fig. 4i,j, Extended Data Fig. 9b and Supplementary Table 6). Overall, higher morphogen concentration conditions exhibited reduced variability across cell lines (Fig. 4j). When summarizing heterogeneity across condition, cell line and replicate experiments, we observed a pronounced segregation of morphogen conditions, neural induction methods and hPS cell lines (Fig. 4k). Analysis of variability across screens (by comparing common conditions in the common hPS cell lines H9 and WTC) confirmed overall correlation of organoid composition (Extended Data Fig. 9d).

To compare the effects of morphogens on DV and AP axes across cell lines, we calculated DV and AP scores for neural progenitors in each condition using a regularized linear model, trained on the radial glia of the human developing brain cell atlas^28^ (Methods). Subsequently, for each treatment condition and cell line, the average coordinates in AP–DV axes were calculated and visualized as a scatterplot (Fig. 4l). We observed that high concentration of SHH and RA produced similar effects across cell lines under both MNIM and dual SMAD inhibition conditions. In contrast, early CHIR timing consistently shifted all cell lines in ventral and posterior directions under dual SMAD inhibition, whereas cell lines showed inconsistent effects under MNIM. Conditions with weak patterning effects—including FGF8 at both concentrations and SHH and CHIR at low concentrations—failed to demonstrate consistent shifts in AP–DV axis coordinates across cell lines. We observed substantial differences in patterning outcome between MNIM and dual SMAD inhibition: Generally, dual SMAD inhibition conditions produced more dorsal neural progenitors than MNIM. Additionally, while MNIM control conditions consistently generated anterior neural progenitors, dual SMAD inhibition control conditions showed marked line-to-line variability: H9-derived progenitors were found dorsally and anteriorly, H1-derived and WIBJ2-derived progenitors were found ventrally and anteriorly, and WTC-derived progenitors were found posteriorly. This observation indicates that dual SMAD inhibition promotes stringent neural fate acquisition but makes the resulting patterning outcome more susceptible to hPS cell line variability than MNIM.

### Assessing reproducibility across cell lines and NIM at the regulon level

**Fig. 5:**
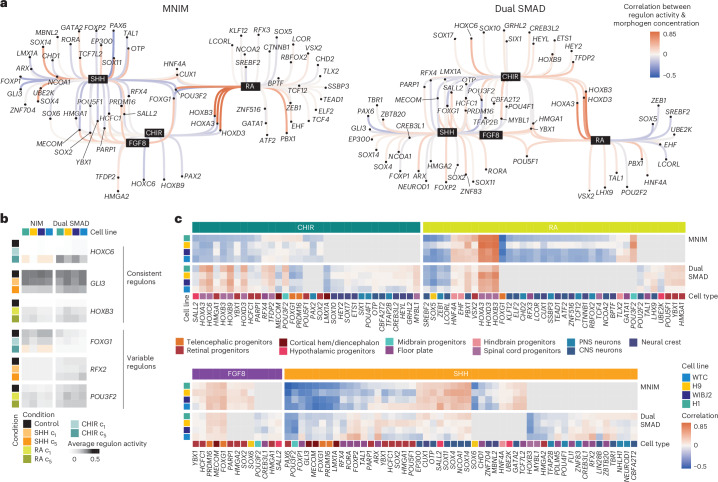
Variable and consistent regulons across hPS cell lines and NIM. **a**, Inferred regulon network for tested patterning molecules for MNIM (left) and dual SMAD inhibition (right) neural induction approaches. Each black node represents a morphogen that is connected to a number of associated regulons (showing correlating activity to morphogen treatment). Color of edges represents correlation of regulon activity with morphogen concentration for the H9 cell line. **b**, Examples of regulon activity in each cell line and morphogen condition for regulons, consistent and inconsistent across cell lines. Beige wells indicate that regulon activity was not inferred for the stated condition. This is because some conditions are represented mostly by non-neural ectoderm-derived tissues, which were excluded for regulon analysis. **c**, Heat map representing the correlation of regulon activity with morphogen concentrations for each cell line for regulons in MNIM and dual SMAD inhibition media. The color panel on the bottom refers to the cell type where that regulon registers maximum activity (excluding non-neural and undifferentiated clusters). Gray wells indicate that regulon was not inferred for the stated neural induction approach.

Our early sampling time point (21 days) allowed us to study the activation of individual regulons responsible for early nervous system regionalization at the single-cell level. We first inferred a global regulatory network for each neural induction method (Methods). We then examined the dependency of regulon activity on morphogen concentration across cell lines separately for MNIM and dual SMAD inhibition-based neural induction methods (Fig. 5a). For each morphogen–transcription factor pair, we assessed the consistency of the correlation between regulon activity and morphogen concentration across hPS cell lines (Methods). Among the regulons that exhibited consistent activity across cell lines were well-known patterning regulators such as *HOXB3*, a cervical *HOX* gene activated by RA, and *HOXC6*, a thoracic *HOX* gene activated by CHIR. Notably, the activity of the *GLI3* regulon was consistently reduced across all cell lines in response to SHH, consistent with previous studies about the role of *GLI3* in DV human brain patterning^46^. Interestingly, *FOXG1*, *RFX2* and *POU3F2* were among the most variable regulons in response to CHIR, SHH and RA, respectively, both across hPS cell lines and neural induction methods (Fig. 5b).

Overall, morphogen effects on different cell lines always followed the same directionality within the same neural induction method. However, we did observe some regulons with opposite responses in MNIM versus dual SMAD inhibition. This was most obvious under CHIR treatment, where HOX regulons were activated in dual SMAD conditions but downregulated in MNIM (Fig. 5c).

RA and FGF8 showed the highest consistency across neural induction methods. FGF8-associated regulons, mostly related to retinal and telencephalic development, showed mild dependency on FGF8 concentration across lines and induction methods. Under RA treatment, HOX regulons were the most consistently activated across induction methods and hPS cell lines. We observed a set of regulons that was consistently downregulated by RA in MNIM conditions but remained largely unaffected in dual SMAD inhibition media. These regulons were mostly associated with neuronal and neural crest fates. A set of retinal development regulons (*POU5F1*, *YBX1*, *HMGA1*) could only be modulated by RA in dual SMAD conditions. Notably, *FOXG1* and *POU3F2* displayed divergent behaviors in response to RA in the two different media. RA treatment induced consistent effects across hPS cell lines; only H9 in MNIM showed many divergent regulons (Fig. 5c).

In SHH treatments, *PAX6* and *GLI3* downregulation was consistent across hPS cell lines and neural induction methods. However, some regulons showed drastic differences in behavior. For example, *HOXB3* was strongly downregulated in dual SMAD conditions but not affected in MNIM. Conversely, *SOX6* was only downregulated in MNIM conditions. Overall, many key patterning regulons associated with SHH treatments (*OTP*, *ARX*, *FOXP2* or *TCF7L2*) showed widely variable responses across lines and neural induction methods (Fig. 5c).

When comparing hiPS and hES cell-derived organoids, we did not detect any notable difference, as well as when comparing across karyotypes (XX versus XY).

The collective evidence presented here demonstrates that the regulon activity changes in response to patterning molecules have consistent directionality across cell lines, but are highly dependent on the neural induction approach.

### Morphogen gradation influences DV forebrain proportion

**Fig. 6:**
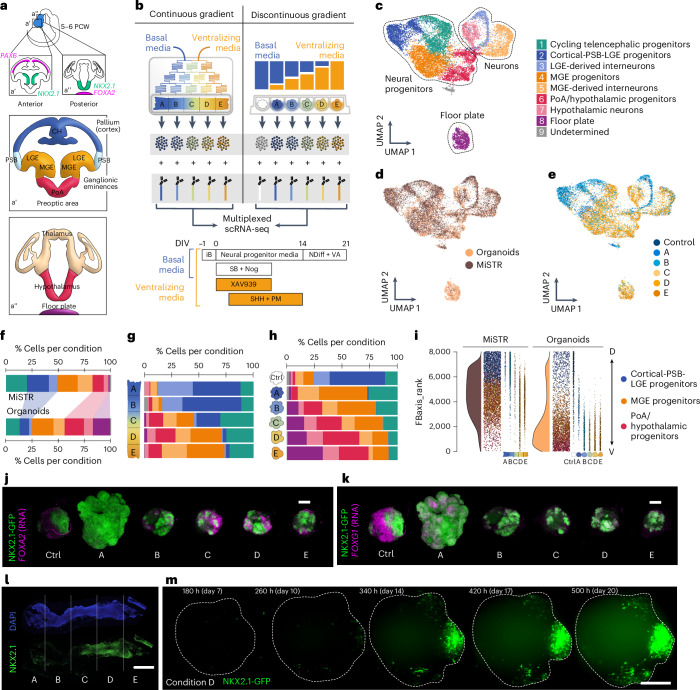
Morphogen gradients and discontinuous morphogen steps similarly recapitulate DV patterning of the human forebrain. **a**, Overview of DV patterning of the developing forebrain and the corresponding regions and marker genes expressed in each of them. Different AP sections of a 5–6-week-old human embryo and their patterning profiles are depicted in a’ (telencephalic, *FOXG1*+, anterior) and a’’ (hypothalamic, *NKX2.1*+, posterior). PCW, post-conception week. **b**, Simplified schematic of the microfluidic gradient tree (MiSTR) and the 96-well setup (organoids) used for treating developing neural tissue with increasing concentrations of SHH. Bottom, culture protocol for both experimental setups. iB, iPS Brew; SB, SB431542; Nog, Noggin; DIV, days in vitro. **c**–**e**, UMAP projection of Harmony-integrated scRNA-seq data from MiSTR and organoid experiments (both using the hES cell line H9), colored by cluster (**c**), experimental setup (**d**) and section of origin (**e**). PSB, pallial–subpallial boundary; LGE, lateral ganglionic eminence; MGE, medial ganglionic eminence; PoA, preoptic area. **f**, Cell-type composition of all aggregated segments from each experimental setup. **g**,**h**, Cell-type composition of each segment from MiSTR tissues (**g**) and separately patterned organoids (**h**). **i**, Forebrain axis score ranking of cells from each experimental setup, representing shifts from anterodorsal identities (higher ranks) toward posterior–ventral identities (lower ranks). For each of them: left, ridge plot showing distribution of cells along FBaxis_rank values; middle, jitter plot showing the distribution of each progenitor type along the FBaxis ranking (most dorsal cells at the top, most ventral cells at the bottom); right, same jitter plot showing separately each MiSTR segment and organoid condition. **j**,**k**, HCR stainings of organoids patterned with increasing concentrations of SHH, purmorphamine (PM) and XAV939. Endogenous NKX2.1-GFP protein is shown in green, while *FOXA2* (**j**) and *FOXG1* (**k**) mRNA levels are shown in magenta. Images are representative of four organoids from each condition cultured simultaneously. **l**, Example immunohistochemistry staining of MiSTR neural tissue exposed to a continuous morphogen gradient. Orientation of the tissue is shown with **a**–**e**, indicating each subsection. Scale bar, 250 µm. Four MiSTR tissues from similar culture conditions (slight variations in SHH and PM concentrations) were stained and showed similar compartmentalization of NKX2-1 expression. **m**, Still images from long-term light-sheet imaging of organoids containing the NKX2.1:GFP reporter showing focal induction and spreading of ventral domains. Images are representative of five organoids cultured simultaneously using the concentration of SHH, PM and XAV9393 corresponding to condition D.

**Extended Data Fig. 10:**
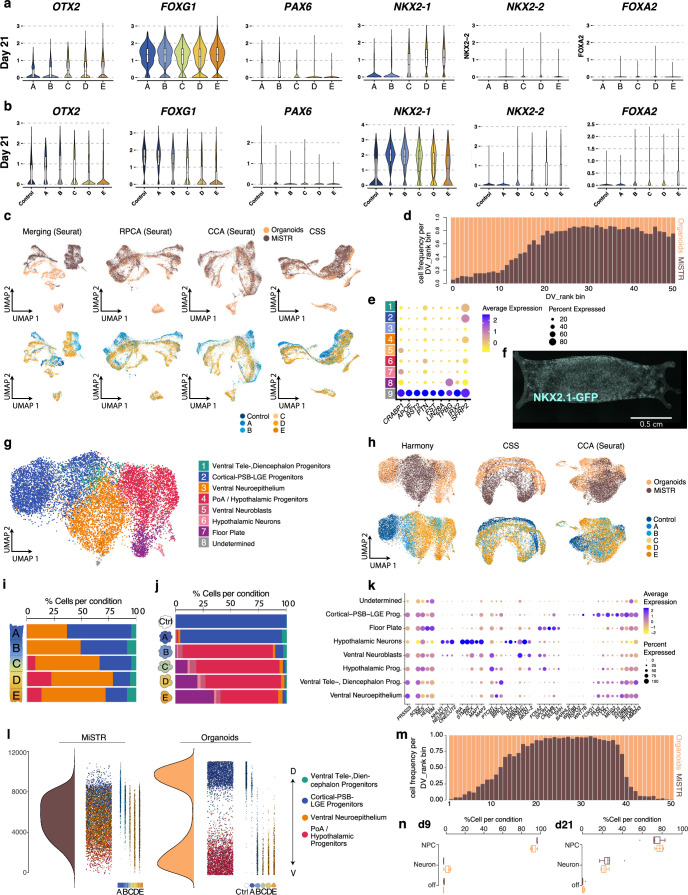
Characterization and data integration of MiSTR and MiSTR-like organoid cultures. All experiments were performed with the hESC line H9. **a, b**, Expression patterns of key forebrain patterning genes across sections A-E in MiSTR tissue (**a**) and MiSTR-like organoids (**b**), showing a tendency towards higher ventralization in organoids, sampled at day 21. For panel a, n = 368 for A, n = 1902 for B, n = 404 for C, n = 2152 for D, n = 1562 for E. For panel b, n = 967 for Control, n = 483 for A, n = 955 for B, n = 669 for C, n = 1144 for D, n = 997 for E. For panels **a-b**, boxplots are shown with the median indicated, where boxes show the quartiles of the measurement and whiskers show the 1.5 IQRs of the lower and upper quartile. **c**, UMAP resulting from different methods showing the robustness of the integration, colored by sample source (top) and segment (bottom), sampled at day 21. **d**, Distribution of MiSTR (brown) and MiSTR-like organoid cells (orange) across the binned DV ranks, showing coverage of the same dorso-ventral domains at different frequencies, sampled at day 21. **e**, Dotplots showing cluster marker expression for the dataset at day 21. Annotations are kept consistent with Fig. 6c: 1-Cycling Telencephalic Progenitors, 2-Cortical-PSB-LGE Progenitors, 3-LGE-derived Interneurons, 4-MGE Progenitors, 5-MGE-derived Interneurons, 6-PoA / Hypothalamic Progenitors, 7-Hypothalamic Neurons, 8-Floor Plate, 9-Unknown. PSB, Pallial-Subpallial Boundary; LGE, Lateral Ganglionic Eminence; MGE, Medial Ganglionic Eminence; PoA, Preoptic Area. **f**, Fluorescence image showing NKX2.1-GFP expression in control MiSTR tissue cultured in the absence of PDMS microfluidic tree. This culture was performed under patterning conditions for segment D, and the image (representative of 3 cultures under D conditions) was taken on differentiation day 12. Induction patterns were similar across other 2D MiSTR cultures under different morphogen conditions. **g**, UMAP projection of Harmony-integrated single-cell RNA-seq data from MiSTR and organoid experiments, day 9, colored by cell type. **h**, UMAP resulting from different methods showing the robustness of the integration, colored by sample source (top) and segment (bottom). **i,j**, Cell type composition of each segment from MiSTR tissues (**not h, but i**) and separately patterned organoids (not **i**, but j), sampled at day 9. **k**, Dotplots showing cluster marker expression for the dataset at day 9. PSB, Pallial-Subpallial Boundary; LGE, Lateral Ganglionic Eminence; Prog., progenitors. **l**, Forebrain axis score ranking of cells from each experimental setup, representing shifts from anterodorsal identities (higher ranks) towards posterior-ventral identities (lower ranks). For each of them: left, ridge plot showing distribution of cells along FBaxis_rank values; middle, jitter plot showing the distribution of each progenitor type along the FBaxis ranking (most dorsal cells at the top, most ventral cells at the bottom); right, same jitter plot showing separately each MiSTR segment and organoid condition, sampled at day 9. **m**, Distribution of MiSTR (brown) and MiSTR-like organoid cells (orange) across the binned DV ranks, showing coverage of the same dorso-ventral domains at different frequencies, sampled at day 9. **n**, Boxplots showing distribution of percentage of progenitors, neurons and off-target cell types, split by tissue source at day 9 (left) and day 21 (right), with the median indicated, where the boxes show the quartiles of the measurement and the whiskers, the 1.5 IQRs of the lower and upper quartile. n = 5 for MiSTR and n = 6 for organoids category for each timepoint and cell class. NPC, neural progenitor cells; off, off-target cell types.

Morphogen gradients form boundaries and organizing centers, which in turn influence region proportion during brain development^3^. We used two approaches to test if graded change in morphogen exposure can control the proportion of dorsal and ventral telencephalon cells within complex human neural tissues (Fig. 6a,b and Extended Data Fig. 10). In the first approach, we use a two-input microfluidic tree gradient generator, which has previously been used for AP neural tube specification (Microfluidic STem cell Regionalization or MiSTR)^47^, to introduce a DV gradient. Here, a three-dimensional sheet of developing neural tissue received a gradient of SHH agonist and WNT antagonist molecules (Fig. 6b). In a second approach, we exposed neural organoids to discrete concentrations of the same agonists representing different locations of the gradient, including a control condition not exposed to patterning molecules (Fig. 6b and Supplementary Table 7). Both culture settings used dual SMAD inhibition for neural induction. After 21 days in culture, we collected MiSTR tissue and neural organoids to perform scRNA-seq, using cell hashing to track the MiSTR segment and organoid condition (Fig. 6b). In both datasets, we observed marked differences in the expression of DV patterning genes across segments (Extended Data Fig. 10a,b and Supplementary Table 8). Datasets were merged and integrated using Harmony, RPCA, CCA and CSS, with all integrations yielding a strong overlap between the two approaches (Extended Data Fig. 10c). We note an exceptional *FOXA2*+ floor plate cluster, which was enriched in organoids treated with high doses of SHH (Fig. 6c–e and Extended Data Fig. 10c).

Similar cluster compositions were observed using both approaches, although with different proportions, and organoids contained more ventral regions than the tissue patterned with a microfluidic gradient (Fig. 6f). Low SHH regions in the MiSTR tissue produced cortical/lateral ganglionic eminence (Ctx/LGE) progenitors and neurons, whereas the lowest concentration of SHH in the organoids induced more ventral fates, including MGE progenitors and MGE-derived interneurons (*FOXG1*+, *NKX2-1*+). While increasing SHH concentrations induced mainly MGE fates in the MiSTR, high SHH induced a combination of hypothalamic fates (*FOXG1*−, *NKX2-1*+) and diencephalic floor plate fates (*FOXA2*+) in the organoids (Fig. 6g,h).

Forebrain axial scoring and ranking (FBaxis_rank) revealed a continuity of the axial domains generated in each of the settings, with MiSTR tissue cells dominated by telencephalic DV fates and organoid cells dominated by diencephalic ventral fates (Fig. 6i and Extended Data Fig. 10d). Immunostaining and HCR in situ hybridization confirmed these findings: while organoids displayed *NKX2-1* expression in all conditions, including control (Fig. 6j,k), MiSTR tissue showed restricted *NKX2-1* expression in the segments with higher SHH exposure (Fig. 6l; sections C–E).

We speculated that these differences could be due to differences in morphogen availability in a flowing microfluidic polydimethylsiloxane (PDMS) chamber versus a plate setting with intermittent media change. To test this hypothesis and find out whether ventralization was generally stronger in organoids, we sampled MiSTR tissue and neural organoids on day 9 of the protocol and integrated the data again using multiple methods (Extended Data Fig. 10g,h). This earlier time point showed the emergence of equivalent regional cell types (Extended Data Fig. 10g). However, while MiSTR segment identities ranged from cortical-PSB-LGE progenitors to hypothalamic progenitors, organoids receiving intermediate amounts of SHH directly jumped to hypothalamic-like signatures (Extended Data Fig. 10i–k). When we scored the positioning of each system in the DV forebrain axis, we confirmed that the MiSTR setup produced much more medial identities with gradual step changes, whereas organoids displayed drastically dorsal or ventral identities depending on whether they surpassed specific SHH concentrations (Extended Data Fig. 10l–n). This rules out the possibility that SHH concentrations are generally lower in the MiSTR setup due to PDMS absorbance.

To investigate these differences in patterning dynamics between the two systems, we performed live fluorescence imaging of NKX2-1:GFP organoids and MiSTR tissue growing in high SHH conditions. Interestingly, NKX2-1-driven fluorescence^48^ emerged at very localized areas in organoids under ‘D’ conditions, despite nonlocalized exposure to SHH; and these foci gradually spread (Fig. 6m). Meanwhile, rectangular monolayer cultures resembling segment D of the MiSTR setup showed homogeneous, low expression levels of NKX2.1-GFP on day 5 that progressively increased in a simultaneous manner across the tissue (Extended Data Fig. 10f). Altogether, these data suggest a feed-forward mechanism that reinforces neural organoid response to SHH signaling, possibly facilitated by a higher number of neighboring cells in comparison to pseudo-monolayer (2.5D) cultures.

## Discussion

We provide a systematic evaluation of timing-dependent and concentration-dependent effects of morphogen signaling on developing human neural organoids. Our screen delimits competence windows for SHH, FGF8, RA, BMP4/BMP7 and WNT pathway modulators, demonstrating that cell fate decisions can be controlled through morphogen timing and dosage. For example, SHH and RA enriched different regional fates at low-versus-high concentrations compared to untreated organoids. We identified new morphogen synergies beyond those known to cooperatively pattern the neural tube. RA stimulated posterior floor plate formation with SHH^49–53^ or hindbrain progenitors with FGF8, consistent with established roles in hindbrain specification^2,3^. Further, we found that WNT inhibition (XAV939) combined with SHH activation increases non-neural ectoderm, an effect also seen for early activation of WNT (CHIR). Interestingly, this effect was observable only without dual SMAD inhibition, highlighting how neural induction methods influence morphogen responses.

Our systematic analysis addresses hPS cell line and protocol variability at the cell-type and regulon level. Unlike previous studies that sample later developmental time points and focus on neuronal populations^54–56^, we harvested organoids at an early time point (day 21) to capture neuroepithelial populations through patterned progenitors. This approach enabled measurement of patterning regulon activity during regionalization events, providing a unique developmental snapshot complementing existing resources. Our data also open up numerous possibilities for computational modeling and prediction of patterning outcomes^57^. While we found no generalizable rules for hPS cell line variability, our data identify specific regulons and cell types requiring optimization for reproducible patterning. The inconsistency likely reflects genetic or epigenetic differences affecting baseline expression and signaling pathway susceptibility^42,58–60^. Neural induction methods substantially impact regionalization outcomes: dual SMAD inhibition produces predominantly CNS fates with higher variability, while MNIM generates diverse neuroectodermal and non-neuroectodermal tissues with greater reproducibility. Our systematic analysis of batch-to-batch variability yielded consistent results with experimental batches separated by only 2 weeks. This aligns with findings from our patterning condition screen, where longer time intervals between experiments correlated with increased variability. We hypothesize that reagent degradation and changing hPS cell states contribute to this time-dependent variability.

Our MiSTR-versus-organoid comparison highlights the importance of tissue architecture in morphogen responses. The MiSTR model’s elongated neural tube-like structure produces milder ventralization likely through lateral inhibitory mechanisms, while organoids enable study of self-organizing interactions where locally secreted morphogens may amplify patterning signals. Both systems generated composite tissues with multiple regional identities corresponding to spatially adjacent brain regions, suggesting intrinsic self-organizing responses create relevant boundaries even under uniform conditions. Higher morphogen concentrations increased organoid homogeneity, although regions responding to lower concentrations remain difficult to reproduce. The prevalence of multiregional organoids indicates that gradient formation likely establishes regional domains, as confirmed by our microfluidic gradient experiments showing compositional shifts along concentration axes.

Several important limitations should be considered when interpreting these findings. Line-to-line differences and batch variation contribute substantially to organoid outcome variability, highlighting the need for standardized experimental documentation to enable proper cross-study comparisons. Our use of multiple molecules targeting individual pathways (for example, SHH/purmorphamine, BMP4/BMP7) may confound pathway-specific interpretations but provides valuable information for assessing relative molecule potency and protocol optimization. Organoid seeding cell number and size at treatment initiation impact patterning by influencing morphogen accessibility, cell density and signaling gradient establishment, variables that should be systematically tested in future studies. Our analysis focuses on cell composition and gene expression rather than morphology and spatial arrangement. However, the location of a cell within a three-dimensional structure may impact its patterning outcome due to concentration gradients and mechanical constraints. This spatial factor possibly contributed to the regional heterogeneity observed within organoids treated with the same patterning molecules. Finally, media change timing may disrupt endogenous patterning gradients, and our day-21 time point, although representing a compromise for capturing patterning events with robust cell identification, may miss critical earlier specification or later refinement processes that longitudinal analysis could reveal.

Despite these limitations, this work, together with recent work on later stages of neural organoids^45,55^, provides an integrated resource for morphogen-induced neural regionalization in vitro, demonstrating that systematic screens with single-cell readouts offer powerful approaches for protocol optimization and understanding the effect of morphogen pathway modulations on brain organoid patterning. Future studies incorporating later time points, additional signaling pathways and more sophisticated culture systems and readout modalities (for example, epigenomics) will further advance our ability to model human brain development in vitro.

## Methods

### Ethics statement

Stem cell experiments with WTC, WIBJ2 hiPS cells, H9, H1 and HES3 (NKX2.1GFP/w) hES cells were approved by the Bundesamt für Gesundheit (Swiss Health Federal Office) with disposition number 606.0000-1/31/19.018224; and by the Ethikkommission Nordwest- und Zentralschweiz (Ethics Commission for Northern and Central Switzerland).

### iPS cell and ES cell culture

NKX2.1GFP/w (HES3) hES cells were obtained from A.K.’s research group at the University of Copenhagen, after the arrangement of a material transfer agreement with E. Stanley and A. G. Elefanty (Murdoch Children’s Research Institute, Melbourne). H1 and H9 (WA09) hES cells were obtained from WiCell; WTC hiPS cells from the Allen Institute for Cell Science; and WIBJ2 cells from the HipSci resource. Stem cells were grown at 37 °C and 5% CO_2_ in feeder-free conditions, on six-well tissue culture plates coated with hES cell-Qualified Matrigel (Corning). They were fed every other day with mTesR Plus (StemCell Technologies) or StemMACS iPS Brew XF (Miltenyi) and passaged when reaching around 80% confluence with EDTA for gentle dissociation. All lines were tested regularly for copy number changes using the Agilent ISCA 8x60K v2 array and no abnormalities were detected. Additionally, a karyotyping control was performed and showed normal XX (NKX2.1GFP/w and H9) and XY (WTC) karyotypes. These analyses were performed by the Cell Guidance Systems Genetics Service. Regular PCR-based mycoplasma testing (Biological Industries) was performed to discard potential *Mycoplasma* infections.

### Neural organoid generation and incubation with patterning molecules

Stem cells were washed with PBS once and trypsinized using TrypLE Express Enzyme (Thermo Fisher Scientific) to obtain single-cell suspensions and 500 cells were plated in each well of ultralow-attachment 96-well plates (day −1). A 1–5-min centrifugation of the well plate was performed at 200*g* and cells in suspension were cultured for one day in mTesR Plus (StemCell Technologies) to allow for aggregation and embryoid body formation. On day 0, embryoid bodies were transitioned to MNIM (containing DMEM/F12, 1% N2 supplement (vol/vol), 1% GlutaMAX supplement (vol/vol), 1% MEM-NEAA (vol/vol) and 1 μg ml^−1^ heparin). MNIM was maintained for 14 days and then switched to NDiff+VA (neural differentiation media with vitamin A), with the exception of the RA/XAV939/SHH/FGF8 timing experiments, which used NDiff−VA instead until day 21. The specific morphogen treatment windows and concentrations used per condition are specified in Supplementary Fig. 1 and are designed based on known published protocols (Supplementary Table 9).

In the MNIM versus dual SMAD inhibition experiments, control dual SMAD organoids were cultured in neural patterning media (see MiSTR and MiSTR-like organoid culture sections below) and exposed to 10 μM SB (SB432542, Miltenyi) and 100 ng ml^−1^ Noggin (rh-Noggin, Miltenyi) from day 0 to 9. MNIM organoids were cultured in the NIM described above. For organoid patterning, we used 4.5 μM XAV939 (Miltenyi) from day 0 to 9 and 180 ng ml^−1^ SHH (rh-SHH/C24II, Miltenyi) together with 270 nM purmorphamine (Miltenyi) from day 3 to 14.

### MiSTR cultures

MiSTR culture was performed as previously published^47^ with some modifications to patterning in the DV axis. One day before the assembly of the MiSTR device and start of differentiation (day −1), H9 cells were detached from the well using EDTA to obtain single-cell suspensions, which were adjusted to a concentration of 1.25 × 10^6^ cells per ml in iPS Brew + 10 μM ROCK inhibitor (Y-27632, VWR). Then, 800 μl of cell suspension (approximately 1 million cells) were plated on top of a pure GFR-Matrigel (Corning) bed polymerized within a PDMS chamber in the bottom part of the MiSTR device. Cells were incubated for a day (37 °C, 5% CO_2_) to allow them to proliferate and form a uniform sheet. On the next day, dead cells and debris were removed from the chamber by washing twice with a 1:20 dilution of KOSR (Knock-Out Serum Replacement, LifeTech) in DMEM/F12 (LifeTech). After aspirating bubbles and pre-perfusing the microfluidic tree with the same solution, the top part of the MiSTR device (containing the PDMS microfluidic tree) was fit over the bed of ES cells. Lastly, the polycarbonate lid was placed on top and the whole setup was pushed into the POM cassette for stabilization.

Once the ‘sandwich’ was assembled, two high-precision GasTight syringes (Hamilton) with different media were attached to the tubing coming out of either side of the MiSTR device:

- The left-side syringe contained default neural patterning media: 50% DMEM/F12 (LifeTech), 50% NeuroMedium (Miltenyi), a 1:200 dilution of Glutamax (LifeTech), a 1:250 dilution of penicillin–streptomycin (LifeTech), NB-21 (NeuroBrew-21 without vitamin A, Miltenyi) and a 1:200 dilution of N2 supplement (LifeTech). These media were complemented with 10 µM SB (SB432542, Miltenyi) and 100 ng ml^−1^ Noggin (rh-Noggin, Miltenyi) for neural induction from day 0 to day 9.
- The right-side syringe contained the same neural patterning media as above, also complemented with SB and Noggin for neural induction on days 0–9, and the following patterning molecules: from day 0 to 9, 5 μM of XAV939 (Miltenyi); from day 3 to 14, 200 ng ml^−1^ of SHH (rh-SHH/C24II, Miltenyi) and 0.3 μM of purmorphamine (Miltenyi).

After bringing the MiSTR device into the incubator and assembling the syringes on high-precision neMESYS pumps, the flow was initiated and kept at a constant rate of 160 μl h^−1^. Meanwhile, the outlet tubing would collect waste media into a small glass bottle placed on an upper shelf of the same incubator. The syringe media were changed or replenished every 2 or 3 days and the differentiation was maintained in the microfluidic tree for 14 days. Then the MiSTR device was disassembled, and the neural sheet was recovered and completely embedded in GFR-Matrigel by adding 200 μl on top of it. After polymerization of the fresh Matrigel, the tissue was transported into a Petri dish with Ndiff+VA media (see composition above, under ‘Neural organoid generation and incubation with patterning molecules’). Cultures were kept in the incubator under mild rocking conditions to ensure good perfusion and media were changed every 3–4 days until day 21. On day 21 of MiSTR culture, the neural sheet was retrieved and cut in five equal parts from left to right, following the longitudinal axis of the gradient in the chamber. A custom-made mold was used to standardize the sizes of each piece. From left to right (basic neural patterning media on the left to neural patterning media with patterning factors on the right), the sections were called A, B, C, D and E. Each section from A to E is represented by two independent MiSTR experiments, which were pooled for dissociation. Before and after each experiment, the different parts of the MiSTR device were sterilized appropriately by either ethanol baths or autoclaving, according to their material.

### MiSTR-like organoid cultures

On day −1, embryoid body aggregation of H9 and HES3 (NKX2.1GFP/w) hES cells was performed as described in the previous section, using iPS Brew and ROCK inhibitor at a 1:200 dilution. For neural induction, neural patterning media were used together with SB and Noggin-mediated dual SMAD inhibition from day 0 to day 14. After this, the media were changed to neural differentiation media with vitamin A (composition previously described) until day 21. Overall, organoids were grown in identical media conditions as the MiSTR cultures, but in 96-well plates for the entire time course. Each of the organoid conditions was treated with the average morphogen concentration to which the corresponding MiSTR segment was exposed (Supplementary Table 7).

### Live imaging of developing MiSTR-like organoids and two-dimensional neural sheets

For the light-sheet imaging experiments, MiSTR-like organoids from conditions ‘control’ (*n* = 4) and ‘D’ (*n* = 4) were aggregated on day −1 and transferred to an imaging cuvette one day later (day 0 of the culture protocol). Matrigel diluted to a 1:50 ratio was used to fix the organoids in their position in the microchambers within the cuvette. The LS1 live light-sheet microscope (Viventis) was used to image each organoid every hour with a ×25 objective demagnified to ×18.5, across 200 optical slices separated by a 2-μm step size. In total, MiSTR-like organoids were imaged for 21 days^48^.

For the imaging of two-dimensional cultures of MiSTR-like neural progenitors, a custom-made chamber was made with the same dimensions as the MiSTR chamber. This chamber was used to contain 1 million H9 and HES3 (NKX2.1GFP/w) hES cells, which were seeded on day −1 on a bed of pure Matrigel. From day 0 until day 14, they were exposed to neural patterning media and the morphogen cocktail corresponding to ‘control’ and condition ‘D’ (as described in the previous section). Imaging of the resulting neural sheets was performed using a Nikon Ti2 microscope with a spinning disk module. Tile scans of the entire tissue were taken every day until NKX2.1-GFP activation, at a ×4 magnification in three different *z*-step locations. Media were exchanged every day, and the cultures were maintained until day 21.

### In situ HCR

Tissue collection and fixation: on day 21 of culture, neural organoids were collected from the 96-well plates and pooled by condition in 2-ml microcentrifuge tubes. After removing leftover media, 2 ml of cold 4% paraformaldehyde (PFA; Thermo Scientific, 28908) in RNase-free PBS (Invitrogen, AM9625) was added and samples were fixed for 2 h or overnight at 4 °C. All subsequent incubation steps were done with mild rocking using a nutator at 4 °C unless otherwise stated to preserve the quality of endogenous mRNAs. After fixation, PFA was removed and samples were washed three times with PBST (RNase-free PBS and Tween20, Sigma-Aldrich) with incubation times of 15 min. After the last wash, half of the PBST was removed, and the same amount of methanol (MeOH) was added to make it a 50:50 PBST–MeOH mixture (vol/vol). Samples were incubated for 10–15 min and the PBST–MeOH solution was exchanged for 100% MeOH, then incubated for 15 min and replaced by new, pure MeOH for overnight storage at −20 °C.

On the day of the experiment, samples were rehydrated with a series of graded PBST/MeOH washes (25% PBST/75% MeOH, 50% PBST/50% MeOH, 75% PBST/25% MeOH, 100% PBST twice) for 5 min each at 4 °C. Then PBST was removed, and samples were treated with 0.5 ml of 10 μg ml^−1^ proteinase K (AM2546, Thermo Fisher) in PBST for 3 min at room temperature. This concentration and time were previously optimized for good permeabilization without disruption of neural organoid tissue. Next, organoids were washed twice for 2 min with 1.5 ml of PBST, then postfixed with 0.5 ml of 4% PFA (20 min at room temperature) and washed again three times for 5 min with 1.5 ml of PBST.

Detection stage: Before hybridization, organoids were treated with 200 μl of probe hybridization buffer for 30 min at 37 °C inside a Thermomixer covered by the ThermoTop (Eppendorf) to avoid evaporation of the buffer. The probe hybridization buffer was then removed and 200 μl of probe solution was added to the organoids, which were incubated overnight at 37 °C as previously mentioned. The probe solution contained 100 μl of probe hybridization buffer and 0.5 μl of each hybridization probe (targeting each gene, 1 μM stock), up to a total of five probes. On the next day, excess probes were removed by washing four times for 15 min with preheated probe wash buffer (30% formamide, 5× SSC, 9 mM citric acid pH 6, 0.1% Tween20, 50 μg ml^−1^ heparin, dilution in ultrapure water) at 37 °C. Finally, organoids were washed twice for 5 min with 2 ml of SSCT (0.1% Tween20 in 5× SSC buffer, Sigma) at room temperature.

Amplification stage: organoids were incubated with 1 ml of amplification buffer for 10 min at room temperature. Meanwhile, hairpin mixtures were prepared with 100 μl of amplification buffer and 2 μl of each pair of snap-cooled hairpin probes (amplification probes, 3 μM stock). Each hybridization probe pair had two corresponding amplification probes that would bind two hybridization probe pairs. Due to their cross-reactivity (the chain reaction could be prematurely triggered if they were in contact), these hairpin amplification probes were snap-cooled separately by heating them to 95 °C for 90 s and cooling them down to room temperature in a dark drawer for 30 min. Once the pre-amplification time was over, the solution was removed and 100 μl of the hairpin mixture was added to the organoids, which were then incubated overnight (12–16 h) in the dark at room temperature. On the next day, excess hairpins were removed with several washing steps using 2 ml of 5× SSCT at room temperature: first, two 5-min washes, followed by two 30-min washes, and finally one 5-min wash. Samples were then mounted on μ-Slide 18-well chambers (81811-IBI, ibidi) and immobilized with 1% low-gelling-temperature agar (Merck/Sigma). When the agar solidified, 18% Optiprep (D1556-250ML, Sigma) in RNase-free PBS was added as a mounting medium and organoids were imaged in the following week.

HCR probes were designed and synthesized by Molecular Instruments and their sequence is confidential (Supplementary Table 10).

### Imaging in situ HCR-stained organoids

Neural organoids were imaged using a Zeiss LSM 980 system in lambda scanning mode followed by spectral unmixing and processed with Fiji. Acquisition mode parameters were: pinhole size, 20 μm (increased light collection in case of very low signal); scan speed, 6; bidirectional scanning, 2× averaging per frame; averaging method, sum intensity, 8 bits per pixel. A water-immersion, ×10 objective was used to take *z*-scans spanning each entire organoid, with a *z*-step size of 10 μm. Tile scans were taken when organoid size exceeded the size of the field of view, and they were later stitched using the ZEN Blue software (v3.5.093.00008). Laser levels for each probe and fluorophore were adjusted to obtain the maximal dynamic range across all organoids, avoiding signal saturation.

### Organoid dissociation

On day 21 of organoid culture, all the organoids from the same conditions were collected from their individual wells and pooled for dissociation together. In the morphogen screen experiments including multiple hPS cell lines (‘AT3’, ‘OG1’, ‘OG2’, ‘SMG4’ and ‘SMOG1’), cells from organoids from different lines were pooled and their identities were later assigned by comparison to a single-nucleotide polymorphism reference. A–E segments of 21-day-old MiSTR tissue were processed in the same way as organoids.

The Miltenyi neural tissue dissociation kit was consistently used in all experiments. First, tissue from each dissociation pool (hereafter referred to as ‘sample’) was transferred to a 5-ml microcentrifuge tube, where it would be washed twice with 1 ml of HBSS without Ca^2+^ and Mg^2+^ (HBSS w/o, Thermo Scientific). After removing the supernatant, 1 ml of enzyme mix 1 (Enzyme P/Papain + Buffer X from Miltenyi Neural dissociation kit) was added to each sample and incubated at 37 °C in the water bath or inside the incubator for 15 min. In the case of the MiSTR tissue dissociations, the gentleMACS Octo Dissociator (Miltenyi) was used inside the incubator for a milder and more progressive homogenization of the tissue into single cells.

After the first incubation step, 15 μl of enzyme mix 2 (Enzyme A/DNase + Buffer Y from Miltenyi Neural Dissociation kit) were added and the sample was mixed carefully with a wide-bore pipette, then triturated 5–10× with a p1000 pipette and triturated again 5–10× with a p200 before another 15 min of incubation at 37 °C. Samples were then further triturated with a p200 and incubated for another 10 min at 37 °C. If there were still clumps, samples were triturated once more with a p200. At this stage, the quality of the dissociation was monitored by observing 1 μl of cell suspension under the microscope at a magnification of ×10. If there were still clumps, further 5-min incubation and trituration steps would be performed. If the suspension was homogeneous, we would proceed to a filtering step.

For cell suspension filtrations, 20 μm PluriSelect cell strainers were used. These were placed on top of a new 5-ml tube and pre-wet with 500 ml of HBSS w/o before passing the cell suspension through it and washing any trapped cells with 1 ml of HBSS w/o and 1 ml of 0.5% BSA (Miltenyi). From these filtered cell suspensions, 20 μl was taken from each of them to count cells and check their viability. In the meantime, samples were centrifuged once for 5 min at 300*g* using a swing centrifuge to minimize cell loss by concentrating the pellet at the bottom of the tube. After this step, the supernatant was removed, and cells were resuspended in staining buffer (0.5% BSA in PBS^−/−^) at a concentration of 7,777 cells per μl.

### Cell hashing (CITE-seq)

Once all samples were ready at the desired concentration, 45 μl of cell suspension was transferred to a new 1.5-ml microcentrifuge tube (approximately 350,000 cells) and 5 μl of Human TruStain FcXTM Fc Blocking reagent (BioLegend) was added to block unspecific binding of the hashing antibodies. After mixing, the suspension was incubated for 10 min at 4 °C. Right after blocking, 2 μl (1 μg) of the corresponding hashing antibody (TotalSeq-A, BioLegend; Supplementary Table 11) were added to the blocked samples, mixed with a pipette and left to incubate for 30 min at 4 °C. Every 10 min the tubes were mildly shaken to avoid precipitation of cells at the bottom of the tube. When the incubation time was finished, 1 ml of staining buffer was added to dilute each sample and tubes were spun down in a swing centrifuge for 5 min at 300*g* and 4 °C. The supernatant was then removed, and an extra washing step was performed, during which 20 μl of cell suspension was taken out for cell counting and viability checking. After the last washing step, cell pellets were resuspended in staining buffer at a concentration of 1,000 cells per μl. Once all samples were adjusted in concentration, 6–12 samples were pooled at a 1:1 ratio, usually by mixing 10 μl of each of them. The suspension was kept on ice during 10x preparations and 20–25 μl of the hashed pool (20–25,000 cells) was loaded in one lane of the Chromium chip (10x Genomics).

### Single-cell transcriptome (cDNA) library generation

We used the Chromium Next GEM Single Cell 3′ v3/3.1 kit (10x Genomics) to generate single-cell transcriptome libraries from hashed samples. The procedure followed the steps listed in the corresponding 10x protocol (CG000183 Rev A for v3, CG000204 Rev D for v3.1).

Briefly, the Chromium chip and Controller were used to encapsulate single cells in GEMs (nanoliter-scale Gel beads in EMulsion) together with a master mix for reverse transcription of its messenger RNAs and a gel bead functionalized with polydT sequences that capture the cell’s mRNA. In addition, these gel beads also contain a unique molecular identifier (UMI) that individually tags all original RNA molecules and a 10x barcode that labels all cDNAs belonging to the same cell. A TruSeq read 1 adaptor sequence is also included to facilitate transcriptome sequencing in Illumina platforms. Once cells were encapsulated in GEMs, a reverse-transcription step was run to convert polyadenylated mRNAs into full-length cDNA sequences with a UMI and cellular barcode. cDNA products were then purified with magnetic beads (Dynabeads MyOne SILANE, manufactured by Thermo Fisher but included in 10x Genomics kits) and further amplified in an extra PCR step using a partial TruSeq read 1 and partial template-switching oligonucleotide as primers. Another purification step based on construct size was performed with SPRIselect reagent (Beckman Coulter) before quantification and quality control with a Bioanalyzer High Sensitivity chip (2100 Bioanalyzer, Agilent).

One-quarter of the cDNA from each 10x sample was carried over for gene expression library construction. In short, cDNA was fragmented to eliminate the template-switching oligonucleotide incorporated in the previous step and ends were repaired and A-tailed. Reaction products were purified with SPRIselect reagent and TruSeq read 2 adaptors were ligated, one of them being a partial read to provide an overhang for the following PCR. Another cleanup step with SPRIselect was performed before the Sample Index PCR when P5 and P7 sequences (for Illumina sequencing) and sample indices (to allow for multiplexing in the sequencer) were included in the construct. The final product was purified with SPRIselect magnetic beads, and a concentration and quality check were performed by running a Bioanalyzer High Sensitivity chip before sequencing.

### Single-cell hashtag (HTO) library generation

The first steps of HTO library preparation were done synchronously with the cDNA library until the cDNA amplification step of the 10x library workflow. From that point onward, a modified version of the BioLegend protocol was used. The barcoded antibodies are coupled to oligo sequences called hashtags (HTOs), which contain a polyadenylated sequence at their 3′ end that enables hybridization with the poly(dT) capture sequences in GEM beads. In parallel to endogenous mRNAs, HTO sequences were reverse-transcribed, and the resulting cDNA was amplified using as PCR oligonucleotides the partial TruSeq read 1 adaptor (provided in the 10x kit) and an additive primer (a partial TruSeq read 2, sequence provided by BioLegend and synthesized by Integrated DNA Technologies). The additive primer annealed on the 3′ end of the construct and enabled its exponential amplification.

During the post-cDNA amplification cleanup step, hashtag-derived fragments (HTOs) were separated from mRNA-derived fragments (cDNA) based on their size (<180 base pairs) and processed separately to construct a hashtag-specific library. The HTO fraction was subjected to a Sample Index PCR where the P5 adaptor was added through the SI-PCR primer mix (10x kit) together with the P7 adaptor and an index sequence (both contained in a TruSeq D70x_s oligonucleotide, sequence synthesized by Integrated DNA Technologies) to allow for simultaneous sequencing with other libraries. For each sample, reactions contained 5 μl of HTO fraction, 2.5 μl of SI-PCR primer (10 μM stock), 2.5 μl of TruSeq D70x_s primer (10 μM stock), 50 μl of Kapa Hifi Hotstart Ready Mix (Roche) and 40 μl of nuclease-free water (Invitrogen). After the Sample Index PCR, a final SPRIselect-based cleanup was performed before resuspension in nuclease-free water and library quality control and quantification.

### Automatic organoid dissociation, single-cell fixation, scRNA-seq library preparation and sequencing for patterning reproducibility experiment

After day 21 in culture, three organoids per condition per cell line were dissociated individually using the CyBio FeliX liquid handler robot with a thermoshaker. For dissociation, we used a papain-based dissociation kit as for the patterning conditions screen. Each organoid was dissociated using 410 μl of enzyme mix 1 and 6 μl of enzyme mix 2. Then, single-cell suspensions from the same condition were pooled (H1 and H9; WTC and WIBJ2). Each individual single-cell suspension has been followed by fixation and permeabilization procedures, which were performed according to the manufacturer’s specification (ParseBiosciences, cell fixation kit v3, CF100). Then, collected samples were processed for highly multiplexed scRNA-seq using a split-pool combinatorial barcoding kit (ParseBiosciences, WT Mega kit v3, MG100).

### Sequencing and genomic data preprocessing

The pooled libraries were appropriately diluted and sequenced at the Genomics Facility Basel (GFB). Sequencing data were demultiplexed by the GFB, using bcl2fastq version 2.20.422 with the following parameters: --ignore-missing-bcls --ignore-missing-controls --ignore-missing-positions --ignore-missing-filter --no- bgzf-compression --barcode-mismatches 1. 10x libraries were sequenced in SP/S1/S2 flow cells with Illumina NovaSeq technology, using a paired-end 28/10/10/90 or 28/8/0/91 configuration (Read1/IDX i7/IDX i5/Read2). After sample demultiplexing, Cell Ranger v3.1.0 was used with GRCh38-3.0.0 as a reference to derive gene-cell count matrices from the sequencing read (fastq) files of the gene expression library. To obtain hashtag-cell count matrices, CITE-seq-Count (v1.4.3)^25^ was run on the hashtag library sequencing read files with the following parameters: --cbf 1, --cbl 16, --umif 17, --umil 28, and a variable number of targeted cells depending on the loaded cells per experiment (ranging from 12,000 to 16,000 cells).

Parse libraries were sequenced in S2 flow cells with Illumina NovaSeq technology, using a paired-end 64/8/8/58 configuration. We used Parse Biosciences Software (v1.3.1) to demultiplex barcodes, map to hg38 human transcriptome and generate count matrix.

### Demultiplexing of hashing antibody labels

The hashtag-cell count matrices obtained from CITE-seq-Count were preprocessed with a custom-made script, which involved transposition and column/row name adaptation. Once adapted, they were subsetted for the cell barcodes present in both the hashtag and gene expression library and added as an ‘HTO’ assay to normalized Seurat objects. They were then normalized using the NormalizeData function using the centered log-ratio transformation method. After normalization, HTODemux was run on the ‘HTO’ assay with a positive.quantile of 0.99–0.999 (adapted depending on the sample) to obtain a hashtag labeling classification of each cell. The quality of this classification was assessed by visualizing hashtag assignments on *t*-SNE and RidgePlots.

### Demultiplexing of cell line identities

In the morphogen screen experiments where multiple hPS cell lines were pooled (‘AT3’, ‘OG1’, ‘OG2’, ‘SMG4’ and ‘SMOG1’), hPS cell identities were demultiplexed using demuxlet^61^ and the parameters --alpha 0 --alpha 0.5. For patterning reproducibility experiment cell identities were demultiplexed using cellsnp-lite^62^ followed by vireo^62,63^. Unclear cell identities were further deciphered using average expression of Y-chromosome genes.

### Single-cell gene expression data analysis

Gene count matrices from each experiment were preprocessed and analyzed using Seurat (v4.3.0)^64^. A quality-control step was consistently applied by filtering out cells with gene counts lower than 1,000 and mitochondrial gene contents of 10% or higher. The count matrices were then normalized and scaled, either using the functions NormalizeData (logarithmic normalization) and ScaleData in the MiSTR experiments or using SCTransform in the patterning screenings. In all cases, cell cycle scoring was performed to remove the effects of cell cycle variables in the data during the scaling step. In addition, a custom-made function was applied to ignore mitochondrial and ribosomal genes in downstream analysis.

Variable feature selection was performed with the ‘mean.var.plot’ method or within SCTransform, and the obtained variable genes were used as input for PCA calculation. The principal components (PCs) with correlation levels to cell cycle scores higher than 0.3 were excluded from downstream analysis. Cell cycle scoring was performed using the CellCycleScoring() function of Seurat package. A variable number of the other PCs—determined by ElbowPlot visualization of their contribution to dataset variance—were used for further dimensionality reduction with *t*-SNE or UMAP embeddings and for neighbor finding and clustering. The clustering resolution and UMAP parameters were adapted for each dataset to retrieve the most meaningful biological information. In some intermediate steps, a list of relevant patterning genes was provided as input to PC calculation to enhance the visualization of differences in patterning, which would otherwise be masked by other sources of variation such as cell cycle, differentiation stage or metabolic states across organoid cells. Feature, dot plot, heat map and violin plotting were performed with the packages ggplot2 (v3.4.1)^65^, Seurat^64^ and SCpubr (v1.1.2)^66^. Differential gene expression testing was performed with Seurat’s function FindMarkers and the wilcoxauc function from the presto package^67^.

Once each experiment was analyzed separately, the single-cell gene/hashtag data were merged with Seurat or integrated using Harmony^68^, CCA/RPCA^69^ or CSS/RSS^27^. In RSS integration, a pseudo-cell aggregated version was used as a reference. The LabelTransfer function from Seurat was used to project the gene expression data to the developing mouse^70^ and human brain^28^ single-cell transcriptome datasets, and the VoxHunt package (v.1.0.1)^71^ was used for spatial projections to the developing mouse brain, using the Allen Brain Atlas ISH reference.

For reproducibility experiment analysis, we used the Scanpy Python package (v1.10.3). Cells were filtered out on the basis of UMI counts (>750, <20,000) and the fraction of mitochondrial genes (<10). Then transcript counts were normalized to the total number of counts for that cell, multiplied by a scaling factor of 10,000 and subsequently natural log transformed. Then, highly variable genes were estimated and total UMI counts and the fraction of mitochondrial genes were regressed out. RSS embedding was generated as described before. For generating UMAP embeddings and clustering, only RSS embeddings with correlation levels to cell cycle scores less than 0.5 were considered. Clusters were annotated into cell classes and brain regions based on canonical marker expression. Neuron clusters were further annotated based on neurotransmitter transporter expression.

### Forebrain axis scoring

To calculate a forebrain axis score per cell, forebrain progenitors (telencephalic and hypothalamic progenitors) were subsetted from the main Seurat object, as this score would only be representative of the DV axis at this AP axis position. The corresponding expression matrix was extracted for these cells and the union of reference genes involved in DV patterning (known from the literature^72–74^) with the variable features from this dataset. This expression matrix was transposed and converted into a Seurat object to observe genes as the variable of interest. ScaleData, RunPCA and FindNeighbors were run to find gene modules with correlated expression patterns. Next, we took the nearest-neighbor graph embeddings and converted them into a symmetric matrix of neighboring (covarying) genes, which was converted into a summary data frame of correlating genes. The dorsalizing genes were separated from the ventralizing ones, and their nearest-neighbor genes were assigned to the same gene module to then calculate a dorsal and ventral score using Seurat’s function AddModuleScore. DV_score and FBaxis_score were calculated as the difference between the dorsal and ventral scores, and FBaxis_rank as the cellular rankings stemming from the FBaxis_score.

### Pseudo-bulk analysis of conditions

To summarize gene expression for each condition taking cell line and experimental information into account, the Seurat functions ‘AverageExpression’ and ‘AggregateExpression’ and the custom function ‘summarize_data_to_groups’ were used. Taking the Condition_ident_line column of the metadata as a grouping variable, the average normalized data matrix, the aggregated count data matrix and the summarized metadata table were given as input to create a new Seurat object (with the ‘CreateSeuratObject’ function). After this, the same downstream processing as earlier was followed, with SCTransform normalization and scaling, cell cycle score regression, PCA calculation with variable features from the SCT assay and UMAP dimensionality reduction.

### Morphogen–regulon network computation for the patterning condition screen

First, our goal was to retrieve regulons for the patterning condition screen data, specifically for the HES3 cells. To calculate regulons, we used SCENIC^39^ using the pySCENIC implementation^75^ (v0.12.1). We ran the full SCENIC algorithm with default parameters using the clustered v10 motif database on the 38,504 cells from the patterning condition screen data^39^. This led to the detection of 413 regulons (average size, 141 genes). Afterward, we calculated regulon activity scores for every cell using AUCell.

Regulon activity was used as input for the GRNBoost2 algorithm (arboreto package 0.1.6) to identify regulons predictive of morphogen concentration, thereby inferring links between regulons and morphogens. To assess time effects and morphogen interactions, we explicitly included these terms as pseudo-morphogens (39 in total). After inference, we demultiplexed the variables back into single morphogens, annotating edges to indicate whether the detected morphogen–regulon link was influenced by interactions or time-dependent effects. When a morphogen–regulon link was detected multiple times, we retained only the strongest connection based on weight (w).

We also calculated correlations between morphogens and regulon activity scores (area under the curve or AUC). Pearson correlations were calculated for timing and concentration experiments separately. Finally, only morphogen–regulon links with a weight > 200 were kept, and non-neural as well as undifferentiated cluster-associated regulons were removed from the network. To improve readability, the number of regulons per morphogen displayed in the regulon network (Fig. 2) was limited to 55. The layout was computed using layout_as_backbone (keep = 0.3) and plotted using igraph v.1.4.0 and tidygraph v1.2.3.

### Morphogen–regulon network computation for the patterning reproducibility screen

For the patterning reproducibility screen (201,104 cells clustered into 26 clusters) on four different cell lines (H1, WTC, H9, WIBJ2), we ran pySCENIC with the same parameters as the original run. First, we ran pySCENIC 100 times on each cell line separately. For this we subsampled the ‘adata’ object, retaining a maximum of 500 cells per cluster, removing any cells belonging to non-neurectodermal tissues. In addition, because we were interested in cell line differences, we performed 100 SCENIC runs sampling a maximum of 600 cells for every cluster (equally distributed across cell lines). In total, we then summarized these 500 SCENIC runs, generating consensus regulons, summarizing every detected interaction between transcription factor and target genes. This led to 136 consensus regulons (average size of 88 genes). We then scored every cell for all consensus regulons using AUCell.

The regulon activity matrix was then used as input for the GRNBoostv2 algorithm to link morphogens with regulons (as described above). The algorithm was run for every cell line separately. Finally, for every detected link, we calculated the Pearson correlation with morphogen concentration, including interactions. For the correlation calculations, we filtered the data, to only retain conditions detected in all cell lines. Furthermore, we subsampled the data, keeping 500 cells per condition for each cell line (22 conditions). Then we normalized the morphogen concentrations (log and min–max). Finally, we calculated correlations (Pearson) separately for conditions with NIM and neural patterning media (SciPy, 1.12.0). We used the Ramsey RESET test to identify morphogen–regulon links that are non-linear using the statsmodels module in Python (v.0.14.1).

### Analysis of morphogen interactions

To quantify differences between single morphogen conditions and morphogen combinations, the MMD distance was calculated between each pair of conditions using pertpy v.0.9.4. MMD was calculated in 20-dimensional PCA space, derived from RSS embeddings.

To quantify non-additivity of morphogen interactions, we developed the following approach. In the 20-dimensional PCA space mentioned above, centroids for each condition were calculated. Then, for each condition (single and combination), the location of the control centroid was deducted to position control as 0. Then, for each triple of single morphogen and combination, the non-negative linear model was fitted, representing morphogen combination as a linear combination of single morphogen treatments and for each case the residual of linear model fit was calculated. These residuals serve as a measure of non-additivity of morphogen interaction (the higher the residual is, the higher the non-additivity). Residuals were computed using the NNLS function of SciPy.optimize v.1.14.1.

### Quantification of variability

To quantify variability between batches and conditions, we utilized several metrics. We calculated Kullback–Leibler divergence (so-called relative entropy) using the entropy function of SciPy v.1.14.1 comparing distributions of cell-type proportion among conditions; MMD and E-distance to measure distances between distributions of cells between conditions in 20-dimensional PCA space, derived from RSS embeddings, using pertpy v.0.9.4. For all of them, lower value represents higher similarity between compared samples, meaning a higher degree of reproducibility. To estimate variability between batches, we calculated the three metrics mentioned above for each cell line and condition, comparing different batches of the same condition and cell line. To compare variability across conditions, we calculated distances between all unique pairs of samples, which received the same treatment (considering all four tested cell lines). Each condition was characterized by the set of 28 unique calculated distances, whose distributions were visualized using box plots.

### Mapping to primary data and human neural organoid atlas

To map single-cell organoid data to the primary human developing brain atlas, we utilized the approach described previously^24^. Briefly, scArches was used to map scRNA-seq data to the scANVI model of the human developing brain atlas. We trained the model for 100 epochs with default parameters. After mapping we calculated maximum presence scores of each condition for primary cells.

To map single-cell organoid data to the human neural organoid atlas, we utilized the hnoca-tools package^24^. scArches was used to map scRNA-seq data to the scPoli model of the human developing brain atlas. We trained the model for 100 epochs with default parameters. After mapping we calculated maximum presence scores of each condition for organoid atlas cells.

### Estimation of AP and DV scores

To train the models to estimate AP and DV scores, the radial glia subset of a recently published first-trimester developing human brain transcriptomic atlas^28^ was used, which was reprocessed as described previously^24^.

For building models, a radial glial cell subset was used. For the AP axis scoring model, the dissection tissues of the primary radial glial cells were firstly summarized to telencephalon, diencephalon, mesencephalon and rhombencephalon. Cells whose dissection tissue labeling was too broad (for example, head and brain) were assigned to ‘None’. To correct for the potential labeling error due to mis-dissection, a label smoothening procedure was applied by calculating the numbers of neighboring cells dissected from each of the four summarized regions. A weighted proportion (weighted by Jaccard index for neighborhood similarity) was calculated and the cell was reassigned to the region with the highest score. Based on the calibrated regional label, a value of 1, 2, 3 or 4 was assigned to each cell—1 for telencephalon, 2 for diencephalon, 3 for mesencephalon and 4 for rhombencephalon. In total, 5,000 radial glial cells with each of the four values were then subset to train an elastic net model (with glmnet package, default parameters) with the scANVI latent representation as the input.

For the DV axis scoring model, the intuitive dorsal and ventral scores were firstly calculated as the module activity scores of the dorsal organizer markers (*PAX6*, *GLI3*, *BARHL1*, *GAS1*, *EMX1*, *WNT2B*) and ventral organizer markers (*NKX2-1*, *NKX2-2*, *SIX6*, *SIX3*, *SHH*, *RAX*, *DLK1*, *FOXA2*, *SPON1*, *SULF1*) using the AddModuleScores function in Seurat. Both marker sets were derived from the mouse developing brain atlas^70^. The marker-based DV scores were calculated as the difference between the two modules scores (DV). A random subset of 80% of the radial glial cells with absolute values of the marker-based DV scores > 5 was selected to train an elastic net model (with glmnet package, binomial family) to classify dorsal and ventral cells with the scANVI latent representation as the input.

To apply the two scoring models to the organoid data, scArches^76^ was first used to project the organoid data to the scANVI model of the primary atlas to obtain the projected latent representation as described in the previous section. Next, the projected representation was used as the input to the trained AP and DV axis scoring model for prediction (type = ‘link’).

### Statistics and reproducibility

For all datasets, single-cell transcriptome measurements were taken once for each organoid or MiSTR culture after 21 days in culture. For differential composition testing in Fig. 2 and Extended Data Figs. 1 and 2, we applied the R function fisher.test (Fisher’s exact test for count data) to compare the number of cells per cluster in each morphogen condition against the control condition. Tests were separately performed for each cell line because each line presented different cell-type biases. No covariates were tested. *P* values were adjusted for multiple testing using the Bonferroni method. For conditions with adjusted *P* value lower than 0.01, an enrichment score was calculated using the log_2_ of the odds ratio; for all the other conditions, the enrichment score was set to 0 (no enrichment nor depletion of a particular cluster). For visualization purposes, an arbitrary value of ±5 was set as the maximum and minimum enrichment score to display, with all values above and below represented as 5 in the final heat map. Supplementary Table 3 shows the final data frame compiling all Fisher’s test results, *P* values, sample sizes and enrichment scores for all clusters, morphogen treatments and cell lines. The script ‘Morphogen_Screen_Enrichment.R’ contains the code used to generate Supplementary Table 3, and is available on GitHub (https://github.com/quadbio/organoid_patterning_screen/). Since HES3-derived organoids were exposed to all the morphogen treatments in the screen, all figures in the paper refer to their enrichment scores, where gradual variations can be better observed.

### Reporting summary

Further information on research design is available in the Nature Portfolio Reporting Summary linked to this article.

## Online content

Any methods, additional references, Nature Portfolio reporting summaries, source data, extended data, supplementary information, acknowledgements, peer review information; details of author contributions and competing interests; and statements of data and code availability are available at 10.1038/s41592-025-02927-5.

## Supplementary information

Supplementary Information Supplementary Figs. 1–3.

Reporting Summary

Supplementary Table 1 Cluster marker genes for single-cell transcriptomic data from neural organoid morphogen screen (Figs. 1–3).

Supplementary Table 2 Shannon entropy index calculation for each condition. Lower values indicate lower cell-type diversity in the organoids.

Supplementary Table 3 List of enrichment scores for each cluster in each morphogen treatment and cell line with corresponding details (neural organoid morphogen screen; Fig. 2).

Supplementary Table 4 List of regulons associated with each morphogen treatment with corresponding details (HES3 cell line).

Supplementary Table 5 Cluster marker genes for single-cell transcriptomic data from neural organoid reproducibility screen (Figs. 4 and 5).

Supplementary Table 6 List of enrichment scores for each cluster in each morphogen treatment and cell line with corresponding details (neural organoid reproducibility screen; Figs. 4 and 5).

Supplementary Table 7 Culture conditions for MiSTR-like organoids. The morphogen dose given to each condition is stated in column ‘Control-E’.

Supplementary Table 8 Cluster marker genes for single-cell transcriptomic data from MiSTR-versus-MiSTR-like organoids experiment (Fig. 6).

Supplementary Table 9 Overview of current neural organoid protocols, with their corresponding neural induction method and patterning molecules used for regionalization. Some of the molecules included in the Neural Induction section may have effects on patterning, but they were placed in the section corresponding to the intended use by the authors. Molecules between square brackets were applied at the same time points and molecules between parentheses are dispensable for obtaining the corresponding regional fates. SB-431542, A83-01 = TGFB inhibitors; Noggin, Dorsomorphin, LDN-193819 = BMP inhibitors; DKK1, IWP-2, IWR-1-e = WNT inhibitors; CHIR99021, BIO = WNT activator; cyclopamine = SHH inhibitor; SAG, PM = SHH activators; SR11237; SU5402, PD325901 = FGF inhibitor; Activin-A, Nodal = TGFB agonist; LIF, leukemia inhibitory factor; RPE, retinal pigmented epithelium; NR, neural retina; MGE, medial ganglionic eminence; LGE, lateral ganglionic eminence; RA, retinoic acid.

Supplementary Table 10 HCR probes from Molecular Instruments (confidential sequence).

Supplementary Table 11 List of all the TotalSeq-A antibodies used for cell hashing and their corresponding barcode sequence.

## Data Availability

Raw and processed sequencing data are available at ArrayExpress. The accession codes for the individual experiments are E-MTAB-15622 for the morphogen reproducibility screen and E-MTAB-15667 for the morphogen patterning screen. Processed data and the VCF files for demultiplexing are available on Zenodo via 10.5281/zenodo.17225179 (ref. ^77^).

## Code availability

Scripts reproducing the main analyses in this study are available on GitHub (https://github.com/quadbio/organoid_patterning_screen/).
