# Supplementary material for: Systematic scRNA-seq screens profile neural organoid response to morphogens
Source: Nat Methods. 2025 Dec 15;23(2):465–78. doi: 10.1038/s41592-025-02927-5 (PMC12904787; doi:10.1038/s41592-025-02927-5)
Supplement: Supplementary file 1 — Supplementary Figs. 1–3. [file 41592_2025_2927_MOESM1_ESM.pdf]

nature methods

Supplementary information https://doi.org/10.1038/s41592-025-02927-5

Systematic scRNA-seq screens profile neural organoid response to morphogens

In the format provided by the authors and unedited

Supplementary information guide

Supplementary Figures

Supplementary Figure 1. Experimental conditions for the probing of morphogen timing, concentrations and combinations in human neural organoids (patterning condition screen). In the "Experiment" column, the ID used in the Seurat object is also indicated. The number of assayed organoids per morphogen treatment from each hESC/hiPSC line is indicated in the last column. The right section of the table depicts a summary of the patterning protocol for each condition, with the culture days indicated at the top. In the Concentration experiments, colour intensity represents morphogen concentrations (higher intensity means higher concentration). In the Timing and Combination experiments, colours were kept as the most intense hue to facilitate visualization.

Supplementary Figure 2. Brightfield images of all organoids cultured in the patterning condition screen.

Supplementary Figure 3. Brightfield images of all organoids cultured in the patterning reproducibility screen.

Supplementary Figure 1

Experiment (ID)	Morphogen	Dose	Timing	Organoids (cell line)
Timing 1 (AT1)	Retinoic Acid	250 nM	Days 0-3	8 (HES3)
	Retinoic Acid	250 nM	Days 5-8	8 (HES3)
	Retinoic Acid	250 nM	Days 10-13	8 (HES3)
	Retinoic Acid	250 nM	Days 14-17	8 (HES3)
	Retinoic Acid	250 nM	Days 17-20	8 (HES3)
	XAV939	180 nM	Days 0-3	8 (HES3)
	XAV939	180 nM	Days 5-8	8 (HES3)
	XAV939	180 nM	Days 10-13	8 (HES3)
	XAV939	180 nM	Days 14-17	8 (HES3)
	XAV939	180 nM	Days 17-20	7 (HES3)
Timing 2 (AT2)	FGF8	100 ng/mL	Days 0-3	6 (HES3)
	FGF8	100 ng/mL	Days 5-8	6 (HES3)
	FGF8	100 ng/mL	Days 10-13	6 (HES3)
	FGF8	100 ng/mL	Days 14-17	6 (HES3)
	FGF8	100 ng/mL	Days 17-20	6 (HES3)
	SHH	200 ng/mL	Days 0-3	5 (HES3)
	SHH	200 ng/mL	Days 5-8	5 (HES3)
	SHH	200 ng/mL	Days 10-13	5 (HES3)
	SHH	200 ng/mL	Days 14-17	5 (HES3)
	SHH	200 ng/mL	Days 17-20	5 (HES3)
Timing 3 (AT3)	BMP4	5 nM	Days 0-3	4 (WTC)
	BMP4	5 nM	Days 5-8	4 (WTC) 4 (HES3) 4 (H9)
	BMP4	5 nM	Days 10-13	4 (WTC) 4 (HES3) 4 (H9)
	BMP4	5 nM	Days 14-17	4 (WTC) 4 (HES3) 3 (H9)
	BMP4	5 nM	Days 17-20	4 (WTC) 4 (HES3) 4 (H9)
	BMP7	5 nM	Days 0-3	4 (WTC) 4 (HES3)
	BMP7	5 nM	Days 5-8	4 (WTC) 4 (HES3) 4 (H9)
	BMP7	5 nM	Days 10-13	4 (WTC) 4 (HES3) 4 (H9)
	BMP7	5 nM	Days 14-17	4 (WTC) 4 (HES3) 4 (H9)
	BMP7	5 nM	Days 17-20	4 (WTC) 4 (HES3) 4 (H9)

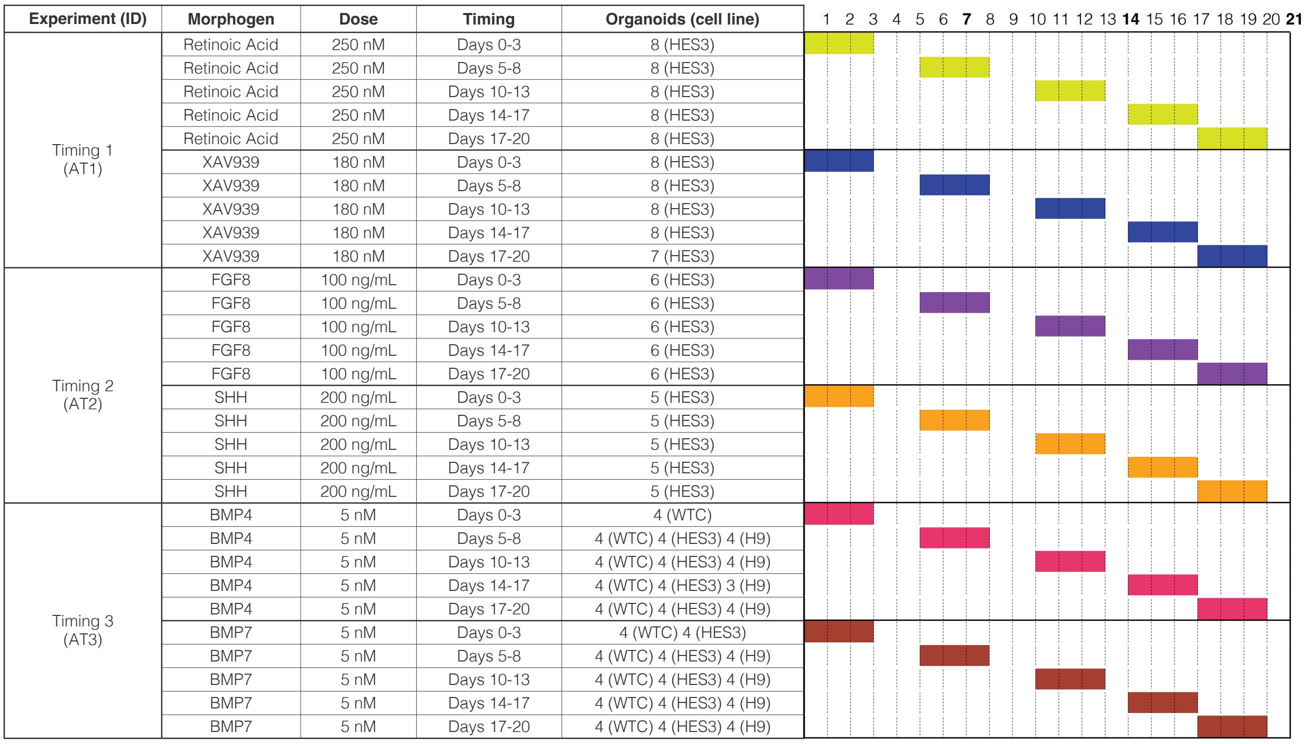

Experiment	Morphogen	Dose	Timing	Organoids (cell line)
Concentration 1 (SMG1)	Retinoic Acid	100 nM	Days 9-20	8 (HES3)
	Retinoic Acid	200 nM	Days 9-20	8 (HES3)
	Retinoic Acid	300 nM	Days 9-20	8 (HES3)
	Retinoic Acid	400 nM	Days 9-20	8 (HES3)
	Retinoic Acid	500 nM	Days 9-20	8 (HES3)
	SHH + PM	20 ng/mL + 0.03 µM	Days 3-14	7 (HES3)
	SHH + PM	60 ng/mL + 0.09 µM	Days 3-14	8 (HES3)
	SHH + PM	100 ng/mL + 0.15 µM	Days 3-14	8 (HES3)
	SHH + PM	140 ng/mL + 0.21 µM	Days 3-14	8 (HES3)
	SHH + PM	180 ng/mL + 0.27 µM	Days 3-14	8 (HES3)
Concentration 2 (SMG2)	FGF8	50 ng/mL	Days 3-14	8 (HES3)
	FGF8	100 ng/mL	Days 3-14	8 (HES3)
	FGF8	150 ng/mL	Days 3-14	8 (HES3)
	FGF8	200 ng/mL	Days 3-14	8 (HES3)
	FGF8	250 ng/mL	Days 3-14	8 (HES3)
	CHIR	0.2 µM	Days 0-9	8 (HES3)
	CHIR	0.6 µM	Days 0-9	8 (HES3)
	CHIR	1 µM	Days 0-9	8 (HES3)
	CHIR	1.4 µM	Days 0-9	8 (HES3)
	CHIR	1.8 µM	Days 0-9	8 (HES3)
Concentration 3 (SMG4)	BMP4	0.5 nM	Days 12-20	4 (WTC) 4 (HES3) 4 (H9)
	BMP4	1.5 nM	Days 12-20	4 (WTC) 4 (HES3) 4 (H9)
	BMP4	2.5 nM	Days 12-20	4 (WTC) 4 (HES3) 4 (H9)
	BMP4	3.5 nM	Days 12-20	4 (WTC) 4 (HES3) 4 (H9)
	BMP4	4.5 nM	Days 12-20	4 (WTC) 4 (HES3) 4 (H9)
	BMP7	0.5 nM	Days 12-20	4 (WTC) 4 (HES3) 4 (H9)
	BMP7	1.5 nM	Days 12-20	4 (WTC) 4 (HES3) 4 (H9)
	BMP7	2.5 nM	Days 12-20	4 (WTC) 4 (HES3) 4 (H9)
	BMP7	3.5 nM	Days 12-20	4 (WTC) 4 (HES3) 4 (H9)
	BMP7	4.5 nM	Days 12-20	4 (WTC) 4 (HES3) 4 (H9)
Concentration 4 (SMOG1)	FGF8	50 ng/mL	Days 12-20	4 (WTC) 4 (HES3) 4 (H9)
	FGF8	100 ng/mL	Days 12-20	4 (WTC) 4 (HES3) 4 (H9)
	FGF8	150 ng/mL	Days 12-20	4 (WTC) 4 (HES3) 4 (H9)
	FGF8	200 ng/mL	Days 12-20	4 (WTC) 4 (HES3) 4 (H9)
	FGF8	250 ng/mL	Days 12-20	4 (WTC) 4 (HES3) 4 (H9)
Concentration 5 (OG1)	Cyclopamine	150 nM	Days 3-14	4 (WTC) 4 (HES3)
	XAV939	5 µM	Days 0-9	4 (WTC) 4 (HES3)
	XAV939	1 µM	Days 0-9	4 (WTC) 4 (HES3)

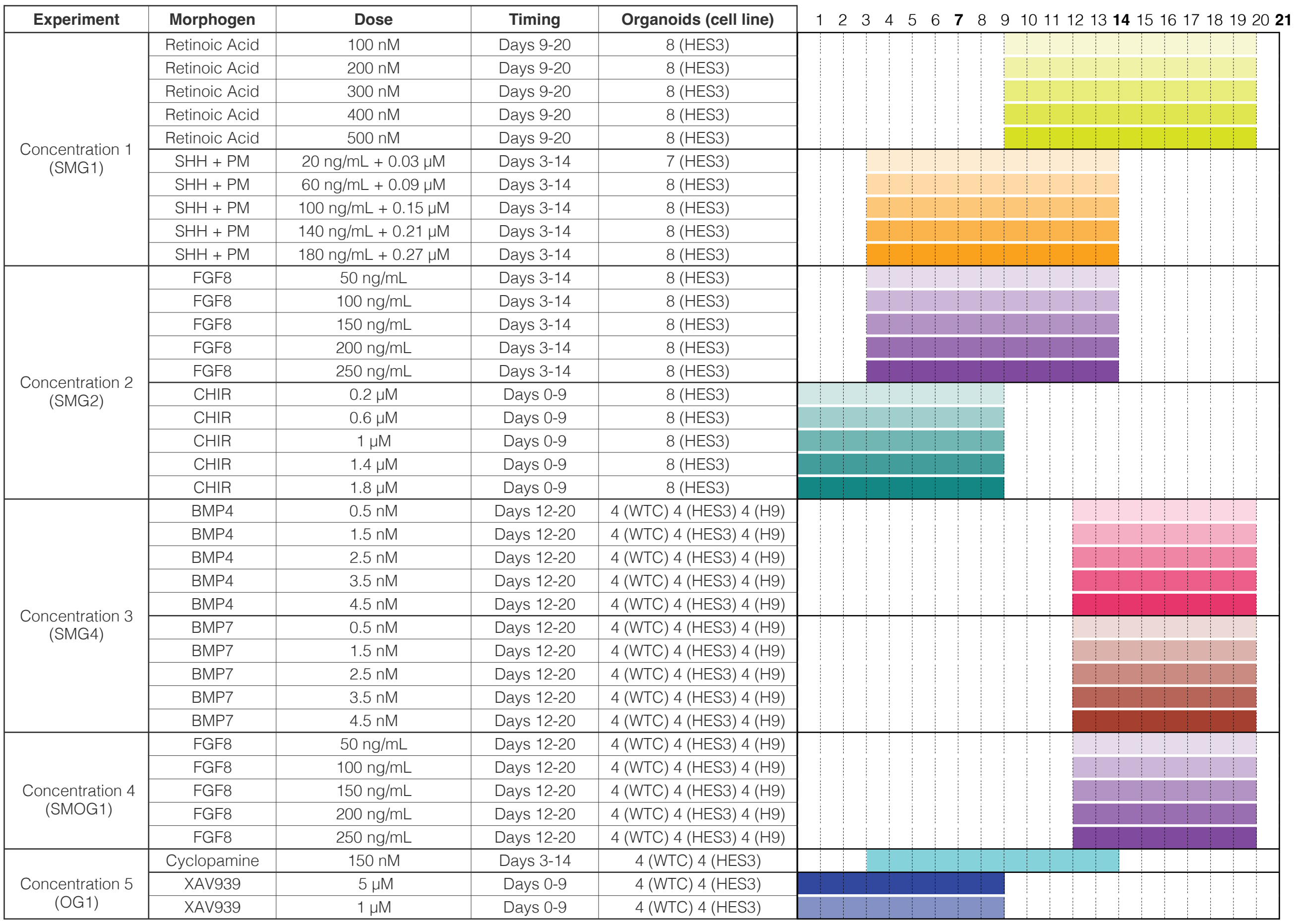

Experiment	Morphogen 1	Dose 1	Timing 1	Morphogen 2	Dose 2	Timing 2	Morphogen 3	Dose 3	Timing 3	Organoids (cell line)
Combination 1 (OG1)	Cyclopamine	150 nM	Days 3-14	XAV939	5 µM	Days 0-9	-	-	-	4 (WTC) 4 (HES3)
	Cyclopamine	150 nM	Days 3-14	XAV939	1 µM	Days 0-9	-	-	-	4 (WTC) 4 (HES3)
	Cyclopamine	150 nM	Days 3-14	RA	500 µM	Days 9-20	-	-	-	4 (WTC) 4 (HES3)
	Cyclopamine	150 nM	Days 3-14	CHIR	1.8 µM	Days 0-9	-	-	-	4 (WTC) 4 (HES3)
	SHH + PM	20 ng/mL + 0.03 µM	Days 3-14	XAV939	5 µM	Days 0-9				4 (WTC) 4 (HES3)
	SHH + PM	20 ng/mL + 0.03 µM	Days 3-14	XAV939	1 µM	Days 0-9	-	-	-	4 (WTC) 4 (HES3)
Combination 2 (OG2)	SHH + PM	20 ng/mL + 0.03 µM	Days 3-14	RA	500 µM	Days 9-20	-	-	-	4 (WTC) 4 (HES3)
	SHH + PM	20 ng/mL + 0.03 µM	Days 3-14	CHIR	1.8 µM	Days 0-9	-	-	-	4 (WTC) 4 (HES3)
	SHH + PM	180 ng/mL + 0.27 µM	Days 3-14	XAV939	5 µM	Days 0-9	-	-	-	4 (WTC) 4 (HES3)
	SHH + PM	180 ng/mL + 0.27 µM	Days 3-14	XAV939	1 µM	Days 0-9	-	-	-	4 (WTC) 4 (HES3)
	SHH + PM	180 ng/mL + 0.27 µM	Days 3-14	RA	500 µM	Days 9-20	-	-	-	4 (WTC) 4 (HES3)
	SHH + PM	180 ng/mL + 0.27 µM	Days 3-14	CHIR	1.8 µM	Days 0-9	-	-	-	4 (WTC) 4 (HES3)
	Cyclopamine	150 nM	Days 3-14	XAV939	5 µM	Days 0-9	FGF8	50 ng/mL	Days 12-20	4 (WTC) 4 (HES3)
	-	-	-	XAV939	5 µM	Days 0-9	FGF8	50 ng/mL	Days 12-20	4 (WTC) 4 (HES3)
	SHH + PM	20 ng/mL + 0.03 µM	Days 3-14	XAV939	5 µM	Days 0-9	FGF8	50 ng/mL	Days 12-20	4 (WTC) 4 (HES3)
	Cyclopamine	150 nM	Days 3-14	RA	500 µM	Days 9-20	FGF8	50 ng/mL	Days 12-20	4 (WTC) 4 (HES3)
	-	-	-	RA	500 µM	Days 9-20	FGF8	50 ng/mL	Days 12-20	4 (WTC) 4 (HES3)
	SHH + PM	20 ng/mL + 0.03 µM	Days 3-14	RA	500 µM	Days 9-20	FGF8	50 ng/mL	Days 12-20	4 (WTC) 4 (HES3)

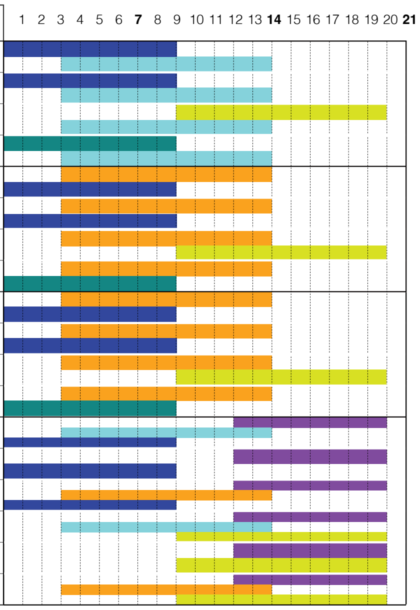

Supplementary Figure 2

NKX2.1-GFP HES3
Timing experiment

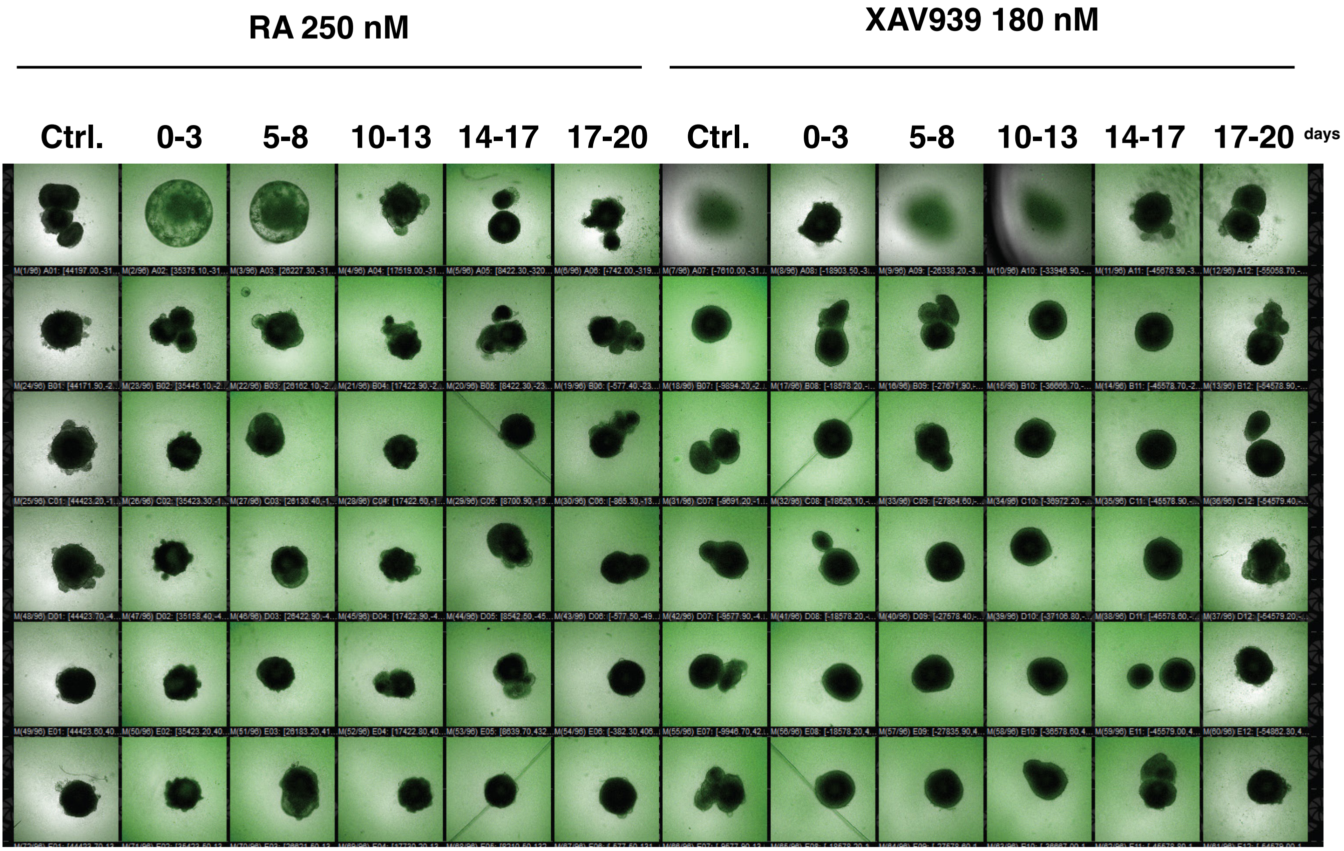

5nM BMP4

Timing experiment

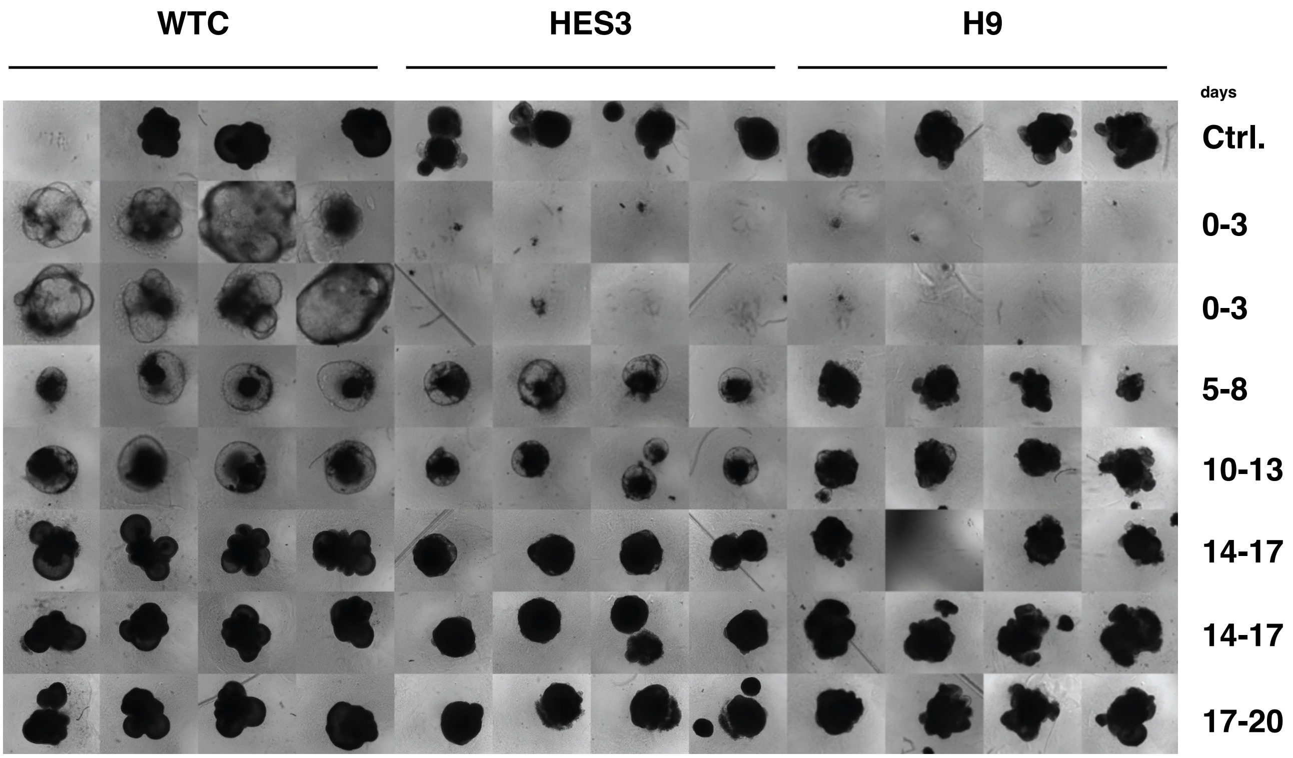

5nM BMP7

Timing experiment

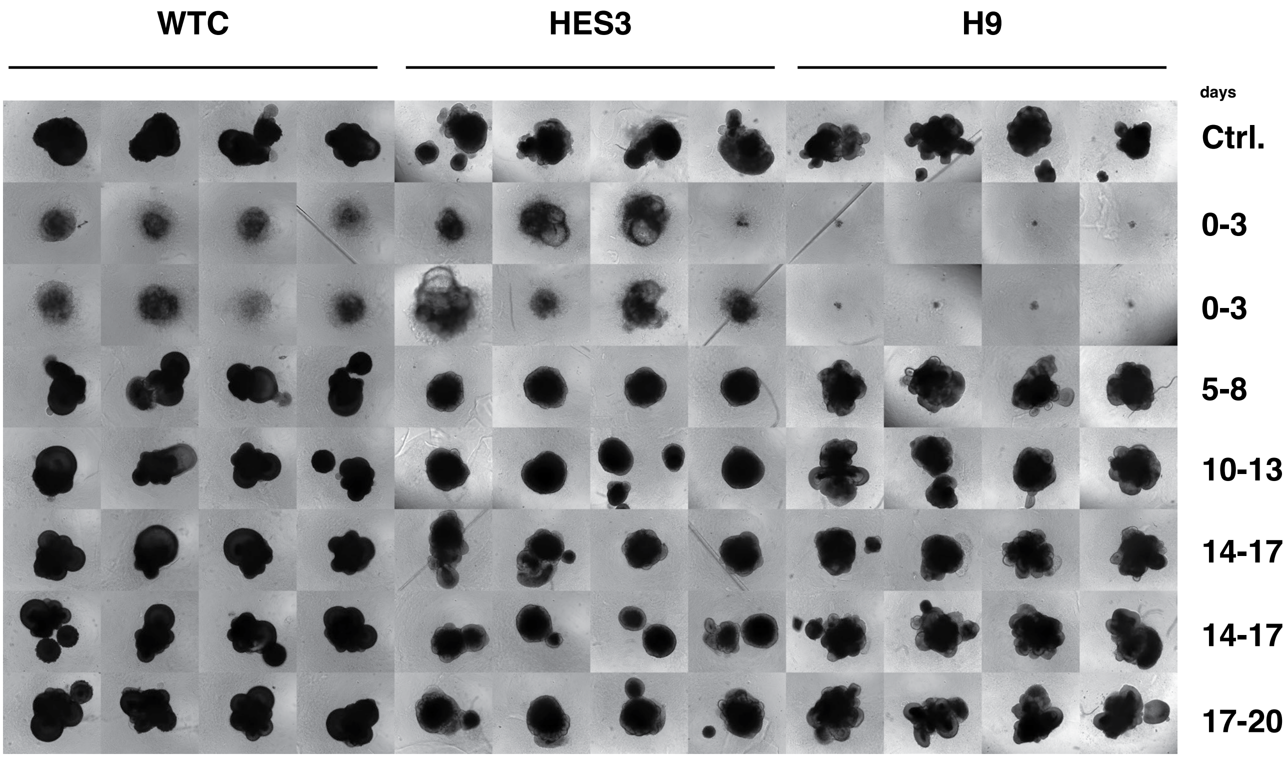

NKX2.1-GFP HES3

Concentration experiment

SHH + PM						RA					
Ctrl.	20ng/mL 0.03uM	60ng/mL 0.09uM	100ng/mL 0.15uM	140ng/mL 0.21uM	180ng/mL 0.27uM	Ctrl.	100nM	200nM	300nM	400nM	500nM

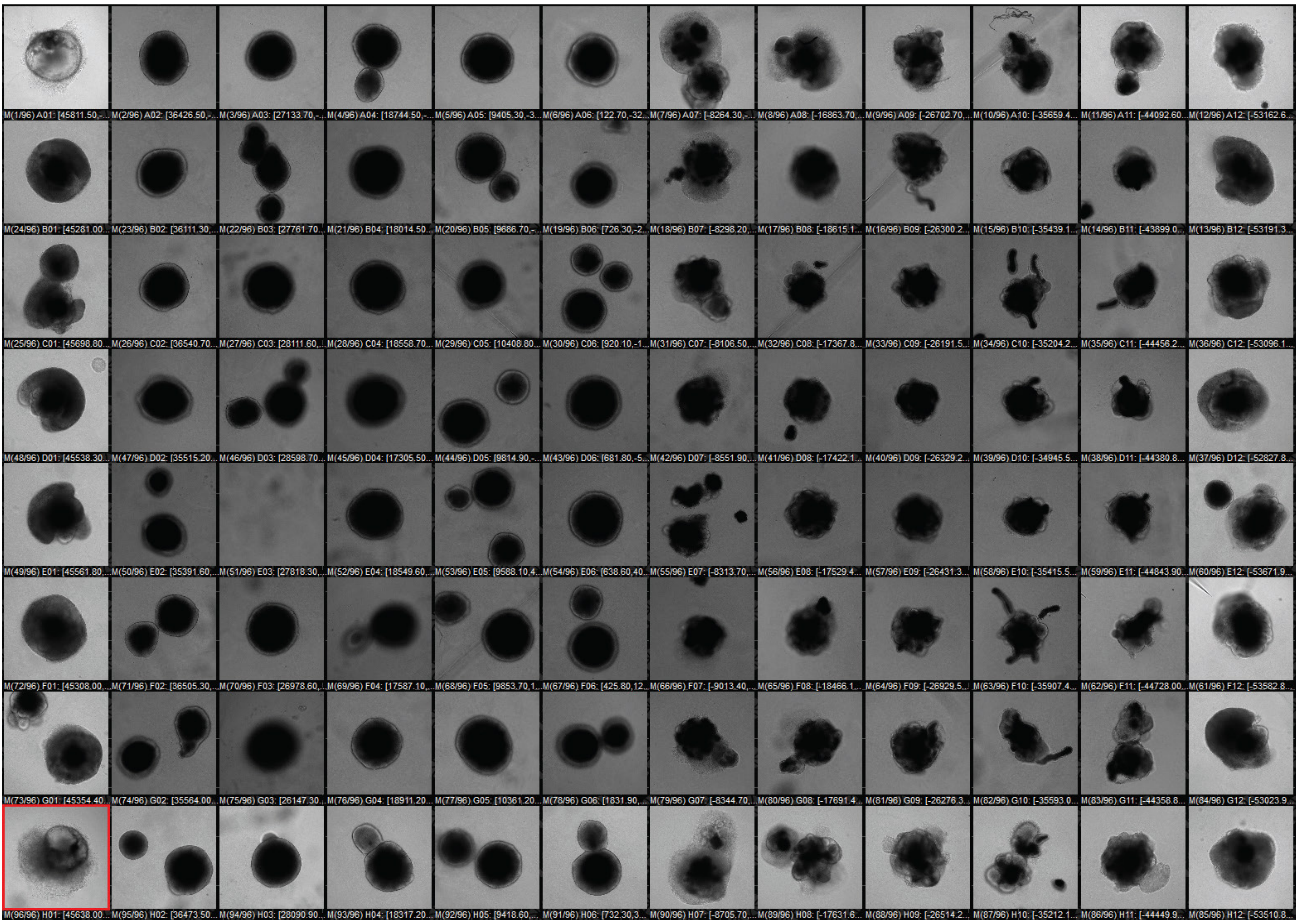

NKX2.1-GFP HES3
Concentration experiment

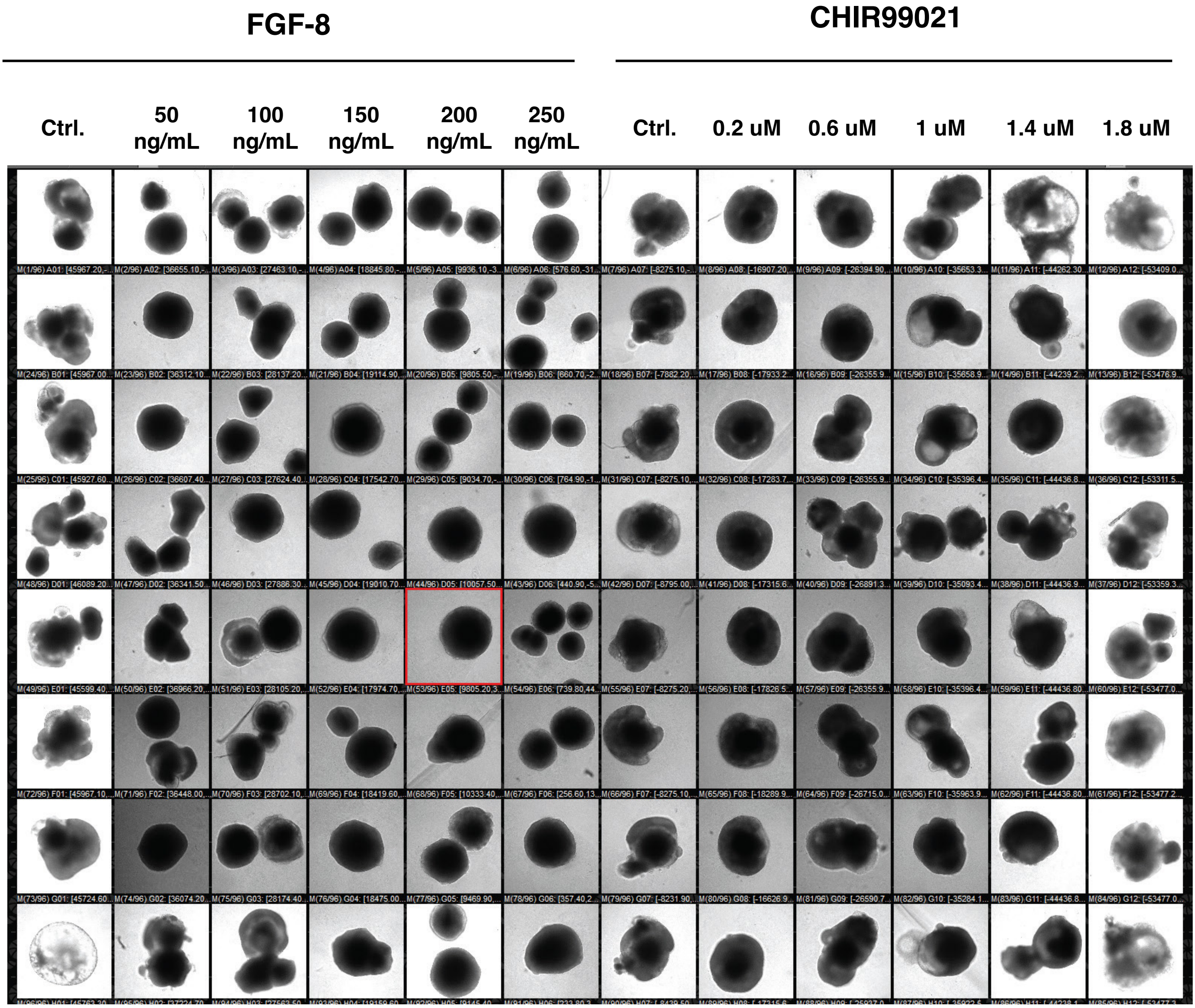

BMP4

Concentration experiment

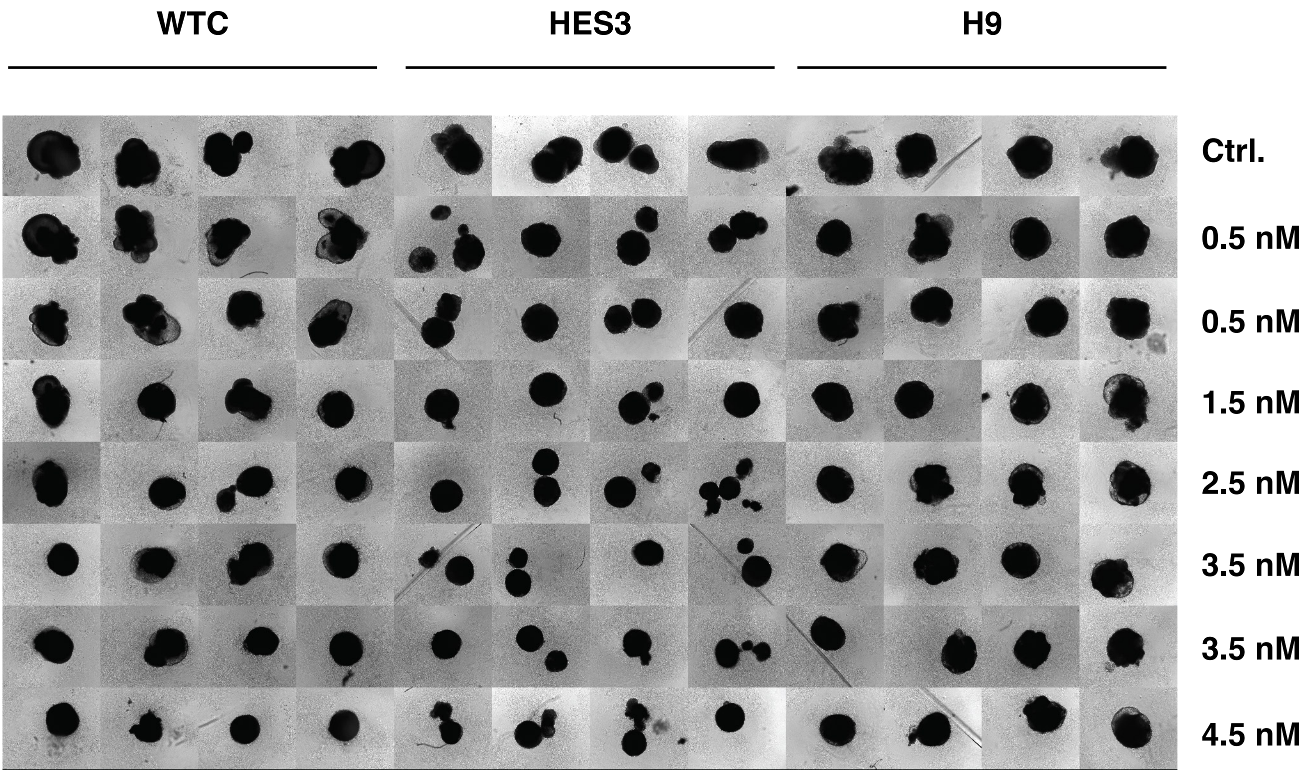

BMP7
Concentration experiment

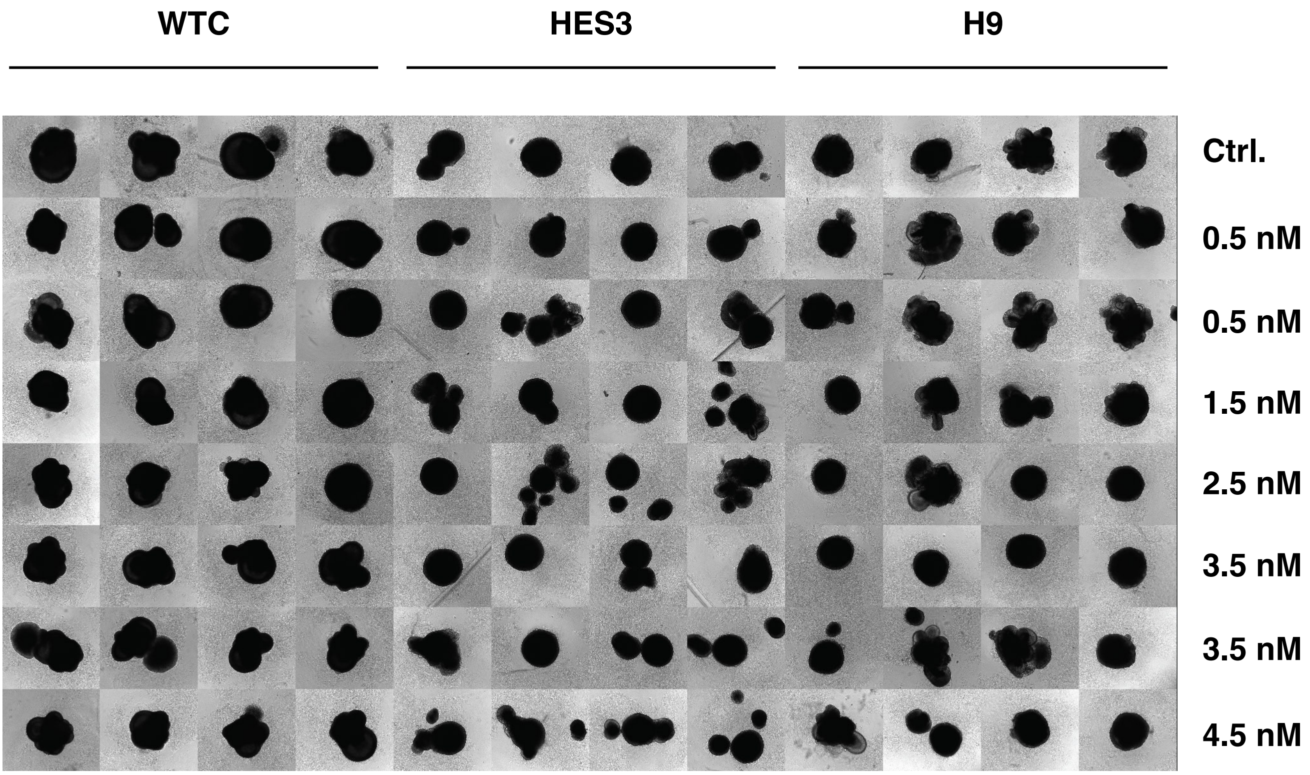

Concentration experiment

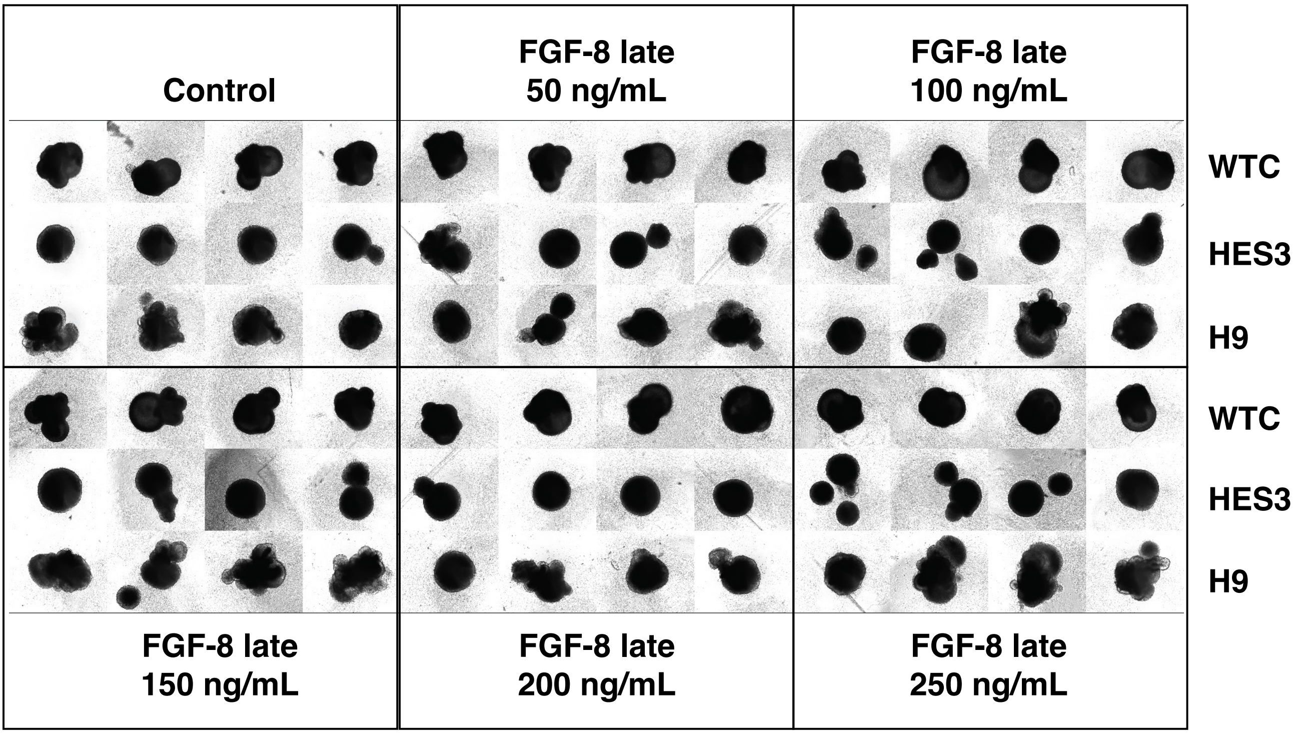

Combination experiment

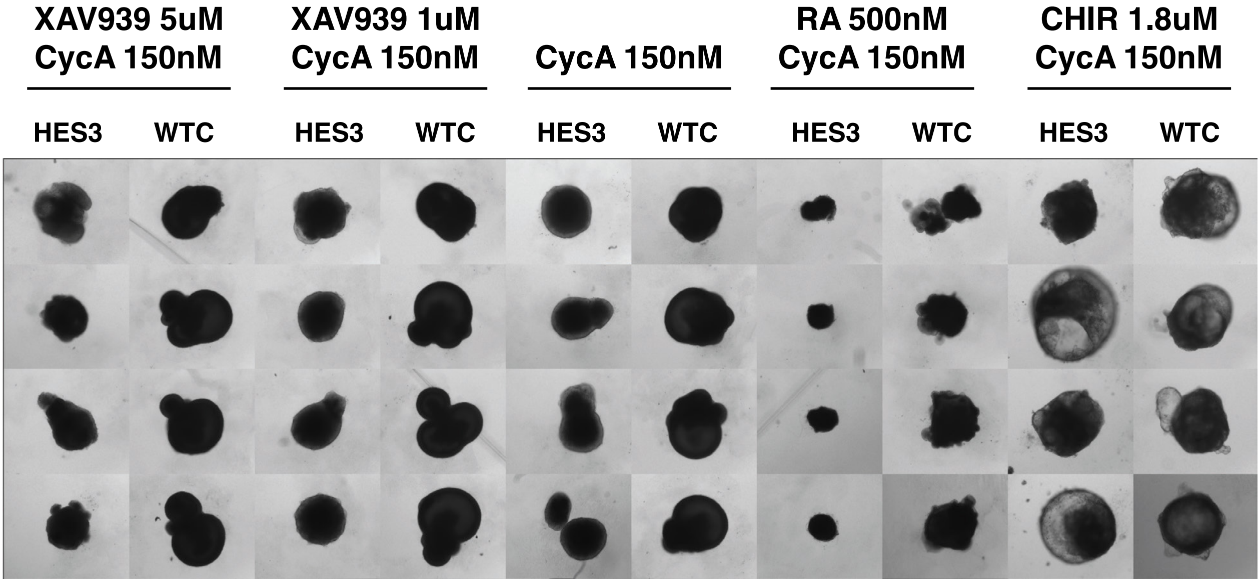

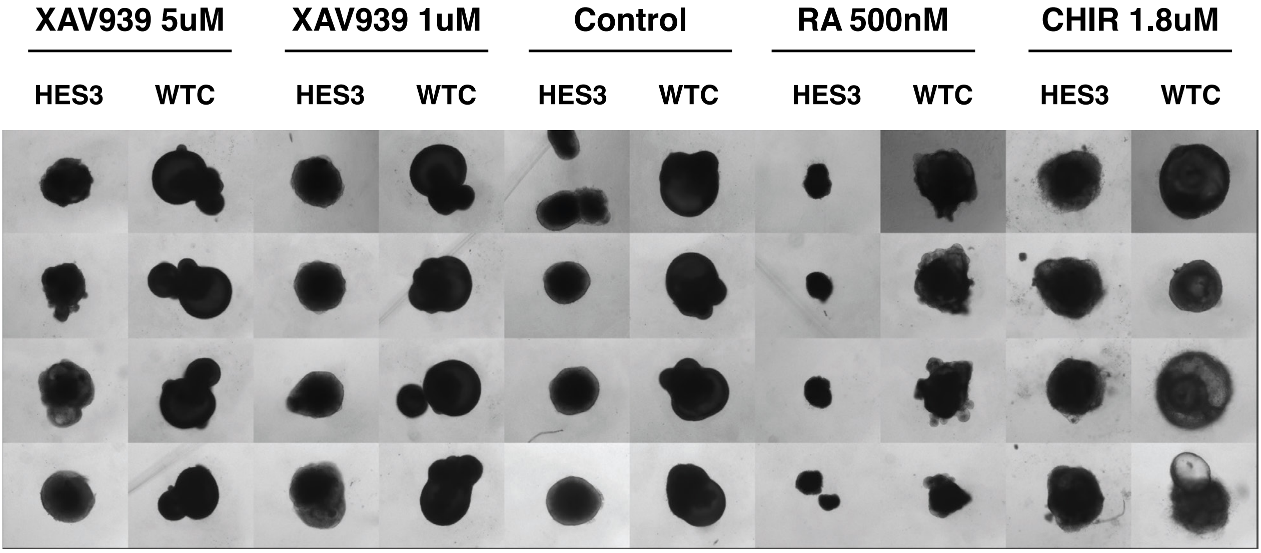

Combination experiment

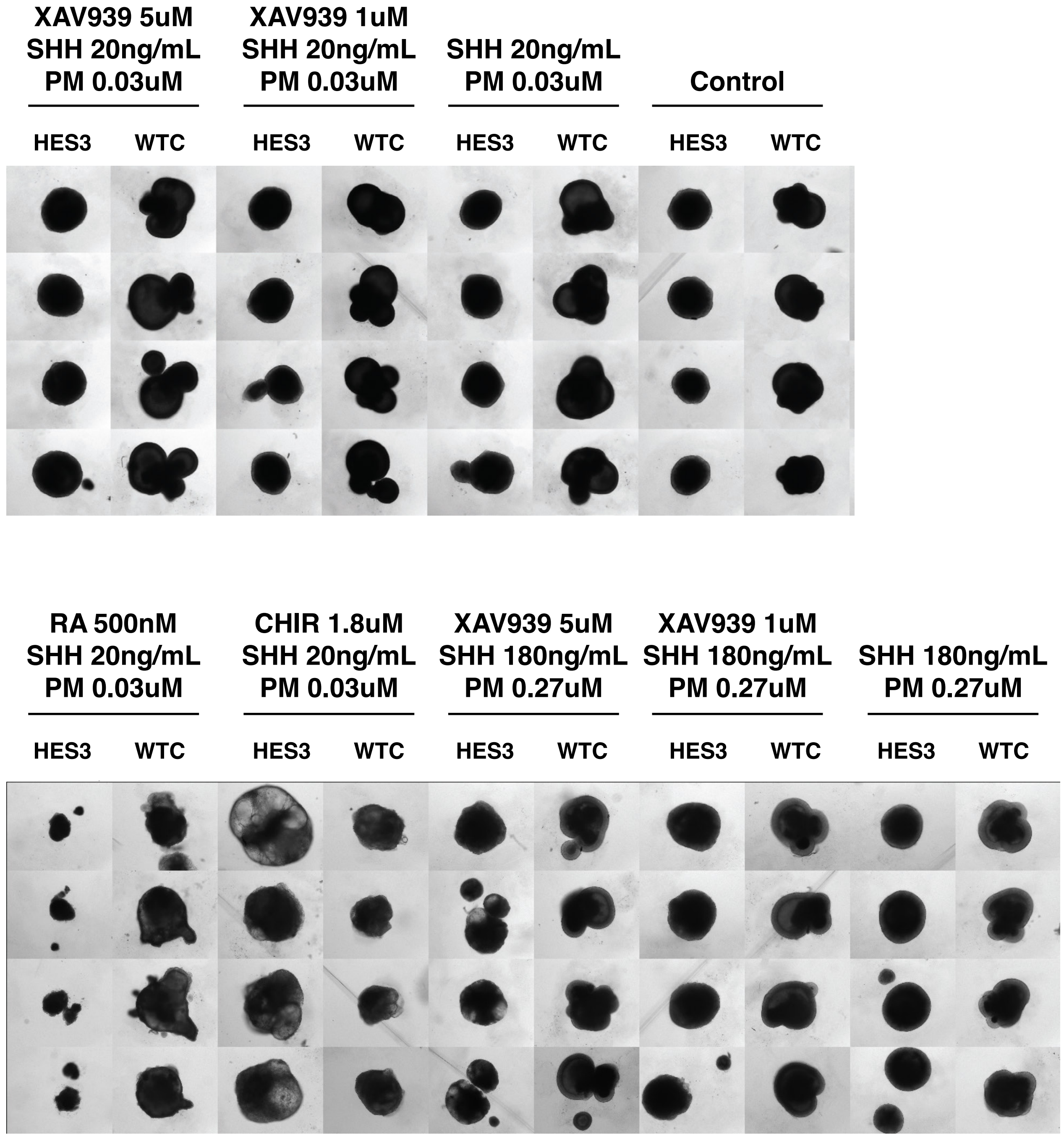

Combination experiment

RA 500nM<br>SHH 180ng/mL<br>PM 0.27uM		CHIR 1.8uM<br>SHH 180ng/mL<br>PM 0.27uM		FGF-8 50ng/mL<br>XAV939 5uM<br>CycA 150nM		FGF-8 50ng/mL<br>XAV939 5uM		FGF-8 50ng/mL<br>XAV939 5uM<br>SHH 180ng/mL<br>PM 0.27uM	
HES3	WTC	HES3	WTC	HES3	WTC	HES3	WTC	HES3	WTC

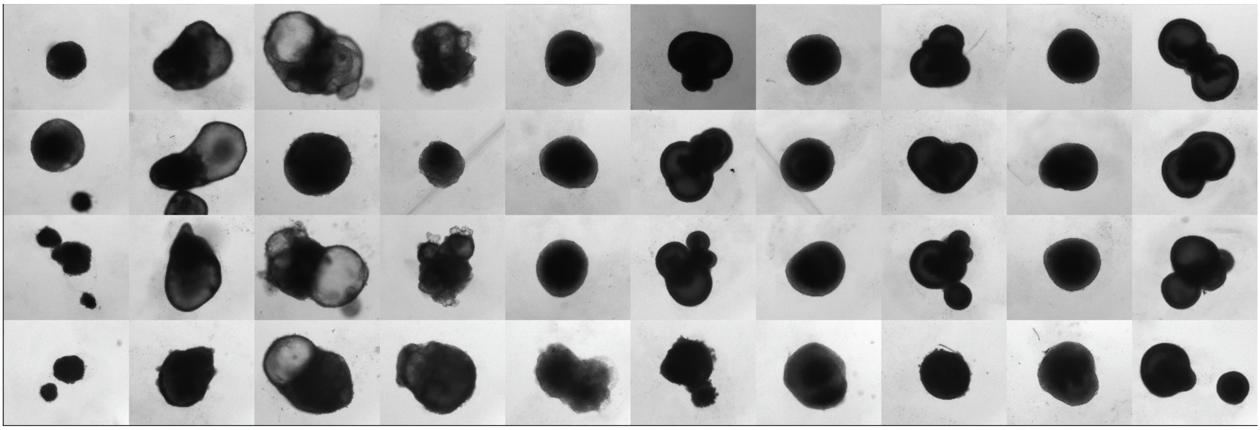

RA 500nM<br>FGF-8 50ng/mL<br>CycA 150nM		RA 500nM<br>FGF-8 50ng/mL		RA 500nM<br>FGF-8 50ng/mL<br>SHH 180ng/mL<br>PM 0.27uM		Control	
HES3	WTC	HES3	WTC	HES3	WTC	HES3	WTC

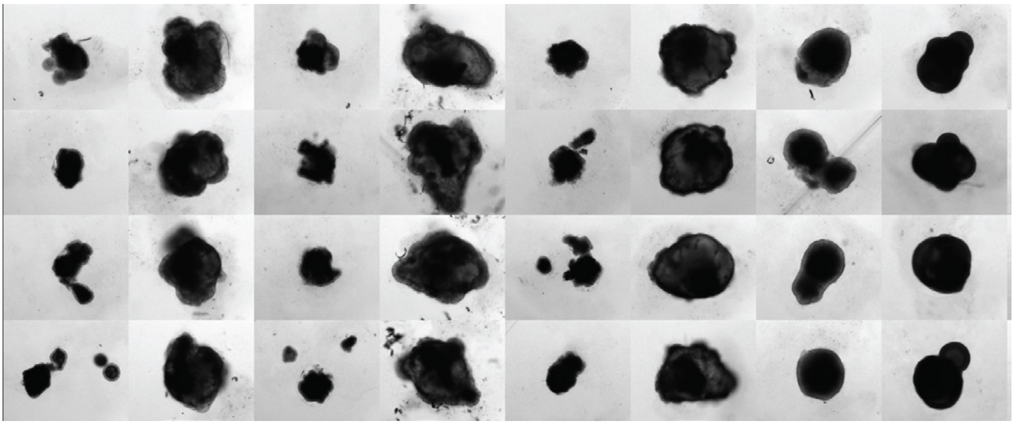

Supplementary Figure 3

H9

Minimal NIM

Control | SHH c_1 | SHH c_5 | RA c_1 | RA c_5 | CHIR c_1 | CHIR c_5 | FGF-8 c_1 | FGF-8 c_5 | CHIR t_1 | CHIR t_3 | CHIR t_5

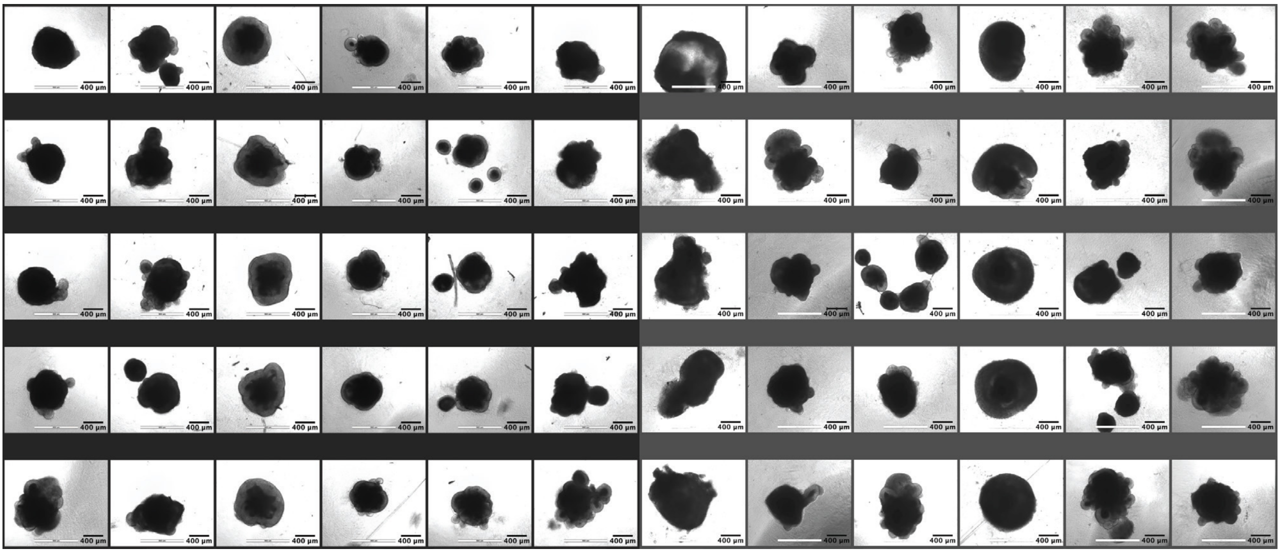

Dual SMAD

Control | SHH c_1 | SHH c_5 | RA c_1 | RA c_5 | CHIR c_1 | CHIR c_5 | FGF-8 c_1 | FGF-8 c_5 | CHIR t_1 | CHIR t_3 | CHIR t_5

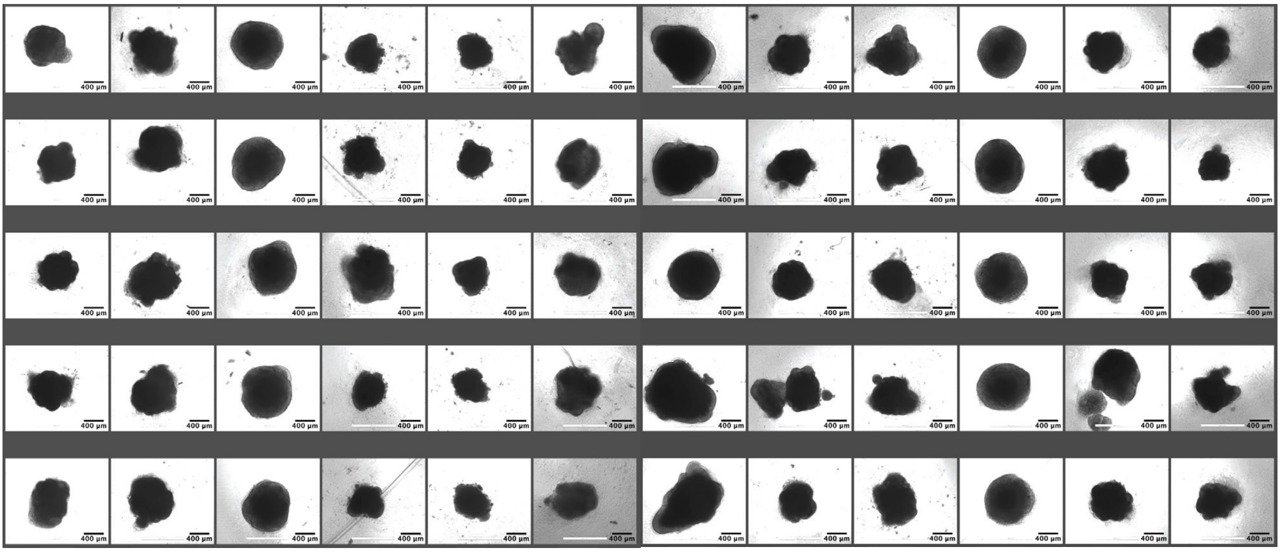

H1

Minimal NIM

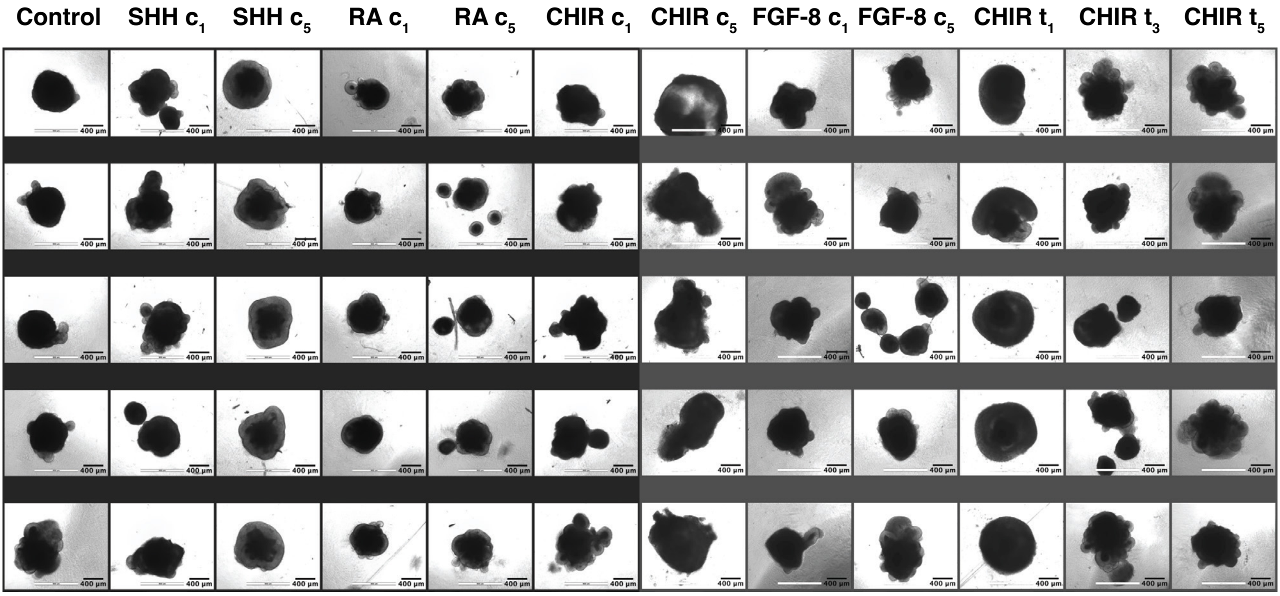

Dual SMAD

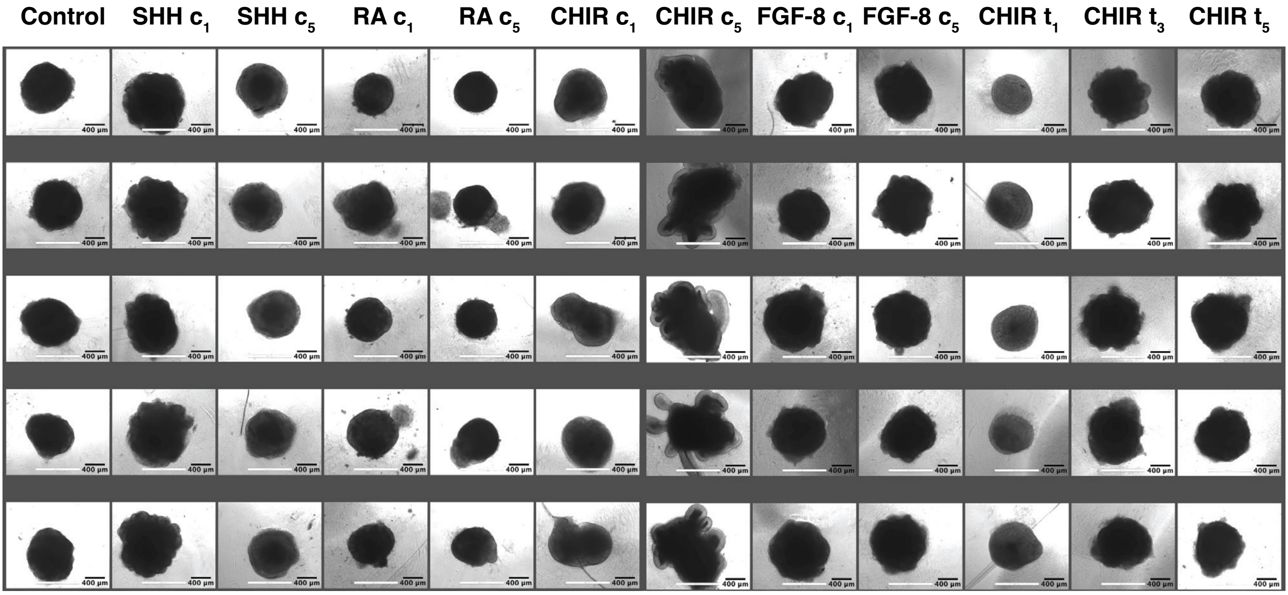

WIBJ2

Minimal NIM

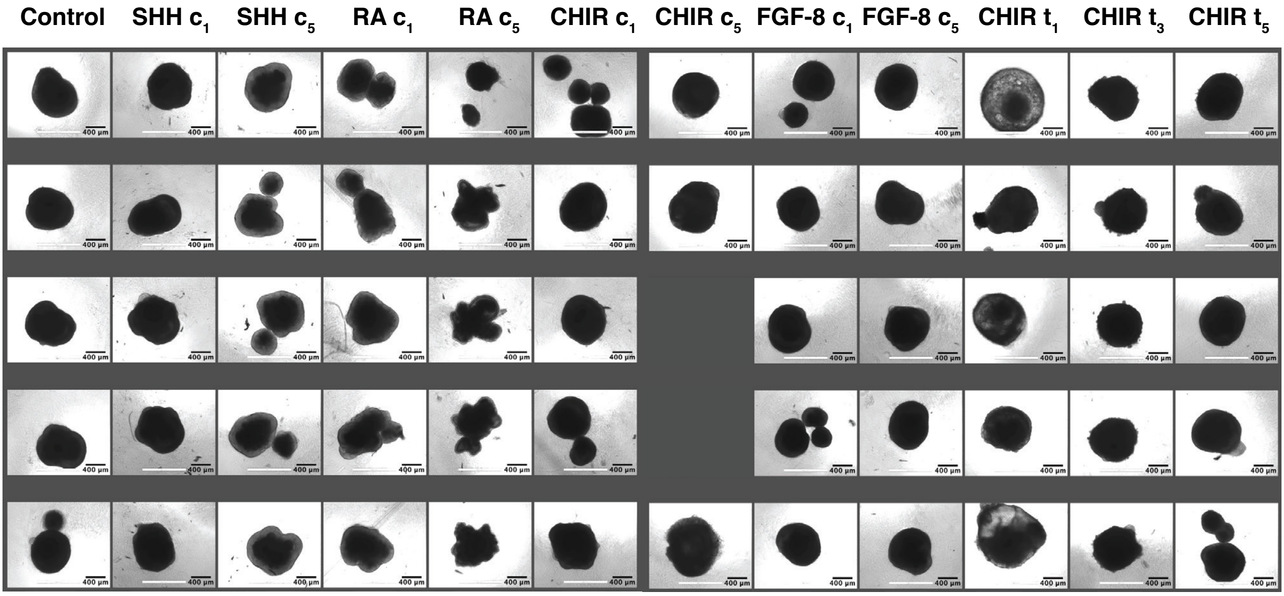

Dual SMAD

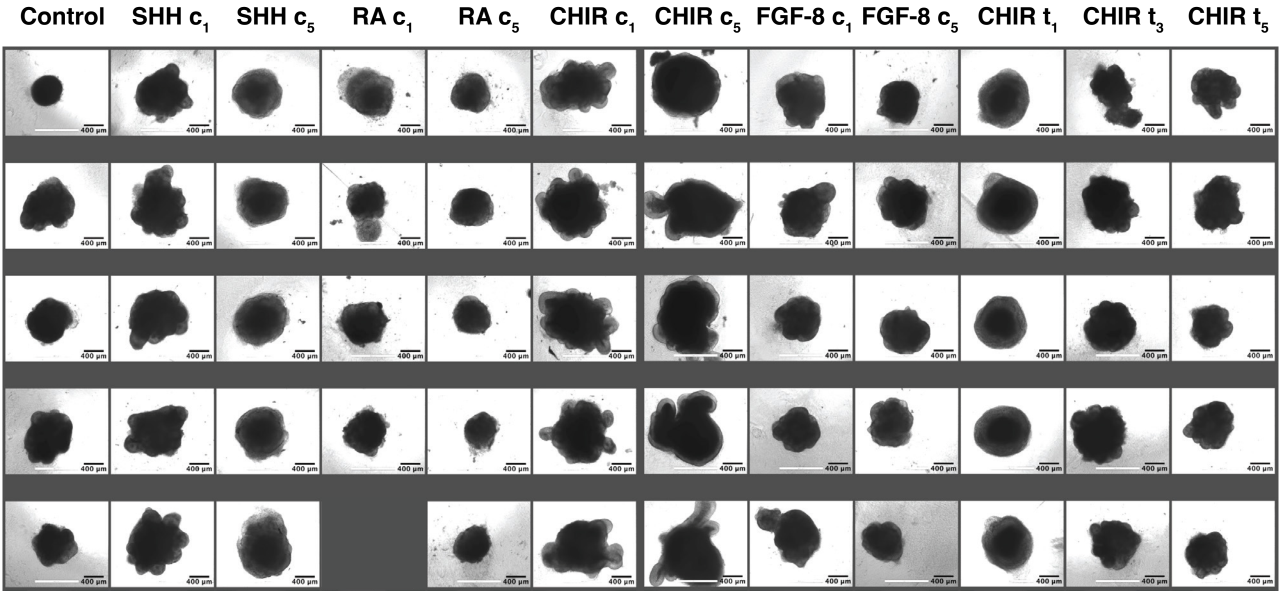

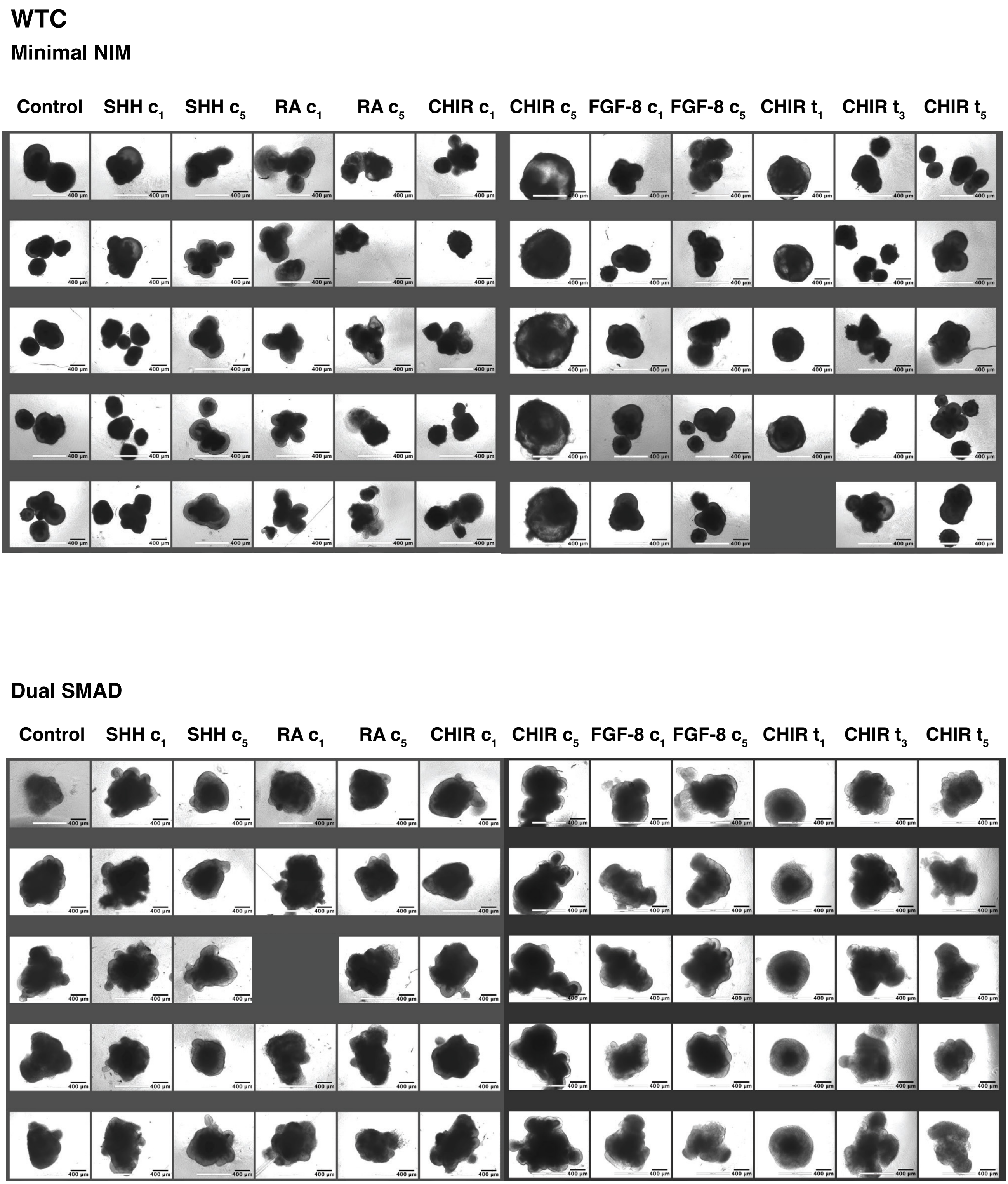
WTC
Minimal NIM
Control
SHH c_1
SHH c_5
RA c_1
RA c_5
CHIR c_1
CHIR c_5
FGF-8 c_1
FGF-8 c_5
CHIR t_1
CHIR t_3
CHIR t_5
400 µm
Dual SMAD
Control
SHH c_1
SHH c_5
RA c_1
RA c_5
CHIR c_1
CHIR c_5
FGF-8 c_1
FGF-8 c_5
CHIR t_1
CHIR t_3
CHIR t_5
400 µm
