# Supplementary material for: Systematic scRNA-seq screens profile neural organoid response to morphogens
Source: Nat Methods. 2025 Dec 15;23(2):465–78. doi: 10.1038/s41592-025-02927-5 (PMC12904787; doi:10.1038/s41592-025-02927-5)
Supplement: Supplementary file 2 — Reporting Summary [file 41592_2025_2927_MOESM2_ESM.pdf]

nature portfolio

Corresponding author(s): Barbara Treutlein (barbara.treutlein@bsse.ethz.ch), Gray Camp (jarrettgrayson.camp@unibas.ch)

Last updated by author(s): 16/09/2025

Reporting Summary

Nature Portfolio wishes to improve the reproducibility of the work that we publish. This form provides structure for consistency and transparency in reporting. For further information on Nature Portfolio policies, see our Editorial Policies and the Editorial Policy Checklist.

Statistics

For all statistical analyses, confirm that the following items are present in the figure legend, table legend, main text, or Methods section.

n/a	Confirmed	
☐	☒	The exact sample size (*n*) for each experimental group/condition, given as a discrete number and unit of measurement
☐	☒	A statement on whether measurements were taken from distinct samples or whether the same sample was measured repeatedly
☐	☒	The statistical test(s) used AND whether they are one- or two-sided<br>*Only common tests should be described solely by name; describe more complex techniques in the Methods section.*
☐	☒	A description of all covariates tested
☐	☒	A description of any assumptions or corrections, such as tests of normality and adjustment for multiple comparisons
☐	☒	A full description of the statistical parameters including central tendency (e.g. means) or other basic estimates (e.g. regression coefficient) AND variation (e.g. standard deviation) or associated estimates of uncertainty (e.g. confidence intervals)
☐	☒	For null hypothesis testing, the test statistic (e.g. *F*, *t*, *r*) with confidence intervals, effect sizes, degrees of freedom and *P* value noted<br>*Give P values as exact values whenever suitable.*
☒	☐	For Bayesian analysis, information on the choice of priors and Markov chain Monte Carlo settings
☒	☐	For hierarchical and complex designs, identification of the appropriate level for tests and full reporting of outcomes
☐	☒	Estimates of effect sizes (e.g. Cohen's *d*, Pearson's *r*), indicating how they were calculated

Our web collection on statistics for biologists contains articles on many of the points above.

Software and code

Policy information about availability of computer code

Data collection	For the collection of light-sheet live imaging data, the Viventis LS1 Live software (Viventis Microscopy) was used. For time-course imaging of MiSTR-like neural progenitors, a Nikon Ti2 with spinning disk module and NIS Elements software was used. HCR imaging data was collected with a Zeiss LSM980 microscope using the ZEN Blue software (v3.5.093.00008).
Data analysis	Scripts reproducing the main analyses in this study are available in Github (https://github.com/quadbio/organoid_patterning_screen). Image analysis was performed using Fiji (Image J2, version 2.14.0/1.54f)

For manuscripts utilizing custom algorithms or software that are central to the research but not yet described in published literature, software must be made available to editors and reviewers. We strongly encourage code deposition in a community repository (e.g. GitHub). See the Nature Portfolio guidelines for submitting code & software for further information.

Data

Policy information about availability of data

All manuscripts must include a data availability statement. This statement should provide the following information, where applicable:

- Accession codes, unique identifiers, or web links for publicly available datasets
- A description of any restrictions on data availability
- For clinical datasets or third party data, please ensure that the statement adheres to our policy

Raw and processed sequencing data are available at ArrayExpress. The accessions for the individual experiments are E-MTAB-15622 for morphogen reproducibility

screen and E-MTAB-15667 for morphogen patterning screen. Processed data and the VCF files for demultiplexing are available at Zenodo (10.5281/zenodo.17225179).

Human research participants

Policy information about studies involving human research participants and Sex and Gender in Research.

Reporting on sex and gender	This study includes data from five different hPSC lines, two of them with XX karyotype (HES3, H9, WIBJ2) and two of them with XY karyotype (WTC, H1). The sex of the PSC donor was not considered for study design nor data analysis.
Population characteristics	Population characteristics of the PSC donors were not taken into account in this study.
Recruitment	No human participants were recruited for this study.
Ethics oversight	Stem cell experiments with WTC, Wibj2 hiPSCs, H9, H1 and HES3 (NKX2.1GFP/w) hESCs were approved by the Bundesamt für Gesundheit (Swiss Health Federal Office) with disposition number 606.0000-1/31 / 19.018224; and by the Ethikkommission Nordwest- und Zentralschweiz (Ethics Commission for Northern and Central Switzerland).

Note that full information on the approval of the study protocol must also be provided in the manuscript.

Field-specific reporting

Please select the one below that is the best fit for your research. If you are not sure, read the appropriate sections before making your selection.

☒ Life sciences ☐ Behavioural & social sciences ☐ Ecological, evolutionary & environmental sciences

For a reference copy of the document with all sections, see nature.com/documents/nr-reporting-summary-flat.pdf

Life sciences study design

All studies must disclose on these points even when the disclosure is negative.

Sample size	No predetermined sample size calculation was performed. For comparison between morphogen conditions, n was determined as the number of cells from each condition passing QC controls. Minimum 3 organoids per condition per cell line were used.
Data exclusions	Cells not passing a QC control of <10% mitochondrial genes and >1000 genes detected were filtered out of the datasets.
Replication	Since organoid batches are subset to substantial variability in regional identities, cell type composition of each morphogen treatment was compared to that of a control condition that was not subject to any treatment, as a normalization method for batch effects. We replicated some of the morphogen treatment conditions, compared cell type enrichments versus control and generally found the same trends in cell-type enrichment. Whenever the trends were not consistent across replicates, we disregarded the effects of that particular morphogen treatment. While only HES3-derived organoids were exposed to all the morphogen treatments, we also evaluated the reproducibility of some treatments in organoids generated from other stem cell lines and report varying results in main Figure 4 and Extended Data Figure 7 and 9. We hypothesize that this variability stems from biological phenomena and highlight the need for further investigation in future studies.
Randomization	Experiments were not randomized. The allocation of developing organoids to experimental groups (morphogen conditions) was determined by their position in a 96-well plate. Usually, all organoids in the same column were allocated to the same morphogen treatment. To minimize positional effects, we avoided using the corners or edges of the plate (usually subject to culture artifacts) and included multiple control groups (one in each plate) within the same experiment.
Blinding	Investigators were not blinded to group allocation during data collection nor analysis. Since investigators were treating organoids with morphogens, it is impossible to perform the experiments with blinding. For scRNA-seq experiments organoids were randomly picked without any prescreening.

Reporting for specific materials, systems and methods

We require information from authors about some types of materials, experimental systems and methods used in many studies. Here, indicate whether each material, system or method listed is relevant to your study. If you are not sure if a list item applies to your research, read the appropriate section before selecting a response.

Materials & experimental systems

n/a	Involved in the study
☐	☒ Antibodies
☐	☒ Eukaryotic cell lines
☒	☐ Palaeontology and archaeology
☒	☐ Animals and other organisms
☒	☐ Clinical data
☒	☐ Dual use research of concern

Methods

n/a	Involved in the study
☒	☐ ChIP-seq
☒	☐ Flow cytometry
☒	☐ MRI-based neuroimaging

Antibodies

Antibodies used	See Extended Data Table 2 and Methods "Cell hashing (CITE-seq)"
Validation	Each lot of this antibody is quality control tested by Biolegend through immunofluorescent staining with flow cytometric analysis and the oligomer sequence is confirmed by sequencing.

Eukaryotic cell lines

Policy information about cell lines and Sex and Gender in Research

Cell line source(s)	WTC, WIBJ2, H1 and H9 cells were obtained from the Allen Institute for Brain Science, HipSci resource and WiCell repositories, while HES3 cells were shared by the Kirkeby lab upon MTA agreement with Prof. Ed Stanley and Prof. Andrew G. Elefanty (Murdoch Children's Research Institute, Melbourne). For details see Methods "Ethics statement " and "iPSC and ESC culture"
Authentication	For a subset of experiments (morphogen screen batches named "AT3", "OG1", "OG2", "SMG4", "SMOG1") cells were authenticated based on the comparison of single-cell RNAseq reads to reference single-nucleotide polymorphisms from each PSC line. For patterning reproducibility experiment authentication procedure see Methods "Demultiplexing of cell line identities".
Mycoplasma contamination	Regular PCR-based mycoplasma testing (Biological Industries) was performed to discard potential Mycoplasma infections.
Commonly misidentified lines (See ICLAC register)	None.
